# A maternal higher-complex carbohydrate diet increases bifidobacteria and alters early life acquisition of the infant microbiome in women with gestational diabetes mellitus

**DOI:** 10.3389/fendo.2022.921464

**Published:** 2022-07-28

**Authors:** Kameron Y. Sugino, Teri L. Hernandez, Linda A. Barbour, Jennifer M. Kofonow, Daniel N. Frank, Jacob E. Friedman

**Affiliations:** ^1^ Harold Hamm Diabetes Center, The University of Oklahoma Health Science Center, Oklahoma City, OK, United States; ^2^ Department of Medicine, Division of Endocrinology, Metabolism and Diabetes, The University of Colorado Anschutz Medical Center, Aurora, CO, United States; ^3^ College of Nursing, The University of Colorado Anschutz Medical Center, Aurora, CO, United States; ^4^ Department of Obstetrics and Gynecology, The University of Colorado Anschutz Medical Center, Aurora, CO, United States; ^5^ Department of Medicine, Division of Infectious Diseases, The University of Colorado Anschutz Medical Center, Aurora, CO, United States; ^6^ Department of Pathology, The University of Oklahoma Health Science Center, Oklahoma City, OK, United States

**Keywords:** gut microbiota, pregnancy, infant, glycemic control, metagenomics, GDM

## Abstract

Gestational diabetes mellitus (GDM) is associated with considerable imbalances in intestinal microbiota that may underlie pathological conditions in both mothers and infants. To more definitively identify these alterations, we evaluated the maternal and infant gut microbiota through the shotgun metagenomic analysis of a subset of stool specimens collected from a randomized, controlled trial in diet-controlled women with GDM. The women were fed either a CHOICE diet (60% complex carbohydrate/25% fat/15% protein, n=18) or a conventional diet (CONV, 40% complex carbohydrate/45% fat/15% protein, n=16) from 30 weeks’ gestation through delivery. In contrast to other published studies, we designed the study to minimize the influence of other dietary sources by providing all meals, which were eucaloric and similar in fiber content. At 30 and 37 weeks’ gestation, we collected maternal stool samples; performed the fasting measurements of glucose, glycerol, insulin, free fatty acids, and triglycerides; and administered an oral glucose tolerance test (OGTT) to measure glucose clearance and insulin response. Infant stool samples were collected at 2 weeks, 2 months, and 4–5 months of age. Maternal glucose was controlled to conventional targets in both diets, with no differences in Homeostatic Model Assessment of Insulin Resistance (HOMA-IR). No differences in maternal alpha or beta diversity between the two diets from baseline to 37 weeks’ gestation were observed. However, women on CHOICE diet had higher levels of *Bifidobacteriaceae*, specifically *Bifidobacterium adolescentis*, compared with women on CONV. Species-level taxa varied significantly with fasting glycerol, fasting glucose, and glucose AUC after the OGTT challenge. Maternal diet significantly impacted the patterns of infant colonization over the first 4 months of life, with CHOICE infants showing increased microbiome alpha diversity (richness), greater *Clostridiaceae*, and decreased *Enterococcaceae* over time. Overall, these results suggest that an isocaloric GDM diet containing greater complex carbohydrates with reduced fat leads to an ostensibly beneficial effect on the maternal microbiome, improved infant gut microbiome diversity, and reduced opportunistic pathogens capable of playing a role in obesity and immune system development. These results highlight the critical role a maternal diet has in shaping the maternal and infant microbiome in women with GDM.

## Introduction

Gestational diabetes mellitus (GDM), defined as glucose intolerance that arises during pregnancy, has increased worldwide over the past decade, reaching up to 20% of all pregnancies depending on the diagnostic criteria (1). GDM is associated with increased risk for preeclampsia, cesarean section delivery, and preterm birth (2), with up to 50% of women developing type 2 diabetes within 10 years. Infants born to women with GDM have a higher risk of developing type 2 diabetes, obesity, and other chronic inflammatory diseases (3).

Diet is the primary treatment for GDM, and accordingly, the dynamic interactions between GDM and the maternal and infant gut microbiome have received substantial attention. The composition of the gut microbiota is altered in women with GDM during late pregnancy (4–6) and is associated in some cases with changes in blood glucose levels (7). Women who develop GDM undergo shifts in gut microbiota composition characterized by an enrichment of potential opportunistic pathogens in the family *Enterobacteriaceae*, while beneficial bacteria like *Bifidobacterium* or *Lachnospiraceae* are depleted (8). Higher abundances of *Enterobacteriaceae* have been linked to prediabetes and untreated type 2 diabetes (9) and increased circulating levels of lipopolysaccharide in adults, which can promote the development of obesity and insulin resistance by inducing a chronic inflammatory state (10). On the other hand, *Bifidobacterium* and *Lachnospiraceae* produce short-chain fatty acids (SCFAs), such as acetate, propionate, and butyrate, which help to decrease the production of pro-inflammatory cytokines and control the overgrowth of *Enterobacteriaceae* in adults (11).

Further, maternal and early life exposures during pregnancy and lactation, such as maternal obesity or diabetes, influence the development of the infant microbiome and have an impact on the occurrence of common diseases, such as diabetes, allergic and atopic disease, cardiovascular disease, and obesity in the offspring (12). Studies on the neonatal microbiome report lower richness and differences in microbial composition in infants born to women with GDM compared with women without a GDM diagnosis (13–16). However, to our knowledge, only one study has investigated the impact of GDM on the infant microbiome beyond 2 weeks of age (16) and no studies have investigated this paradigm between 2 weeks and 6 months of age. Evidence suggests that the microbiome within the first 2 months of life impacts immune cell development patterns up to 3 months of age (17), and others found an association between increased *Lachnospiraceae* abundance at 3–4 months of age and childhood overweight/obesity at 1 and 3 years (18). Therefore, a need exists for studies that investigate the microbiome of infants born to women with GDM within the first 4 months of life.

Although diet is a major driver of microbiota composition and functions (19), the impact of maternal diet therapy in women with GDM on the maternal and infant microbiome and potential consequences of diet therapy remains unexplored. Diet therapy for women with GDM conventionally involves lower carbohydrate intake to blunt postprandial glucose excursion, at the cost of increasing dietary fats (20) since protein intake is typically constant and difficult to appreciably modify. A maternal high-fat diet, especially with an increase in saturated fats, promotes insulin resistance in human and animal models (20, 21), can lead to increased fetal fat accretion (22, 23), and may put the offspring at a higher risk of developing metabolic syndrome due to excess lipid exposure *in utero* (24, 25). Thus, alternate diets are needed to improve maternal/infant health in women with GDM without the unintended consequences that may arise from administering a high-fat CONV diet. From a microbiome perspective, a need for more highly controlled diet studies (26) is warranted to investigate the effects of dietary intervention in women with GDM and how diet impacts the maternal and infant microbiome and determine whether these alterations to the microbiome are linked to improvements in maternal and infant health outcomes. However, a previous study examining the maternal dietary effect on the maternal and infant microbiome in GDM had confounding influences of dietary intake that were not controlled (14). Here, we conducted a randomized, controlled dietary intervention (RCT) in GDM, directly comparing a conventional CONV diet or a diet higher in complex carbohydrates and lower in fat [CHOICE, Choosing Healthy Options in Carbohydrate Energy (27–29)]. Notably, all meals were provided for the duration of the study. The isocaloric nature of the diet was critical to ensure no differences in gestational weight gain (GWG) between diets. Furthermore, the percentage of saturated, polyunsaturated, and monounsaturated fats were identical and both simple sugars and fiber were also well controlled.

In the larger RCT of 46 women and infants who completed the study, we previously found that between-group maternal glycemic profiles were nearly identical (29). Although fasting free fatty acid (FFA) profiles were not different between the diet groups, the postprandial FFA profiles were lower with the CHOICE diet (29), likely suppressed by a higher postprandial insulin response. Between the diet groups, GWG was not different and insulin resistance indices were similar at 37 weeks, and the usual increase in the insulin resistance of pregnancy was blunted on both diets (29). Infant adiposity by air displacement plethysmography (PEAPOD), the primary endpoint on which the RCT was powered, was also not different, and cord blood glucose, C-peptide, FFAs, and triglycerides (TGs) were similar (29). From the larger RCT of 46 women, we collected a subset of stool samples in 34 women at 30 and 37 weeks’ gestation and in 24 infants at 2 weeks, 2 months, and 4–5 months of age to examine the impact of a controlled, third-trimester diet on the maternal microbiome and on infant microbial colonization during the first 4 months of life. We hypothesized that the consumption of the CHOICE, compared with CONV, would favorably alter maternal and infant microbiome composition consistent with improvements to the maternal measures of glucose tolerance.

## Materials and methods

### Participants

This study was approved by the Colorado Multiple Internal Review Board and was registered at http://www.clinicaltrials.gov (NCT02244814). All studies were performed in accordance with relevant guidelines and regulations. GDM was diagnosed using the Carpenter and Coustan criteria (30) between gestational weeks 24 and 28. Women were randomized to a diet group and entry criteria included the age of 20–36 years, a BMI of 26–39 kg/m^2^, a singleton pregnancy, no significant or obstetric comorbidities, no history of preterm labor or preeclampsia prior to term, and the treatment of GDM with diet alone. Informed consent was obtained from all women, and all women planned to breastfeed for at least 4 months and were otherwise healthy. Participants were excluded if they met any criteria for overt diabetes or were likely to fail the diet and require medical attention (fasting glucose >115 mg/dl or fasting TGs >400 mg/dl, which would place them at risk for TG-induced pancreatitis). Women taking beta blockers, antihypertensives, or glucocorticoids were excluded as were smokers and non-English speaking women. Maternal stool samples were excluded if they took antibiotics within 4 weeks of the sampling time. Infant stool samples were excluded if their mother received antibiotics at delivery, if they took antibiotics within 4 weeks of the stool sample collection, or if they were missing information on antibiotic use at the sample collection. From the original cohort of 46 women with GDM, 34 women in this subgroup had complete measurement data and stool samples from the study visits at 30 and 37 weeks’ gestation (n=16, CONV; n=18, CHOICE). Twenty-four infants had stool samples collected (n=14, CONV; n=10, CHOICE).

### Study protocol

The enrolled women with newly diagnosed GDM began dietary intervention between gestational weeks 30 and 31 and continued to delivery. Detailed information on the diets has been described (28). Briefly, diets were eucaloric, contained similar amounts of fiber (~23.5 and ~29.3 g/day for CONV and CHOICE, respectively), and had the following macronutrient distributions: CONV, 40% complex carbohydrate/45% fat/15% protein; CHOICE, 60% complex carbohydrate/25% fat/15% protein. Both diets were matched for fat percentage (35% saturated fatty acids/45% monounsaturated fatty acids/20% polyunsaturated fatty acids) and simple sugars (≤18% of kilocalories) and were composed of foods with a low-to-moderate glycemic index. Daily kilocalories were distributed as 25% breakfast/25% lunch/30% dinner/20% snacks. We defined complex carbohydrates as “polysaccharides and starches primarily derived from grains, vegetables, and fruits that tend to attenuate a sharp postprandial rise in plasma glucose” (28). All menus were tailored to individual participant food preferences, and meals were prepared by the Clinical Translational Research Center Nutrition Services at University of Colorado Anschutz Medical Campus. Meals were picked up by the participants or delivered every 72 h when they met with investigators. Women were provided with formula to supplement or replace breastfeeding when necessary (Gerber Good Start, Arlington, VA, USA). **Supplementary Figure 1** shows a summary of the study design.

### Blood measures

Maternal blood measures were performed at two different visits at 30–31 and 36–37 weeks’ gestation. A fasting (10-h) blood sample was collected prior to a 2-h oral glucose tolerance test (OGTT) at baseline (30–31 weeks) and again after 6–7 weeks on the diet (36–37 weeks). OGTT measurements were taken at 0, 30, 60, 90, and 120 min using a peripheral intravenous line. The Matsuda index was calculated using the standard method (31). HOMA-IR was calculated as [fasting insulin x (fasting glucose/18)]/22.5. Hepatic insulin resistance was calculated by multiplying glucose AUC by insulin AUC calculated during the first 30 min of the OGTT. GWG was determined by the change in weight from the first prenatal visit to delivery and weight gain on diet by the change in weight from time of diet randomization to delivery. Additional blood measures (i.e., TGs, FFAs, glycerol, glucose, C-peptide, and insulin) were performed at breakfast meal studies at baseline and 36–37 weeks’ gestation, where participants consumed a standardized breakfast meal (30% of total daily energy intake) after an overnight fast (≥10 h) as previously (28). When possible, cord blood was obtained to measure infant C-peptide, glucose, insulin, and HOMA-IR.

### Infant measurements

The infant breastfeeding status was determined by a questionnaire and was grouped by whether they were exclusively breastfed (yes, no, or mixed). The delivery mode was recorded as vaginal, cesarean section with labor, or cesarean section without labor; however, we grouped the cesarean section variables together due to low sample size. Birth weight and infant anthropometrics were obtained and newborn percent body fat was measured by air displacement plethysmography (PEAPOD) at delivery and the 2-week, 2-month, and 4–5 month postpartum visits (COSMED, Rome, Italy) (28).

### Stool sample collection and metagenomics processing

Maternal stool samples were collected at 30–31 and 36–37 weeks’ gestation by mothers within 24 h of their clinic visit and stored at -20°C until delivery to the laboratory, whereupon aliquots were separated and stored at -80°C until analysis. Infant stool samples were obtained at 2 weeks, 2 months, and 4–5 months, and DNA extraction was carried out as previously described using the QIAamp PowerFecal DNA kit (Qiagen Inc, Carlsbad, CA, USA). Triplicate shotgun metagenomic libraries were constructed for each stool sample (except for one pair of maternal samples, which was sequenced in duplicate) using the plexWell LP384 kit (seqWell Inc., Beverly, MA, USA) following the manufacturer’s protocol. The shotgun metagenomic sequencing of the pooled libraries was performed on the NovaSeq 6000 (Illumina, San Diego, CA, USA) at 2 × 150-bp read length by Novogene Inc. (Sacramento, CA, USA). Raw sequence reads were trimmed and processed for quality using BBMap (32). Briefly, the default options in bbduk were used to remove the adapter sequence and quality trimming before removing contaminant sequences with the Kmer filtering option. The tadpole command was then used in the error correction mode using default parameters. The triplicate/duplicate runs were then concatenated for further processing. The average sequencing depth, average number of contigs, and average contig length are shown in **Supplementary Table 1**. Metaphlan2 was used to retrieve the taxonomy and relative abundances using default settings (33). The participants’ sequence libraries at all timepoints were further concatenated for contig assembly using MEGAHIT (34). Contigs ≥1,000 bp were mapped to the MEGAHIT coassembly using bowtie2 (35). Contigs were predicted for gene function with Prodigal (36) using default settings and annotated to the Kyoto Encyclopedia of Genes (KEGG) database (37–39) using KofamScan (40). Reads per kilobyte million (RPKMs) were calculated for each annotation for downstream analysis.

### Statistical analysis

#### Population characteristics

Maternal BMI and blood measures were log-scaled then standardized using the mean and standard deviation; the normality of the data was checked using a Shapiro–Wilk test. To test for differences between the groups, a repeated-measures ANOVA was performed on the continuous variables against the diet group and time. Continuous infant characteristics were modeled using the same repeated-measures ANOVA procedure as for the maternal characteristics, and categorical variables were tested for differences between the diet groups using a chi-squared test.

#### Microbial analysis

Bacterial sequence counts were rarefied to 100,000 reads per sample, without replacement, 999 times and averaged across all sampling iterations before rounding to the nearest integer. Rarefaction curves were run to confirm that sample richness reached an asymptote within 100,000 reads and Good’s coverage was used to confirm adequate rarefaction (>99% for each sample) (41). Shannon diversity and Chao1 richness indices, calculated at the family, species, and gene annotation levels, were used as the measures of alpha diversity, and the Bray–Curtis dissimilarity index was used as a measure of beta diversity. Alpha and beta diversities were calculated using the vegan package (42).

#### Modeling

Models were constructed at the family level of taxonomy using alpha diversity, beta diversity, and taxonomic abundances—a deeper insight into species-level differences in taxonomic abundances was also calculated. Gene annotation abundances were investigated using the same modeling procedures. To evaluate these models, we used the function lmer from the lme4 package (43) for alpha diversity and the gene annotation data (linear mixed effects models), adonis2 from the vegan package for beta diversity (PERMANOVA),and glmmer.nb from the lme4 package for taxonomic abundance comparisons (negative binomial regression). For each dataset, modeling was performed as follows: 1) our simplest model included the diet group, sampling timepoint, and the interaction of diet group and sample timepoint within the framework of a repeated-measures design. 2) Additional models were built by adding a single explanatory variable to the simple model (12 additional models in the maternal analysis and four in the infant analysis). 3) A full model was constructed by adding all variables into one multivariate regression. The corrected Akaike information criterion (AICc) was computed for each of the models, where a lower AICc is indicative of a better model fit and an AICc score of 2 or lower suggests a significantly better model fit (44, 45). 4) If multiple explanatory variables from (2) had AICc scores 2 or lower than the simple model, the variables were added to a model together with group and time, and the AICc was calculated once more. 5) If there was a large difference between the AICc scores used in (4), the variable from (2) within the best-fitting model (i.e., the one with the lowest AICc) and variables in models within 2 AICc scores of the best-fitting model were included together in an additional model and the AICc again calculated. Once computed, the AICc scores were compared and the lowest AICc model was used for statistical analysis. If there were multiple models within 2 AICc points of each other, the simpler model of the two was used (e.g., if a model from (2) was close in AICc to our simple model (1), we would choose the simple model). If both models are equally parsimonious, the lower AICc model was chosen. If a model failed to converge or was found to be singular between the taxon and the variable of interest, the model was discarded.

Once the final models were calculated, the P-values were compiled and corrected for the false discovery rate (FDR) using the Benjamini–Hochberg procedure. AICc was calculated using the MuMIn package (46), except for the adonis2 models, which used a custom R script to calculate AICc from the residual sums of squares (47). In the negative binomial regression models, taxa at the family and species levels were included in the modeling if they were present in at least 60% of the samples and had at least 5,000 reads summed across all samples. The number of OTUS and percent read coverage after applying these filtering procedures are shown in **Supplementary Table 1**. To define the gene annotation based on mapping genes in the KEGG Orthology database, we used KEGG orthologous terms present in at least 60% of the samples, where they were then summed into gene families for further analysis using lmer.

## Results

### Maternal and infant characteristics were similar between the diet groups

Similar to the findings in the whole cohort (29), we found no differences in maternal characteristics between the diet groups in this subgroup (**Table 1**). The majority of infants in this subgroup were delivered vaginally (~70%) and exclusively breastfed throughout the collection period, and the body fat percentage was not different between the diet groups (29) (**Table 2**).

**Table 1:** Maternal population characteristics.

Group	CONV		CHOICE		p-value
Maternal age (years)^1^	32.96 ± 3.08		31.96 ± 4.77		0.49
Days on diet^1^	33.75 ± 4.17		33.0 ± 6.12		0.68
Weight gain on diet (kg)^1^	0.5 ± 1.43		0.62 ± 1.29		0.81
GWG (kg)^1^	8.9 ± 5.79		10.27 ± 4.47		0.44
Ethnicity, n (%)					0.21
Caucasian	11 (68.8)		16 (88.9)		
Asian	4 (26.7)		1 (5.6)		
American Indian or Alaska Native	1 (6.3)		0 (0)		
Other	0 (0)		1 (5.6)		
Parity, n (%)					0.19
0	6 (37.5)		12 (66.7)		
1	9 (56.3)		4 (22.2)		
2	1 (6.3)		1 (5.6)		
3	0 (0)		1 (5.6)		
**Timepoint**	**30 Weeks**	**37 Weeks**	**30 Weeks**	**37 Weeks**	
Sample Size, n	16	16	18	18	
Maternal BMI (kg/m²)^1^	31.47 ± 4.95	31.71 ± 4.86	32.93 ± 5.69	33.16 ± 5.79	0.94
Fasting Glucose (mg/dl)^1^	77.81 ± 7.17	73.56 ± 6.53	79.83 ± 6.49	72.72 ± 6.14	0.23
Fasting Insulin (uIU/ml)^1^	11.5 ± 5.89	11.62 ± 5.29	15.39 ± 6.57	13.67 ± 4.59	0.25
log2(Glucose AUC)^1^	14.19 ± 0.16	14.17 ± 0.17	14.14 ± 0.11	14.05 ± 0.14	0.11
log2(Insulin AUC)^1^	13.3 ± 0.9	13.44 ± 0.78	13.54 ± 0.65	13.64 ± 0.6	0.85
Fasting Free Fatty Acids^1^	8.79 ± 0.42	8.83 ± 0.32	8.85 ± 0.44	8.84 ± 0.43	0.74
Fasting Triglycerides^1^	198 ± 60.05	226.25 ± 56.43	216.78 ± 54.26	260.61 ± 58.89	0.27
Fasting Glycerol^1^	100.44 ± 34.78	102.38 ± 36.65	113.17 ± 29.48	126.44 ± 41	0.45
Hepatic IR^1^	22.07 ± 0.89	21.93 ± 0.78	22.27 ± 0.72	22.16 ± 0.62	0.84
HOMA IR^1^	2.23 ± 1.22	2.12 ± 1	3.08 ± 1.43	2.47 ± 0.9	0.16
Matsuda Index^1^	3.48 ± 1.89	3.4 ± 1.48	2.57 ± 0.87	2.77 ± 0.85	0.44

^1^Values shown are mean ± SD.

GWG, gestational weight gain.

**Table 2:** Infant population characteristics.

Group	CONV			CHOICE			p- value
Sample Size, n	14			10			
Vaginal Delivery, n (%)	11 (78.57)			6 (60.0)			0.59
Female, n (%)	7 (50.0)			6 (60.0)			0.94
Gestation Period (months)^1^	39.4 ± 0.3			38.9 ± 0.35			0.31
Birth Weight (g)^1^	3254 ± 101			3083 ± 119			0.29
% Body Fat at Delivery^1^	7.7 ± 7.5			7.5 ± 1.3			0.94
Cord C-peptide^1,a^	0.78 ± 0.23			0.86 ± 0.44			0.65
Cord Fasting Glucose^1,b^	58.25 ± 8.31			63.25 ± 11.32			0.40
Cord Fasting Insulin^1,c^	4.00 ± 1.00			3.75 ± 1.26			0.72
Ethnicity, n (%)							0.48
White	11 (78.6)			7 (70.0)			
Asian	3 (21.4)			2 (20.0)			
American Indian or Alaska Native	0 (0)			1 (10.0)			
**Infant Age**	**2 Weeks**	**2 Months**	**4–5 Months**	**2 Weeks**	**2 Months**	**4–5 Months**	
Sample Size, n	11	13	11	9	10	9	
Infant Weight (kg)	3.50 ± 0.36	5.24 ± 0.71	6.60 ± 0.78	3.23 ± 0.41	4.96 ± 0.51	6.32 ± 0.60	0.21
% Body Fat	10.39 ± 4.7	21.93 ± 4.0	25.19 ± 4.3	8.43 ± 4.3	18.37 ± 5.8	23.53 ± 3.9	0.24
Exclusively Breastfed, n (%)							0.71
No	1 (9.09)	1 (7.69)	2 (18.18)	0 (0)	1 (10.0)	2 (22.22)	
Mixed	3 (27.27)	3 (23.08)	2 (18.18)	0 (0)	1 (10.0)	1 (11.11)	
Yes	7 (63.64)	9 (69.23)	7 (63.64)	9 (100)	8 (80.0)	6 (66.67)	

^1^Values shown are mean ± SD.

a Missing 10 observations; CONV n=9, CHOICE n=5.

b Missing 12 observations; CONV n=8, CHOICE n=4.

c Missing 13 observations; CONV n=7, CHOICE n=4.

### Maternal microbial and gene-annotation diversity did not differ between the diet groups and were not associated with any maternal measures

We first evaluated whether the overall structure of the maternal microbiome was altered by the dietary intervention. To this end, we assessed the significance of alpha- and beta-diversity measures from maternal stool samples at the family, species, and gene annotation levels. At the family level, both alpha diversity metrics—Shannon diversity and Chao1 (richness)—were best modeled by the simplest model, which included only the diet group, time, and their interaction. However, none of these variables were significantly associated with either alpha diversity metric (p>0.05) (**Supplementary Figure 2**). At the species level, Shannon diversity was best modeled by the simplest model, but no terms were significant (p>0.05) (**Supplementary Figure 3**). Chao1 was best modeled by the full model, which included the diet group, time, and their interaction as well as all 11 of the maternal measurements. However, none of these variables were significantly associated with Chao1 richness at the species level (p>0.05). At the gene annotation level, we found results similar to those of the microbiome at the species level, where Shannon diversity was best modeled by the simplest model, but no terms were significant (p>0.05), and Chao1 was best modeled by the full model, but no terms were significant (p>0.05) (**Supplementary Figure 4**).

In the family level, beta-diversity models, the simplest model fit the data best, but the diet group, time, and their interaction were not associated with maternal microbiome beta diversity (p>0.05) (**Supplementary Figure 5**). Similar results were found at the species (simplest model, all p>0.05) and gene annotation levels (full model, all p>0.05) (**Supplementary Figures 6 and 7**).

#### Maternal microbiome displayed moderate associations with diet and maternal measures at the family level

Next, we identified individual taxa with differential abundances in maternal microbiota at the family level and found several taxa associated with maternal measures (**Table 3** and **Supplementary Figure 8**). We found that *Rikenellaceae* was negatively associated with maternal fasting FFA levels (p=0.02). Other associations were found between *Bifidobacteriaceae* and insulin AUC and an increase in *Bifidobacteriaceae* abundance over time on CHOICE but a decrease on CONV. However, these associations did not survive FDR correction. Likewise, we found that *Prevotellaceae* abundance was positively associated with the Matsuda index and *Erysipelotrichaceae* was similarly associated with HOMA-IR, but these associations did not survive FDR correction. A full list of models and their respective results are available in **Supplementary Table 2**.

**Table 3:** Variables associated with maternal family-level taxa determined by negative binomial regression.

Variable/Taxa					p-value	Adjusted p-value
Timepoint^1^	30 weeks	37 weeks				
*Bifidobacteriaceae*	1.91 ± 0.38	2.04 ± 0.4			0.03	0.34
Diet Group by Timepoint^1^	CONV 30W	CONV 37W	CHOICE 30W	CHOICE 37W		
*Bifidobacteriaceae*	2.07 ± 0.59	1.46 ± 0.44	1.76 ± 0.48	2.85 ± 0.73	0.004	0.12
Matsuda Index^2^	Predicted Slope					
*Prevotellaceae*	0.47 ± 0.27				0.01	0.19
log2(Fasting FFAs)^2^						
*Rikenellaceae*	-0.002 ± 0.001				<0.001	0.02
HOMA-IR^2^						
*Erysipelotrichaceae*	0.22 ± 0.21				0.02	0.24
log2(Insulin AUC)^3^						
*Bifidobacteriaceae*	0.22 ± 0.21				0.01	0.19

^1^Values shown are mean ± SEM.

^2^Number refers to the change in relative abundance per 1 unit increase in the variable being reported.

^3^Number refers to the change in relative abundance per 1 unit increase in the log-adjusted variable being reported.

#### CHOICE diet increased maternal abundance of *Bifidobacterium adolescentis*

At the species level, we found that several taxa were associated with maternal diet and other maternal measures (**Table 4** and **Supplementary Figure 9**). *Bifidobacterium adolescentis* was the main species responsible for the *Bifidobacteriaceae* increase on CHOICE and decrease on CONV (p=0.02). Similarly, *Prevotella copri* showed a trend decrease on CHOICE but increase on CONV (p=0.07). Two species of *Alistipes* were differentially associated with fasting glucose levels, with *A. finegoldii* displaying a negative relationship (p=0.04) while *A. shahii* had a positive relationship (p=0.03). *A. shaii*, *A. finegoldii*, *Alistipes putredinis*, and *Bacteroides vulgatus* were all negatively associated with fasting glycerol levels, but the association with *A. shahii* did not survive FDR correction. Four species, *Streptococcus thermophilus, Eubacterium ramulus, Dorea longicatena*, and *Roseburia hominis* were associated with glucose AUC after the OGTT challenge. However, only the association with *E. ramulus* was significant (p<0.001), while the others showed trend associations after FDR correction (p<0.1). A full list of models and their respective results are available in **Supplementary Table 3**.

**Table 4:** Variables associated with maternal species–level taxa determined by negative binomial regression.

Variable/Taxa					p-value	Adjusted p-value
Timepoint^1^	30 weeks	37 weeks				
*Bifidobacterium adolescentis*	0.58 ± 0.2	0.43 ± 0.16			0.01	0.12
*Eubacterium eligens*	0.33 ± 0.11	0.41 ± 0.12			0.04	0.22
*Anaerostipes hadrus*	0.14 ± 0.07	0.26 ± 0.09			0.05	0.24
*Dorea longicatena*	0.73 ± 0.17	0.75 ± 0.17			0.02	0.15
*Coprobacillus* unclassified	0.06 ± 0.04	0.12 ± 0.06			0.04	0.22
Diet Group^1^	CONV	CHOICE				
*Bifidobacterium longum*	1.07 ± 0.23	0.86 ± 0.2			0.03	0.19
*Bacteroides xylanisolvens*	0.3 ± 0.1	0.16 ± 0.07			0.03	0.19
*Clostridium symbiosum*	0.14 ± 0.08	0.03 ± 0.03			0.03	0.19
*Eubacterium hallii*	1.04 ± 0.22	2.52 ± 0.41			0.01	0.08
*Ruminococcus bromii*	0.17 ± 0.12	0.88 ± 0.49			0.02	0.15
*Klebsiella oxytoca*	0.03 ± 0.03	0.11 ± 0.06			0.03	0.20
Diet Group by Timepoint^1^	CONV 30W	CONV 37W	CHOICE 30W	CHOICE 37W		
*Bifidobacterium adolescentis*	0.54 ± 0.28^ab^	0.16 ± 0.1^b^	0.62 ± 0.28^ab^	1.17 ± 0.5^a^	0.001	0.02
*Prevotella copri*	0.19 ± 0.19	0.36 ± 0.35	0.14 ± 0.14	0.07 ± 0.08	0.01	0.07
*Eubacterium ramulus*	0.14 ± 0.09	0.18 ± 0.1	0.33 ± 0.14	0.24 ± 0.13	0.02	0.14
*Lachnospiraceae bacterium*	0.05 ± 0.06	0.03 ± 0.04	0.07 ± 0.06	0.13 ± 0.09	0.04	0.22
Matsuda index^2^	Predicted Slope					
*Bifidobacterium bifidum*	-0.57 ± 0.34				0.01	0.12
*Parabacteroides distasonis*	0.42 ± 0.23				0.01	0.09
Maternal BMI^2^						
*Bacteroides fragilis*	-0.15 ± 0.06				0.04	0.22
*Bacteroides thetaiotaomicron*	-0.1 ± 0.05				0.03	0.19
*Alistipes finegoldii*	-0.078 ± 0.065				0.02	0.15
HOMA-IR^2^						
*Bacteroides uniformis*	0.38 ± 0.16				0.004	0.07
Fasting Glucose^2^						
*Alistipes finegoldii*	-0.049 ± 0.044				0.002	0.04
*Alistipes shahii*	0.033 ± 0.032				0.001	0.03
*Anaerostipes hadrus*	0.04 ± 0.04				0.02	0.15
*Ruminococcus bromii*	-0.07 ± 0.03				0.005	0.07
log2(Fasting Triglycerides)^3^						
*Faecalibacterium prausnitzii*	-1.35 ± 0.29				0.01	0.12
log2(Fasting FFAs)^3^						
*Ruminococcus obeum*	-0.29 ± 0.35				0.02	0.13
*Escherichia coli*	0.83 ± 0.43				0.02	0.14
*Escherichia* unclassified	1.78 ± 0.83				0.01	0.08
log2(Fasting Glycerol)^3^						
*Bacteroides thetaiotaomicron*	-0.21 ± 0.24				0.01	0.12
*Bacteroides vulgatus*	-0.91 ± 0.28				<0.001	0.01
*Alistipes finegoldii*	-0.53 ± 0.52				<0.001	0.01
*Alistipes putredinis*	-0.53 ± 0.52				<0.001	0.01
*Alistipes shahii*	-0.38 ± 0.59				0.03	0.19
log2(Insulin AUC)^3^						
*Bacteroides fragilis*	-0.32 ± 0.35				0.01	0.11
*Eubacterium ventriosum*	1.2 ± 0.37				0.01	0.13
log2(Glucose AUC)^3^						
*Streptococcus thermophilus*	-3.33 ± 1.95				0.005	0.07
*Eubacterium ramulus*	3.56 ± 1.67				<0.001	<0.001
*Dorea longicatena*	1.67 ± 1.15				0.003	0.06
*Roseburia hominis*	-3.73 ± 1.4				0.003	0.06

Values in a row that do not contain the same superscript are significantly different, p<0.05

^1^Values shown are mean ± SEM.

^2^Number refers to the change in relative abundance per 1 unit increase in the variable being reported.

^3^Number refers to the change in relative abundance per 1 unit increase in the log-adjusted variable being reported.

#### Maternal gene annotation pathways were not altered by diet

Finally, we analyzed maternal microbiome gene annotations and found several associations between maternal measures and metabolic, transport, degradation, and infection-related pathways, but none of them survived FDR correction. A full list of models and their respective results are available in **Supplementary Table 4**.

#### Microbial family-level richness increased with age in infants born to women on CHOICE

**Figure 1:**
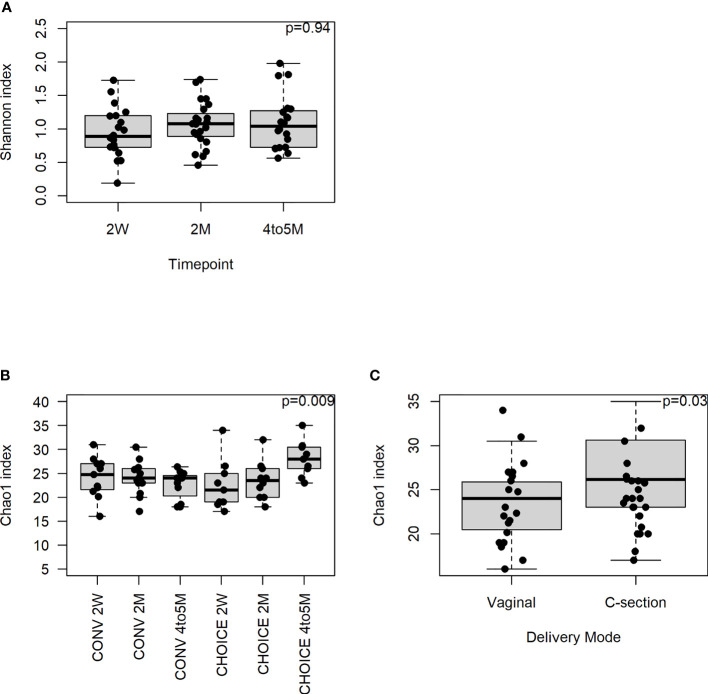
Infant microbiome alpha diversity at the family level. A repeated-measures mixed-effects model was used to test for statistical significance. **(A)** Association of Shannon diversity and infant age (n=20, 2-week; n=23, 2-month; n=20, 4–5-month). **(B)** Chao1 index association with diet group over time (n=11, CONV 2-week; n=13, CONV 2-month; n=11, CONV 4–5-month; n=9, CHOICE 2-week; n=10, CHOICE 2-month; n=9, CHOICE 4–5-month). **(C)** Association of Chao1 index with delivery mode (n=17, vaginal; n=7, cesarean).

Next, we evaluated the overall structure of the infant microbiome and whether it was altered by maternal dietary intervention. We assessed the significance of alpha- and beta-diversity measures in the infant stool samples at the family, species, and gene annotation levels. At the family level, we found no association between Shannon diversity and diet group, infant age, or the interaction of the two terms (**Figure 1A**). The infant microbiome Chao1 index decreased over time in CONV infants and increased in CHOICE infants (p=0.009), resulting in a significantly higher richness score in the CHOICE infants compared with CONV at 4–5 months (**Figure 1B**). Cesarean section delivery was associated with increased microbiome richness compared with infants born by vaginal delivery (**Figure 1C**). At the species level, Shannon diversity was associated with a trend increase over time (p=0.06), while the Chao1 index was not associated with any terms (p>0.05), as best modeled by the full model (diet, age, diet/age interaction and GWG, delivery mode, breastfeeding status, and sex) (**Supplementary Figure 10**). At the gene annotation level, results were similar to those found at the species level, and no terms were significant for the simplest model in Shannon diversity (p>0.05) or for the full model in the Chao1 index (p>0.05) (**Supplementary Figure 11**).

Bray–Curtis beta diversity at the family level was modeled best by the simple model and displayed a trend association with the diet group (p=0.06) and diet group by infant age (0.09) (**Supplementary Figure 12**). At the species level, we found a significant difference in the infant gut community between the diet groups (p=0.01) and how the microbiota developed over time (p=0.01) (**Supplementary Figure 13**) but not their interaction (p=0.18). Similar results were found at the gene annotation level but only reached trend significance (diet group and age, p=0.06, **Supplementary Figure 14**; diet and age interaction p=0.10).

#### Infant family-level abundances were altered with infant age, maternal diet group, and their interaction

Like we did with the maternal microbiome data, we identified individual taxa with differential abundances in the infant microbiota at the family level. Multiple taxa differed in abundance in association with maternal or infant variables (**Table 5** and **Supplementary Figure 15**). *Staphylococcaceae* and *Streptococcaceae* were enriched at 2 weeks of age and decreased in abundance over time at 2 months and 4–5 months of age (both p<0.001). *Ruminococcaceae* increased at 2 months and remained stable through 4–5 months of age (p<0.001), while *Veillonellaceae* also increased in abundance at 2 months but decreased at 4–5 months of age (p<0.001). *Coriobacteriaceae* and *Eubacteriaceae* increased in abundance over time (p=0.03 and p<0.001, respectively). Two families were associated with the diet group alone: CONV infants had higher levels of *Enterococcaceae* (p=0.002) and a lower abundance of *Clostridiaceae* (p=0.03).

**Table 5:** Variables associated with infant family-level taxa determined by negative binomial regression.

Variable/Taxa							p-value	Adjusted p-value
Infant Age	2 Weeks	2 Months	4-5 Months					
*Coriobacteriaceae*	0.02 ± 0.01^c^	0.13 ± 0.06^b^	0.51 ± 0.27^a^				0.005	0.03
*Staphylococcaceae*	0.91 ± 0.42^a^	0.05 ± 0.02^b^	0.01 ± 0.01^c^				<0.001	<0.001
*Streptococcaceae*	6.35 ± 1.98^a^	1.16 ± 0.34^b^	0.87 ± 0.27^b^				<0.001	<0.001
*Clostridiaceae*	0.54 ± 0.22	0.18 ± 0.07	0.3 ± 0.12				0.04	0.15
*Eubacteriaceae*	0.01 ± 0.002^b^	0.01 ± 0.003^b^	0.15 ± 0.04^a^				<0.001	<0.001
*Ruminococcaceae*	0.04 ± 0.02^b^	0.15 ± 0.06^a^	0.2 ± 0.09^a^				<0.001	<0.001
*Verrucomicrobiaceae*	0.01 ± 0.004^c^	0.42 ± 0.14^a^	0.05 ± 0.02^b^				<0.001	<0.001
Diet Group	CONV	CHOICE						
*Enterococcaceae*	0.43 ± 0.13	0.07 ± 0.03					<0.001	0.002
*Clostridiaceae*	0.29 ± 0.16	0.4 ± 0.25					0.01	0.03
*Ruminococcaceae*	0.17 ± 0.08	0.06 ± 0.04					0.05	0.16
*Veillonellaceae*	4.49 ± 2.32	1.55 ± 0.94					0.03	0.12
Diet Group by Infant Age	CONV 2W	CONV 2M	CONV 4-5M	CHOICE 2W	CHOICE 2M	CHOICE 4-5M		
*Clostridiaceae*	0.11 ± 0.07^ab^	0.45 ± 0.29^ab^	0.5 ± 0.34^ab^	1.84 ± 1.41^a^	0.09 ± 0.07^b^	0.39 ± 0.3^ab^	<0.001	<0.001
*Eubacteriaceae*	0.01 ± 0.003^b^	0.01 ± 0.003^b^	0.88 ± 0.34^a^	0.01 ± 0.003^b^	0.01 ± 0.01^b^	0.02 ± 0.01^b^	<0.001	<0.001
*Veillonellaceae*	6.59 ± 3.96^a^	5.37 ± 3.1^a^	2.56 ± 1.54^ab^	0.93 ± 0.64^b^	1.23 ± 0.83^ab^	3.24 ± 2.23^ab^	0.01	0.04
Delivery Mode	Vaginal	C-section						
*Lactobacillaceae*	0.12 ± 0.05	0.97 ± 0.53					0.002	0.01
*Eubacteriaceae*	0.02 ± 0.01	0.01 ± 0.01					0.03	0.12
Sex	Male	Female						
*Verrucomicrobiaceae*	0.02 ± 0.01	0.15 ± 0.04					<0.001	<0.001
GWG^1^	Predicted Slope							
*Enterococcaceae*	0.18 ± 0.04						0.01	0.04
*Lachnospiraceae*	0.48 ± 0.22						0.001	0.01

Values in a row that do not contain the same superscript are significantly different, p<0.05

All values shown are mean ± SEM.

^1^Number refers to the increase in relative abundance per 1 kg increase in gestational weight gain (GWG).

Several families were significantly associated with the diet group and infant age. *Clostridiaceae* began higher in CHOICE at 2 weeks of age and decreased at 2 months but remained stable in the CONV infants (p<0.001). *Eubacteriaceae* was stable in CHOICE infants but was enriched at 4–5 months in CONV infants (p<0.001) Finally, *Veillonellaceae* began at a high abundance in 2-week-old CONV infants and decreased over time, while this family was low in abundance in CHOICE infants at 2 weeks of age and increased in abundance over time (p=0.04).

Independent of the infant age, *Lactobacillaceae* was impacted by the delivery mode; infants delivered vaginally had a lower abundance than infants born *via* cesarean section (p=0.01). The abundance of *Verrucomicrobiaceae* was higher in female infants (p<0.001). *Enterococcaceae* and *Lachnospiraceae* were both positively associated with maternal GWG, where *Enterococcaceae* increased by 0.18% relative abundance for every 1 kg gained during pregnancy (p=0.04) and *Lachnospiraceae* increased by 0.48% relative abundance for every kilogram (p=0.006). A full list of models and their respective results are available in **Supplementary Table 5**.

#### Species-level abundances in infants change over time and displayed different colonization patterns between maternal diet groups

At the species level, *B. breve*, *B. longum*, *Collinsella aerofaciens*, *B. ovatus*, *B. thetaiotamicron*, *E. rectale*, *Faecalibacterium prausnitzii*, and *Subdoligranulum* unclassified increased in abundance from 2 weeks to 4–5 months (**Table 6** and **Supplementary Figure 16**). The opposite association was observed for *Staphylococcus epidermidis*, *S. anginosus*, *S. vestibularis*, *Clostridium perfringens*, *Veillonella dispar*, and *Haemophilus parainfluenza*, which decreased in abundance as the infant aged. *V. atypica* increased in abundance at 2 months of age before returning to the level similar to that seen at 2 weeks. Four species were associated with diet—*Enterococcus faecalis*, *S. anginosus*, *C. perfringens*, and *V. parvula*—all of which were higher in CONV infants independent of infant age.

**Table 6:** Variables associated with infant species–level taxa determined by negative binomial regression.

Variable/Taxa							p value	Adjusted p value
Infant Age	2 Weeks	2 Months	4-5 Months					
*Bifidobacterium adolescentis*	0.02 ± 0.01	0.04 ± 0.03	0.03 ± 0.02				0.036	0.151
*Bifidobacterium bifidum*	0.03 ± 0.01	0.03 ± 0.02	0.08 ± 0.04				0.028	0.125
*Bifidobacterium breve*	0.82 ± 0.46^b^	2.94 ± 1.62^a^	4.22 ± 2.37^a^				0.002	0.017
*Bifidobacterium longum*	0.55 ± 0.28^b^	1.58 ± 0.78^b^	8.02 ± 4.14^a^				0.002	0.017
*Collinsella aerofaciens*	0.002 ± 0.001^b^	0.003 ± 0.002^b^	0.02 ± 0.02^a^				0.001	0.011
*Bacteroides ovatus*	0.01 ± 0.01^b^	0.02 ± 0.01^b^	0.04 ± 0.02^a^				<0.001	<0.001
*Bacteroides thetaiotaomicron*	0.006 ± 0.003^b^	0.007 ± 0.004^b^	0.01 ± 0.008^a^				0.003	0.025
*Staphylococcus epidermidis*	0.15 ± 0.06^a^	0.002 ± 0.001^b^	0.001 ± 0.0005^b^				<0.001	<0.001
*Streptococcus anginosus*	0.015 ± 0.007^a^	0.003 ± 0.001^b^	0.001 ± 0.0005^b^				<0.001	<0.001
*Streptococcus vestibularis*	1.79 ± 0.88^a^	0.17 ± 0.08^b^	0.02 ± 0.01^c^				<0.001	0.002
*Clostridium perfringens*	1.0 ± 0.47^a^	0.21 ± 0.10^b^	0.11 ± 0.05^b^				0.016	0.075
*Eubacterium rectale*	0.008 ± 0.002^b^	0.01 ± 0.003^ab^	0.015 ± 0.005^a^				0.014	0.069
*Faecalibacterium prausnitzii*	0.007 ± 0.002^b^	0.009 ± 0.003^ab^	0.01 ± 0.004^a^				0.009	0.052
*Subdoligranulum* unclassified	0.02 ± 0.01^b^	0.05 ± 0.02^a^	0.09 ± 0.04^a^				<0.001	0.002
*Veillonella atypica*	0.02 ± 0.01^b^	0.05 ± 0.02^a^	0.03 ± 0.01^ab^				0.010	0.058
*Veillonella dispar*	0.04 ± 0.02^a^	0.03 ± 0.02^ab^	0.01 ± 0.01^b^				0.012	0.062
*Enterobacter cloacae*	0.03 ± 0.02	0.06 ± 0.03	0.03 ± 0.02				0.032	0.138
*Haemophilus parainfluenzae*	0.006 ± 0.006^ab^	0.009 ± 0.009^a^	0.002 ± 0.002^b^				0.017	0.078
Diet Group	CONV	CHOICE						
*Bifidobacterium longum*	2.26 ± 1.27	1.61 ± 1.06					0.045	0.177
*Enterococcus faecalis*	0.35 ± 0.11	0.05 ± 0.02					<0.001	0.001
*Streptococcus anginosus*	0.02 ± 0.008	0.0006 ± 0.0003					<0.001	<0.001
*Clostridium perfringens*	0.41 ± 0.15	0.2 ± 0.08					<0.001	<0.001
*Veillonella parvula*	1.06 ± 0.59	0.15 ± 0.1					<0.001	0.003
Diet Group by Infant Age	CONV 2W	CONV 2M	CONV 4-5M	CHOICE 2W	CHOICE 2M	CHOICE 4-5M		
*Bifidobacterium longum*	1.55 ± 1.06^ab^	0.94 ± 0.61^b^	7.89 ± 5.36^a^	0.19 ± 0.15^c^	2.63 ± 1.99^ab^	8.15 ± 6.34^ab^	0.003	0.022
*Bacteroides ovatus*	0.01 ± 0.01^b^	0.01 ± 0.01^b^	0.09 ± 0.06^a^	0.02 ± 0.02^ab^	0.02 ± 0.02^ab^	0.02 ± 0.02^ab^	0.004	0.028
*Enterococcus faecalis*	0.77 ± 0.42	0.27 ± 0.14	0.22 ± 0.12	0.03 ± 0.02	0.03 ± 0.02	0.15 ± 0.09	0.043	0.175
*Streptococcus anginosus*	0.57 ± 0.31^a^	0.01 ± 0.007^b^	0.001 ± 0.001^c^	0.0004 ± 0.0003^c^	0.0005 ± 0.0003^c^	0.001 ± 0.0007^c^	<0.001	<0.001
*Clostridium perfringens*	0.13 ± 0.08^c^	1.43 ± 0.84^ab^	0.36 ± 0.24^b^	7.78 ± 5.32^a^	0.03 ± 0.02^c^	0.03 ± 0.02^c^	<0.001	<0.001
*Veillonella parvula*	3.35 ± 2.8^a^	0.9 ± 0.64^ab^	0.4 ± 0.3^ab^	0.05 ± 0.04^b^	0.18 ± 0.15^ab^	0.36 ± 0.28^ab^	0.006	0.035
*Veillonella* unclassified	0.39 ± 0.36^ab^	0.49 ± 0.44^ab^	0.13 ± 0.12^b^	0.3 ± 0.32^ab^	0.34 ± 0.36^ab^	1.24 ± 1.31^a^	0.006	0.035
*Enterobacter cloacae*	0.05 ± 0.04^a^	0.05 ± 0.04^a^	0.01 ± 0.01^a^	0.02 ± 0.02^ab^	0.06 ± 0.06^a^	0.08 ± 0.07^a^	0.012	0.062
*Klebsiella* unclassified	0.01 ± 0.01	0.04 ± 0.04	0.03 ± 0.03	0.09 ± 0.1	0.02 ± 0.02	0.05 ± 0.06	0.027	0.124
Delivery Mode	Vaginal	C-section						
*Clostridium perfringens*	0.05 ± 0.01	1.82 ± 0.84					<0.001	<0.001
Sex	Male	Female						
*Streptococcus anginosus*	0.002 ± 0.0008	0.006 ± 0.003					0.012	0.062
Breastfeeding Status	No	Mixed	Exclusive					
*Staphylococcus epidermidis*	0.002 ± 0.002^b^	0.004 ± 0.002^b^	0.05 ± 0.01^a^				<0.001	<0.001
*Streptococcus anginosus*	0.003 ± 0.002^b^	0.0007 ± 0.0004^b^	0.02 ± 0.005^a^				<0.001	<0.001
GWG^1^	Predicted Slope							
*Enterococcus faecalis*	0.14 ± 0.03						<0.001	0.001
*Streptococcus salivarius*	0.42 ± 0.08						0.005	0.030
*Ruminococcus gnavus*	0.01 ± 0.003						<0.001	0.002

Values in a row that do not contain the same superscript are significantly different, p<0.05.

All values shown are mean ± SEM.

^1^Number refers to the increase in relative abundance per 1 kg increase in gestational weight gain (GWG).

Several species were associated with changes in abundance within a diet group over time. *B. ovatus*, *S. anginosus*, and *Enterobacter cloacae* displayed a pattern of abundance in CONV infants that changed over time (either up or down), whereas no change in the abundance of these species was observed in CHOICE infants. With *C. perfringens*, increased abundance was seen at 2 weeks of age in CHOICE infants and decreased by 2 months, whereas this species was depleted in CONV infants at 2 weeks and increased in abundance at 2 months. Similar differential colonization patterns over time between the diet groups were also seen for *B. longum*, *V. parvula*, and *Veillonella* unclassified.

*C. perfringens* was higher in abundance in infants born *via* cesarean section. *S. epidermidis* and *S. anginosus* were both higher in abundance in exclusively breastfed infants compared with mixed feeding and formula-only feeding. Female infants had a trend for higher abundance of *S. anginosus* compared with male infants. Three species were positively associated with GWG: *E. faecalis* increased by 0.14% relative abundance per 1 kg of weight gained, *S. salivarius* by 0.42%, and *R. gnavus* by 0.01%. A full list of models and their respective results are available in **Supplementary Table 6**.

#### Infant gene annotation pathways were not different between maternal diet groups

Finally, we analyzed infant microbiome gene annotations and found only one pathway that was significant with any of the tested maternal/infant variables after FDR correction. A trend between the mannose-type O-glycan biosynthesis pathway and the breastfeeding status was observed (p=0.09) where infants who were not breastfed had a much higher abundance of genes involved in this pathway than mixed feeding or exclusive breastfeeding (**Supplementary Figure 17**). A full list of models and their respective results are available in **Supplementary Table 7**.

## Discussion

To our knowledge, this is the first study to compare maternal and infant gut microbiome outcomes in a dietary intervention of two different diet compositions (all meals provided) in women with GDM. We ascertained how the diets impacted the maternal gut microbiome and the relationships with maternal metabolic characteristics and the infant microbiome under eucaloric diet conditions and equivalent GWG. Surprisingly, we found that the probiotic family *Bifidobacteriaceae*, specifically *B. adolescentis*, increased in the microbiota of women on the CHOICE diet. *Bifidobacteria* are generally beneficial bacteria that attenuate intestinal inflammation and dysbiosis, in part by enhancing SCFA production (7), inhibiting and reducing lipopolysaccharide-induced injury of the gut epithelium (48), and by metabolizing resistant starches such as human milk oligosaccharides and other complex carbohydrates like fructooligosaccharides and galactooligosaccharides (49). *B. adolescentis* abundance is correlated with lower HbA1c and basal insulin requirements (50), suggesting that it conveys an overall protective effect of the CHOICE diet on pancreatic β-cell function in women with GDM. Others have found similar increases in *Bifidobacteria* after supplementing adults with prediabetes with galactooligosaccharides for 12 weeks (51). Furthermore, other studies suggest that the supplementation of resistant starches in healthy adults also increases *Bifidobacteria* (52), while the depletion of dietary carbohydrates in a gluten-free diet results in a likewise depletion of *Bifidobacteria* (53). Together, this suggests that higher intake of complex carbohydrates in the diet, in pregnant and non-pregnant individuals, increases *Bifidobacteriaceae* abundance in the microbiome.

The maternal microbiome at the family and species levels also varied significantly with the fasting levels of FFAs, glycerol, and glucose AUC after the OGTT challenge. Specifically, we found a negative association between *Rikenellaceae* and fasting FFAs. Other studies in adults have found an inverse association between this family and the levels of visceral adipose tissue in elderly adults (54), a negative association with obesity (55), and depleted abundance in adults with non-alcoholic fatty liver disease (56). Interestingly, species within the genus *Alistipes*, part of the family *Rikenellaceae*, were inversely associated with fasting glycerol and fasting glucose levels but not FFAs. *A. finegoldii* and *A. putredinis* were negatively associated with fasting glycerol levels along with *B. vulgatus*. Blood glycerol levels are a measure of adipose tissue lipolysis and TGs (57) and feed directly into the gluconeogenesis pathway. Together, our data suggest that bacteria in the family *Rikenellaceae* help lower FFAs, but specific members of the family (i.e., *Alisitpes*) have a relationship with a different part of the same pathway, notably fasting glycerol and glucose levels.

*B. uniformis* was positively associated with HOMA-IR in our cohort, although this was only a trend association after FDR correction. Supplementation studies have found that *B. uniformis* can reduce body weight gain, liver TGs, and inflammation in the context of a high-fat diet in mice (58, 59). However, in humans with metabolic syndrome, increases in the stool levels of *B. uniformis* had no effect on body composition or insulin sensitivity (60). Pregnant women with type 1 diabetes have increased abundance of *B. uniformis* (61). More work needs to be done to elucidate the role of *B. uniformis* in diabetes risk.

Maternal diet significantly impacted the patterns of infant colonization over time, with CHOICE infants showing increased microbiome alpha diversity (richness), greater *Clostridiaceae*, and decreased *Enterococcaceae*. To our knowledge, there are only two studies that have investigated the impact of maternal diet on the infant microbiome in humans. Lundgren et al. (62) observed that increased maternal fruit and vegetable intake was associated with higher levels of *Streptococcus* and *Clostridium* and decreased abundances of *Enterobacteriaceae* in offspring at 6 weeks of age. Chu et al. (19) found that a maternal diet higher in fat was associated with greater *Enterococcus* abundance in the meconium and higher *Bacteroides* abundance in infants at 6 weeks of age. We found a similar result in our cohort, where *Enterococcaceae* abundance was increased in infants born to mothers on the higher-fat CONV diet. *Enterococcaceae*, like *E. faecalis*, is known to influence healthy intestinal immune system development, but the members of this family can also act as opportunistic pathogens (63). In healthy infants, this family decreased in abundance as other bacteria began to colonize and populate the gut, suggesting that early *Enterococcaceae* colonization is important for proper immune training but an overabundance in the early life could increase the risk of *Enterococcus* infection.

The abundances of infant microbiome species were impacted by the maternal diet group as the infant aged. For example, *C. perfringens* was enriched in the gut of CONV infants; a relative abundance of 1.43% and 0.36% was observed at 2 months and 4–5 months of age, respectively. In contrast, *C. perfringens* started at a higher abundance at 2 weeks of age in CHOICE infants before dropping to 0.03% relative abundance at 2 and 4–5 months. Other groups have associated cesarean section delivery with a higher abundance of *C. perfringens* (64), which is consistent with our results. Moreover, the microbiome of germ-free mice colonized with gut microbes from infants born to women with obesity was enriched in *Clostridia* (65) and the enrichment of this taxa has been associated with adolescent obesity (66). This suggests that the higher-fat CONV diet exacerbated obesity-associated microbiome signals within a population that is already at risk for adverse health events due to the maternal GDM status. We found an association between a higher abundance of *Lactobacillaceae* and cesarean section delivery, which is surprising considering that vaginally delivered infants have a much higher exposure to vaginal *Lactobacillus* species compared with cesarean section–delivered infants. However, a recent systematic review by Shaterian et al. concluded that cesarean section delivery is associated with a higher abundance of *Lactobacillus* in the first 3 months of life, while vaginally delivered infants have higher abundances of this taxa after 3 months of age (67), which matches our results. Moreover, gut *Lactobacillus* species do not appear to originate from the maternal vaginal microbiome (68), likely due to differences in species-specific adaptations needed to colonize the vagina versus the human gastrointestinal tract. Together, this suggests that the delivery mode impacts *Lactobacillaceae* colonization not because of the direct transmission of *Lactobacillus* from a mother to a child but because of differential successional patterns of colonization prompted by early bacterial exposures. In our other comparisons, we the family *Lachnospiraceae* was positively associated with maternal GWG and was predicted to increase in relative abundance by 0.5% for each kilogram increase in GWG. Other studies have noted a positive association between maternal overweight/obesity and infant *Lachnospiraceae* abundance (13, 18), which was linked to infant risk for overweight/obesity later in life (18). More studies are needed to determine whether individual genera/species within *Lachnospiraceae* are contributing to infant adiposity and whether specific metabolites produced by this family are responsible for this association with overweight/obesity risk in offspring.

Two species in infant stool were associated with the breastfeeding status: *S. epidermidis* and *S. anginosus*, both of which were enriched in the gut of infants who were exclusively breastfed. *S. epidermidis* is acquired from skin and can be found in breastmilk, may play a role in educating the immune system and can act as an opportunistic pathogen in neonates (69). Less is known about the role of *S. anginosus* in the commensal human gut, although it has been shown to be a common member of the skin, respiratory, and intestinal microbiomes and has the potential to cause infection (70). Breastfeeding for the first 6 months of life is the recommended practice to optimally support infant survival, nutrition, and development (71). Our results suggest that exclusive breastfeeding results in distinct communities in the infant gut and increases the abundance of bacteria important for immune training.

A major strength of our RCT study is that the mothers were well matched, the study design allowed for well-controlled dietary constituents that impact the microbiome (fiber, calories, all meals provided), and GWG was nearly identical. We also controlled for antibiotic exposure, excluding any maternal or infant stool sample when antibiotics were reported within 4 weeks of collection. Importantly, we collected maternal and infant stool samples at several timepoints, allowing us to interrogate how the maternal diet group related to changes in the microbiome across gestation and infant age. Another strength is that the gut microbiota of study participants was not confounded by anti-hyperglycemic agents. Our trial also had certain limitations, including the small sample size and the predominantly Caucasian participants, and thus needs confirmation in a larger RCT including women with different ethnicities. Additionally, due to limitations inherent in macronutrient research, we cannot be certain that the differences reported in women on CHOICE are due to a higher intake of carbohydrates rather than a lower intake of fats. However, we emphasize carbohydrate intake in this study since the administration of complex carbohydrates to women with GDM makes this study unique and provides an alternative to the higher-fat CONV GDM diet. Although the infant sample size was small, all results were corrected for FDR, and subspecies occurrence with divergent functionalities was identified with metagenomic sequencing. Many of the bacteria investigated were, on average, less than a percent of the total relative abundance in the participants’ microbiome. While the higher-abundance taxa will interact more with the host and gut environment, rare taxa have been shown to disproportionately contribute to shifts in a microbiome community over time (72) and may act as metabolic keystones to bridge gaps between the functional repertoires of the microbiome as a whole (73). The entire study cohort was powered to detect differences in postprandial FFA AUC, placental FATP2 expression, and neonatal adiposity (primary outcome) between groups. However, this subanalysis of the study trial may not be adequately powered to detect small differences in microbiota abundances and should thus be interpreted with caution. As this was an exploratory study, more work needs to be done in a larger cohort. In addition, although the two groups were extremely well matched for age, BMI, parity, and ethnic distribution (see **Table 1**), we cannot exclude other socioeconomic factors or unknown environment differences in the two cohorts. The goal of these studies was to recruit, to the greatest extent possible, women with similar starting metabolic characteristics, the ability to follow their diets (compliance), and without the need to go on to require insulin therapy. More work needs to be done to elucidate the roles of rare taxa in the maintenance of a healthy microbiome and the functional pathways in a larger cohort, despite their relative rarity.

Overall, our results show that an isocaloric diet high in complex carbohydrates and low in fat consumed by women with GDM is associated with a bacterial environment that is metabolically favorable, demonstrating greater bacterial diversity and a reduction in potential pathogens in infants during the first 4 months of life. Improving the gut microbiome diversity and reducing opportunistic pathogens capable of playing a role in obesity and immune system dysbiosis suggest the exciting possibility of using a targeted intervention to modulate these taxa and modify disease progression in a precision medicine–based approach in future studies. The potential implications of the abundance of these taxa for overall metabolic health across various phenotypes of infants from women with GDM await further elucidation.

## Data availability statement

The shotgun metagenomic data generated and analyzed during the current study are deposited in the NCBI Sequence Read Archive under BioProject ID PRJNA845806. https://www.ncbi.nlm.nih.gov/sra.

## Ethics statement

The studies involving human participants were reviewed and approved by Colorado Multiple Internal Review Board. The patients/participants provided their written informed consent to participate in this study.

## Author contributions

TH and LB designed the clinical study from which samples were generated. DF and JF provided guidance on the study design related to microbiome study data interpretation and analysis. JK processed stool samples for sequence acquisition. KS performed biostatistical and bioinformatic analyses. All authors contributed to and approved the manuscript.

## Funding

This study was supported by the National Institute of Diabetes, Digestive, and Kidney Diseases (R01 DK101659 to TH), American Diabetes Association/Glaxo Smith Kline Targeted Research Award (1-13-GSK-13), and Janssen Pharmaceuticals. The funders had no role in the design, conduct, or reporting of this work.

## Acknowledgments

The authors wish to thank the participants of the CHOICE study and Emily Dunn, Kristy Rolloff, Laurie Kay Moss, Nicole Hirsch, and Sarah Farabi at University of Colorado Anschutz Medical Campus for collection and storage of clinical samples. We thank Rachel Janssen for editing the manuscript.

## Conflict of interest

The authors declare no conflicts of interest.

## Publisher’s note

All claims expressed in this article are solely those of the authors and do not necessarily represent those of their affiliated organizations, or those of the publisher, the editors and the reviewers. Any product that may be evaluated in this article, or claim that may be made by its manufacturer, is not guaranteed or endorsed by the publisher.
