# Supplementary material for: A maternal higher-complex carbohydrate diet increases bifidobacteria and alters early life acquisition of the infant microbiome in women with gestational diabetes mellitus
Source: Front Endocrinol (Lausanne). 2022 Jul 28;13:921464. doi: 10.3389/fendo.2022.921464 (PMC9366142; doi:10.3389/fendo.2022.921464)
Supplement: Supplementary file 1 [file Image_1.pdf]

A Maternal Higher-complex Carbohydrate Diet Increases Bifidobacteria and Alters Early Life Acquisition of the Infant Microbiome in Women with Gestational Diabetes Mellitus

Kameron Y. Sugino[1], Teri L. Hernandez[2,3], Linda A. Barbour[2,4], Jennifer M. Kofonow[5], Daniel N. Frank[5], and Jacob E. Friedman[1,6]*

[1]Harold Hamm Diabetes Center, The University of Oklahoma Health Science Center, Oklahoma City, OK, United States; [2]Department of Medicine, Division of Endocrinology, Metabolism and Diabetes, [3]College of Nursing, [4]Department of Obstetrics and Gynecology, and [5]Department of Medicine, Division of Infectious Diseases, The University of Colorado Anschutz Medical Center, Aurora, CO, United States; [5]Department of Pathology, The University of Oklahoma Health Science Center, Oklahoma City, OK, United States

***Corresponding Author**: Jacob E Friedman, jed-friedman@ouhsc.edu

Supplementary figures

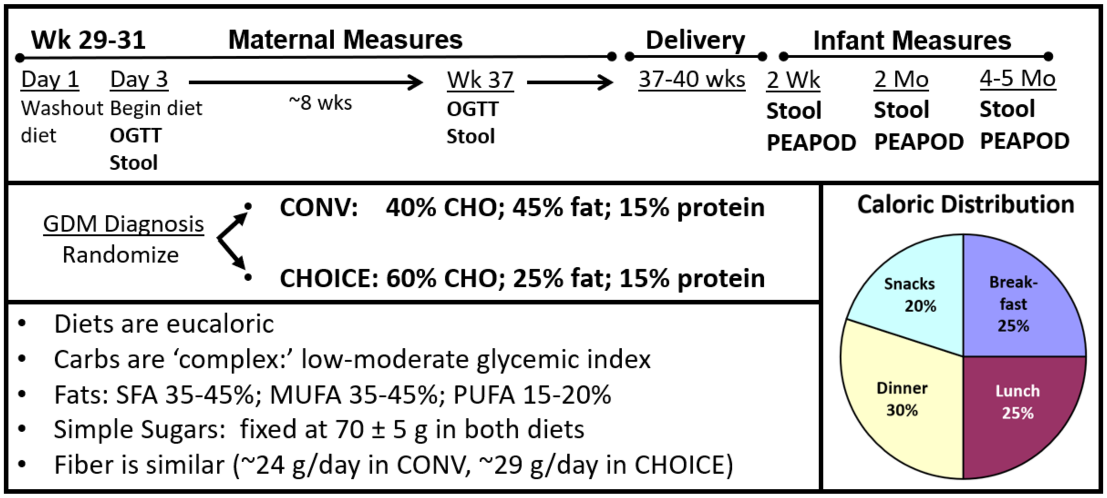

Supplementary Figure 1. Overview of the study design.

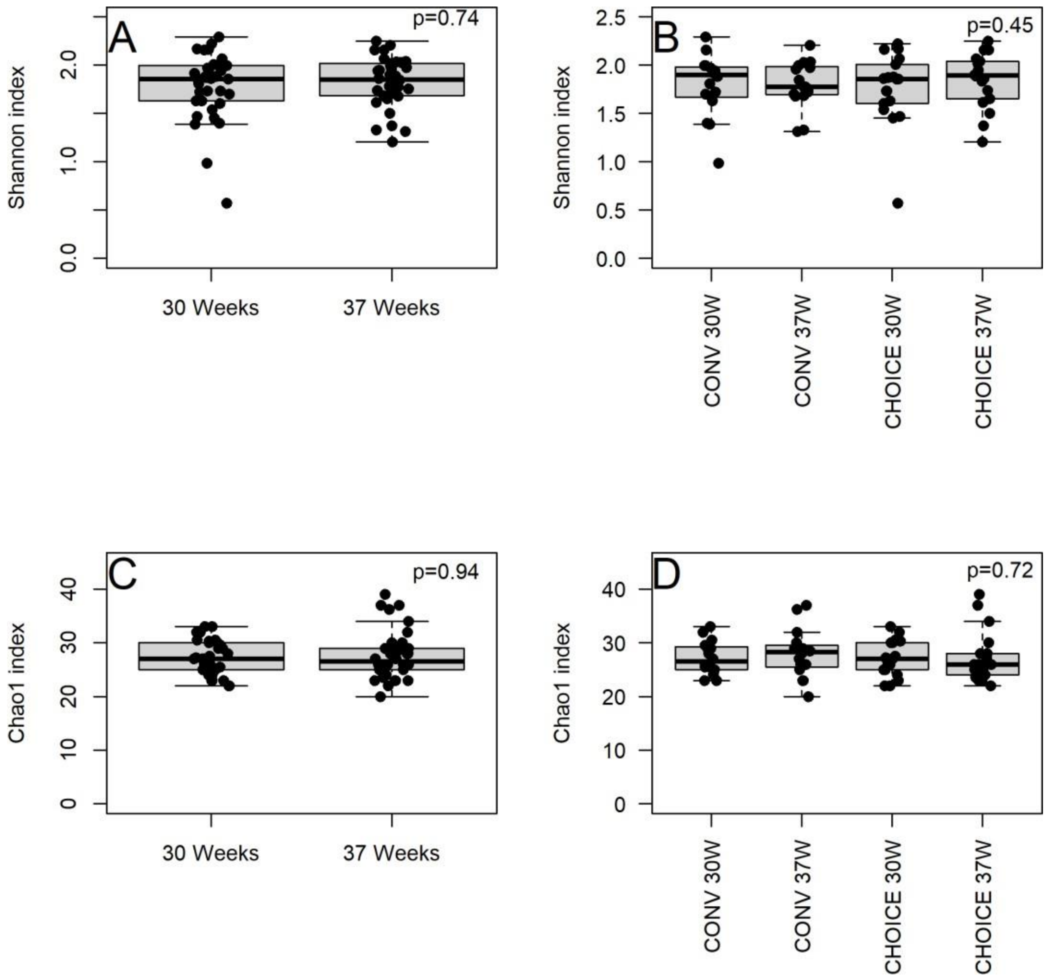

Supplementary Figure 2. Maternal microbiome alpha diversity at the family level. Shannon diversity for weeks of gestation (A) and the interaction between diet group and time (B). Chao1 index of richness for weeks of gestation (C) and the interaction between diet group and time (D).

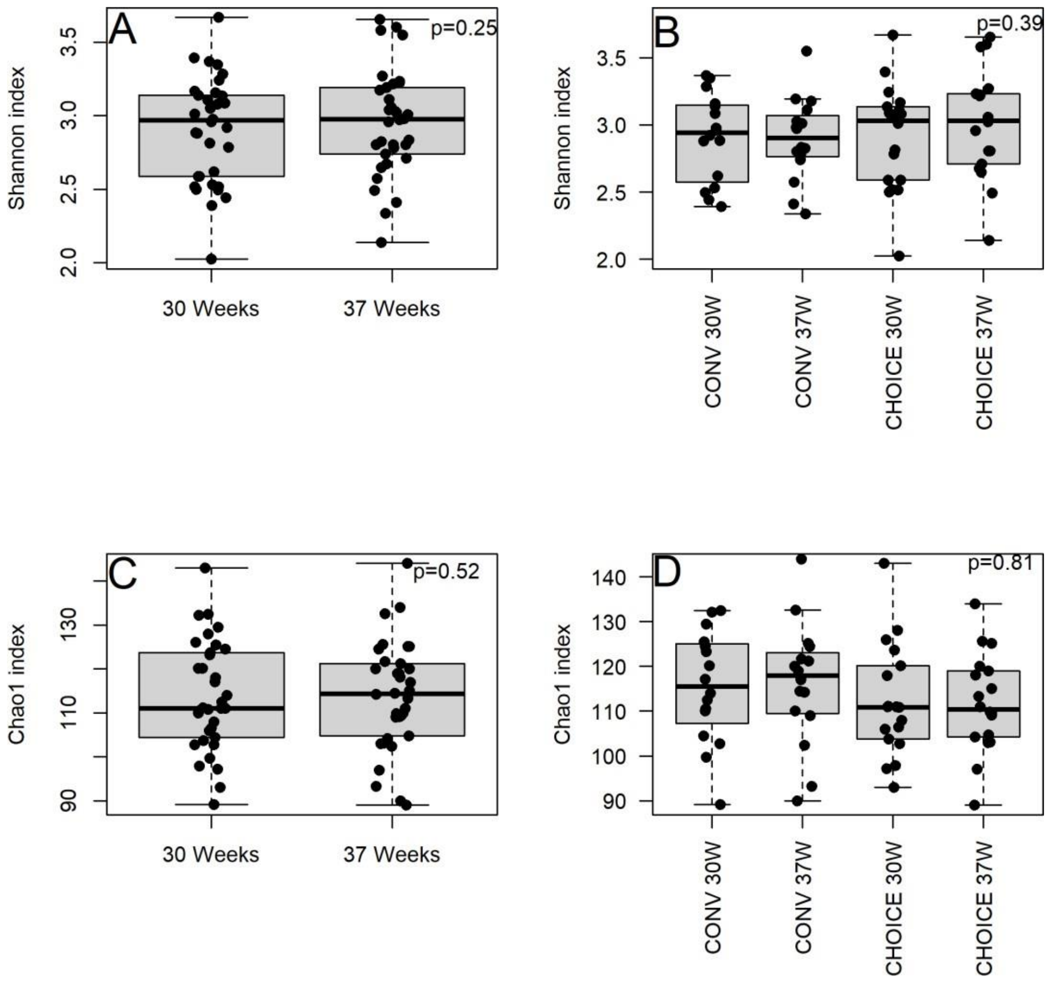

Supplementary Figure 3. Maternal microbiome alpha diversity at the species level. Shannon diversity for weeks of gestation (A) and the interaction between diet group and time (B). Chao1 index of richness, modelled with diet, time, diet/time interaction and all 11 of the maternal measurements, for sample timepoint (C) and the interaction between diet group and time (D).

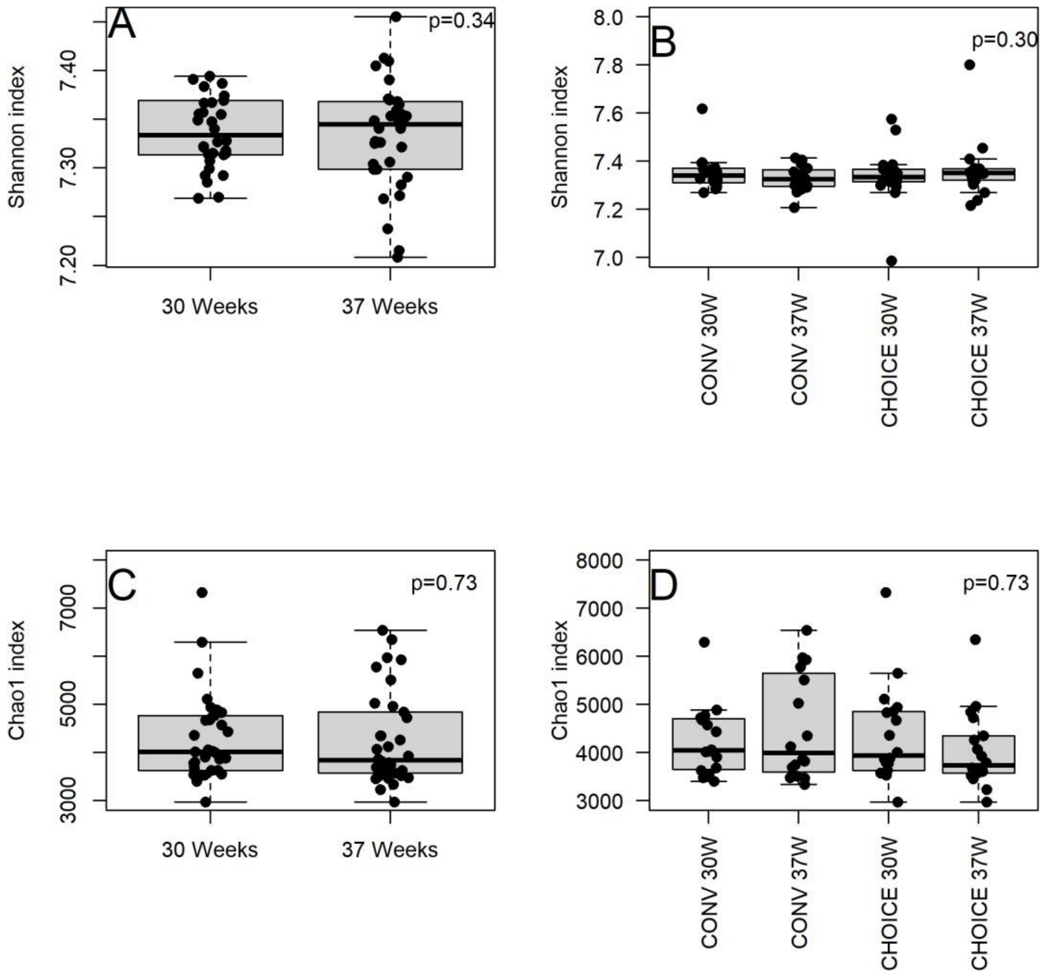

Supplementary Figure 4. Maternal microbiome alpha diversity at the gene annotation level. Shannon diversity for weeks of gestation (A) and the interaction between diet group and time (B). Chao1 index of richness, modelled with diet, time, diet/time interaction and all 11 of the maternal measurements, for sample timepoint (C) and the interaction between diet group and time (D).

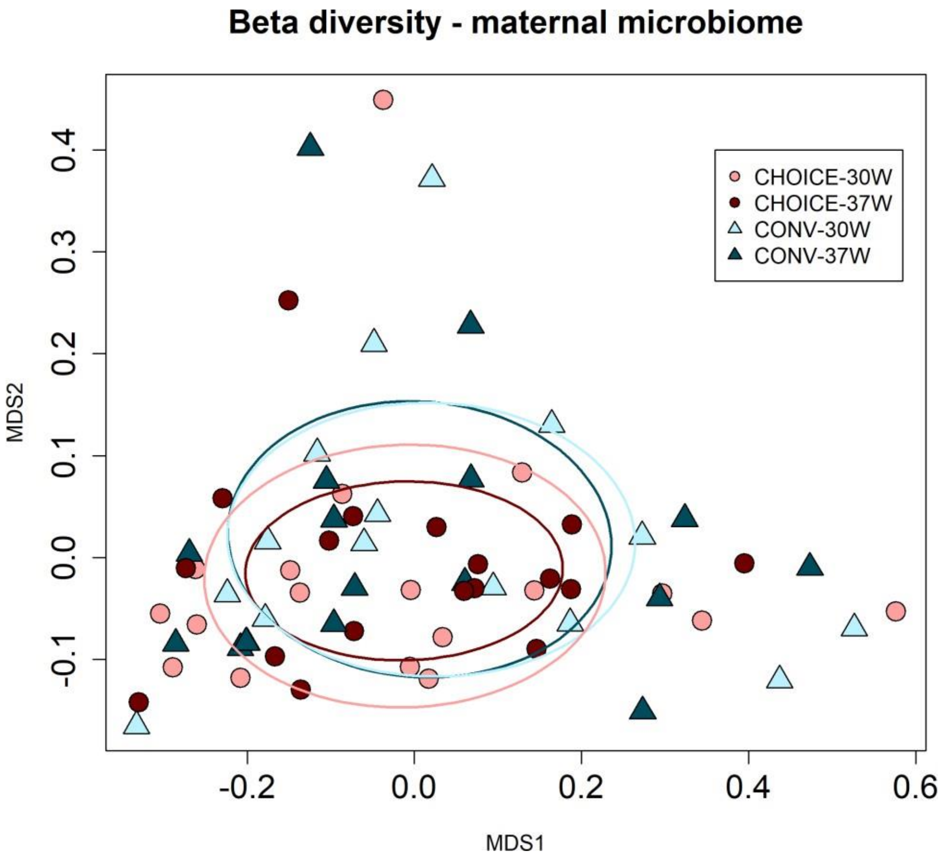

Supplementary Figure 5. Bray-Curtis dissimilarity of the maternal microbiome at the family level ordinated by non-metric multidimensional scaling (NMDS).

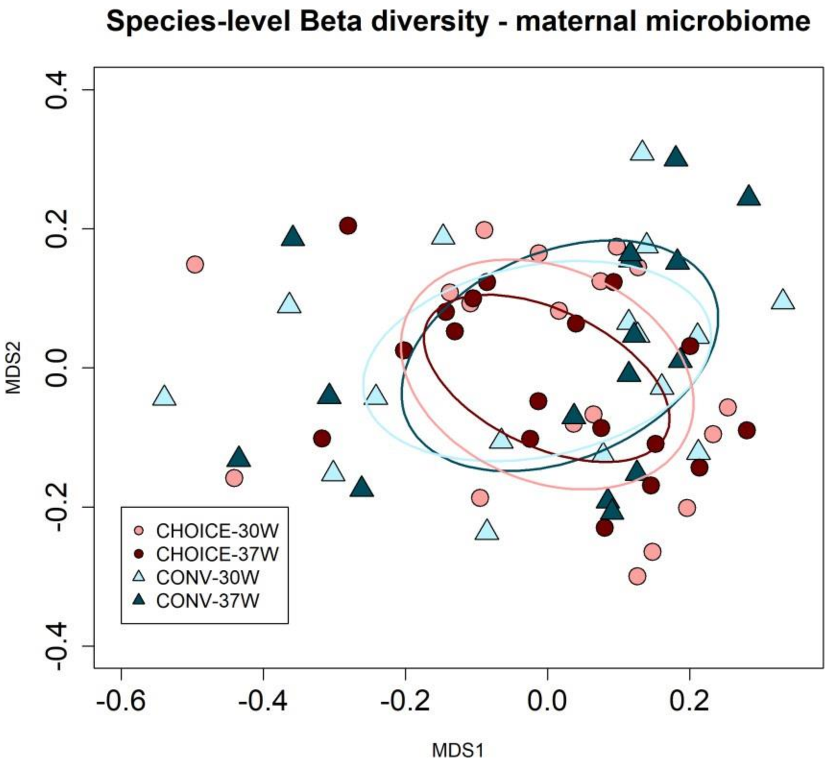

Supplementary Figure 6. Bray-Curtis dissimilarity of the maternal microbiome at the species level ordinated by NMDS.

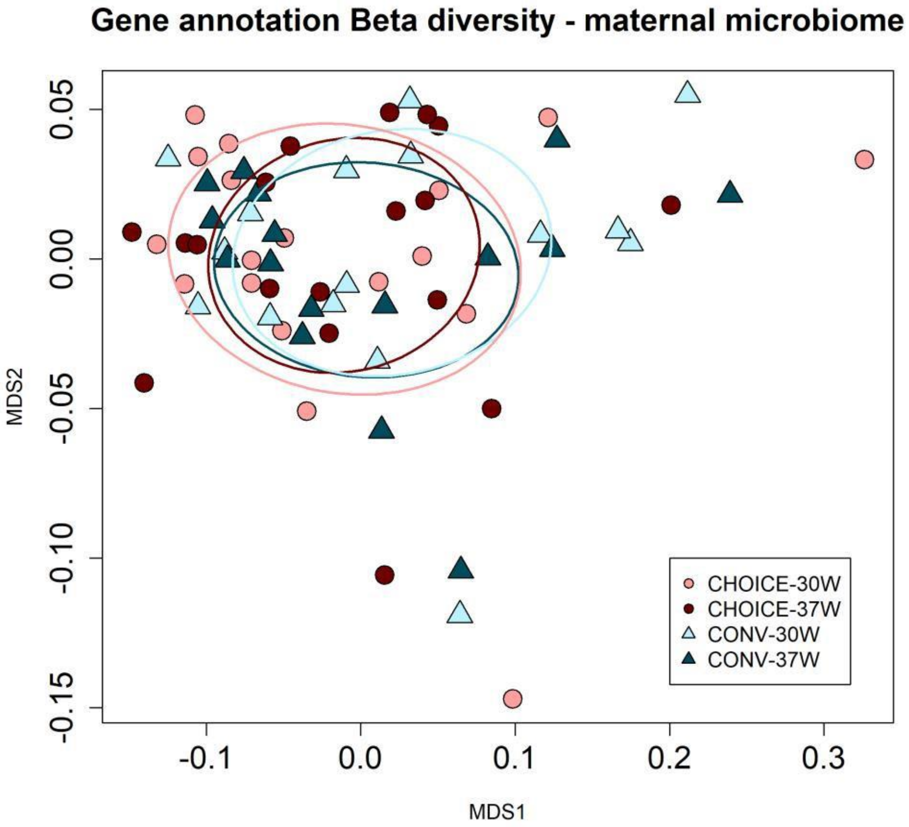

Supplementary Figure 7. Bray-Curtis dissimilarity of the maternal microbiome at the gene annotation level ordinated by NMDS.

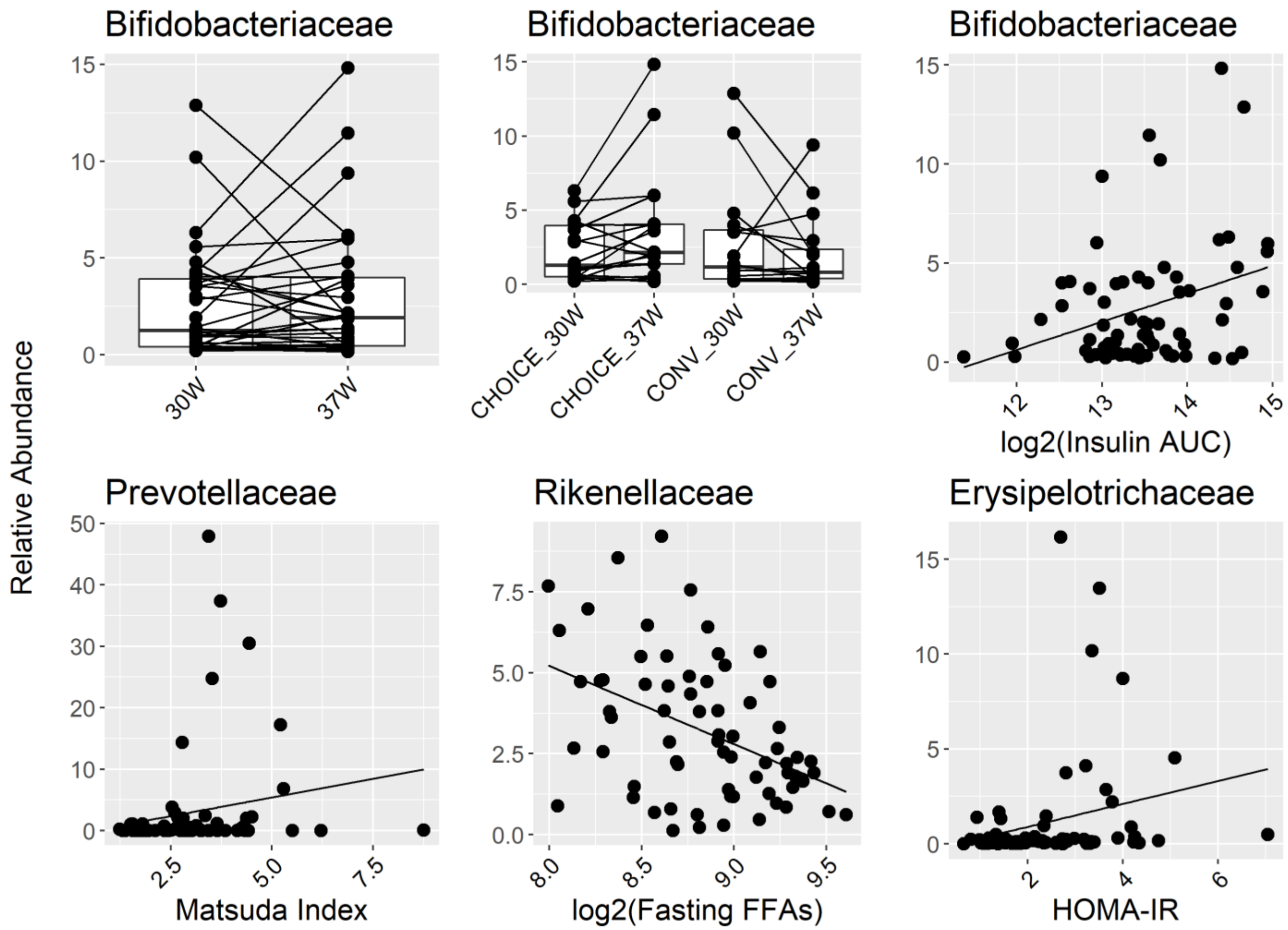

Supplementary Figure 8. Maternal family-level results for the negative binomial regression models.

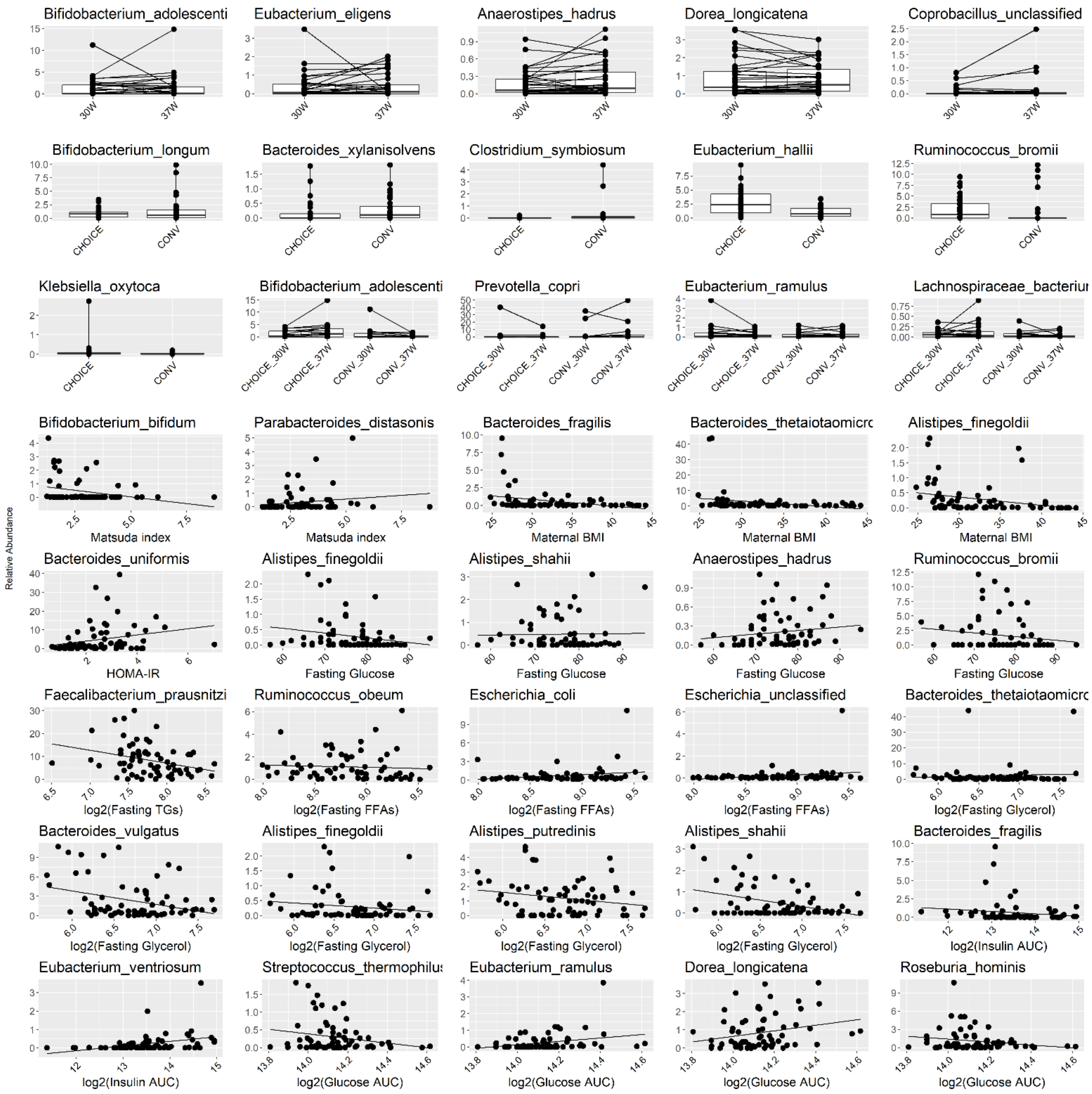

Supplementary Figure 9. Maternal species-level results for the negative binomial regression models.

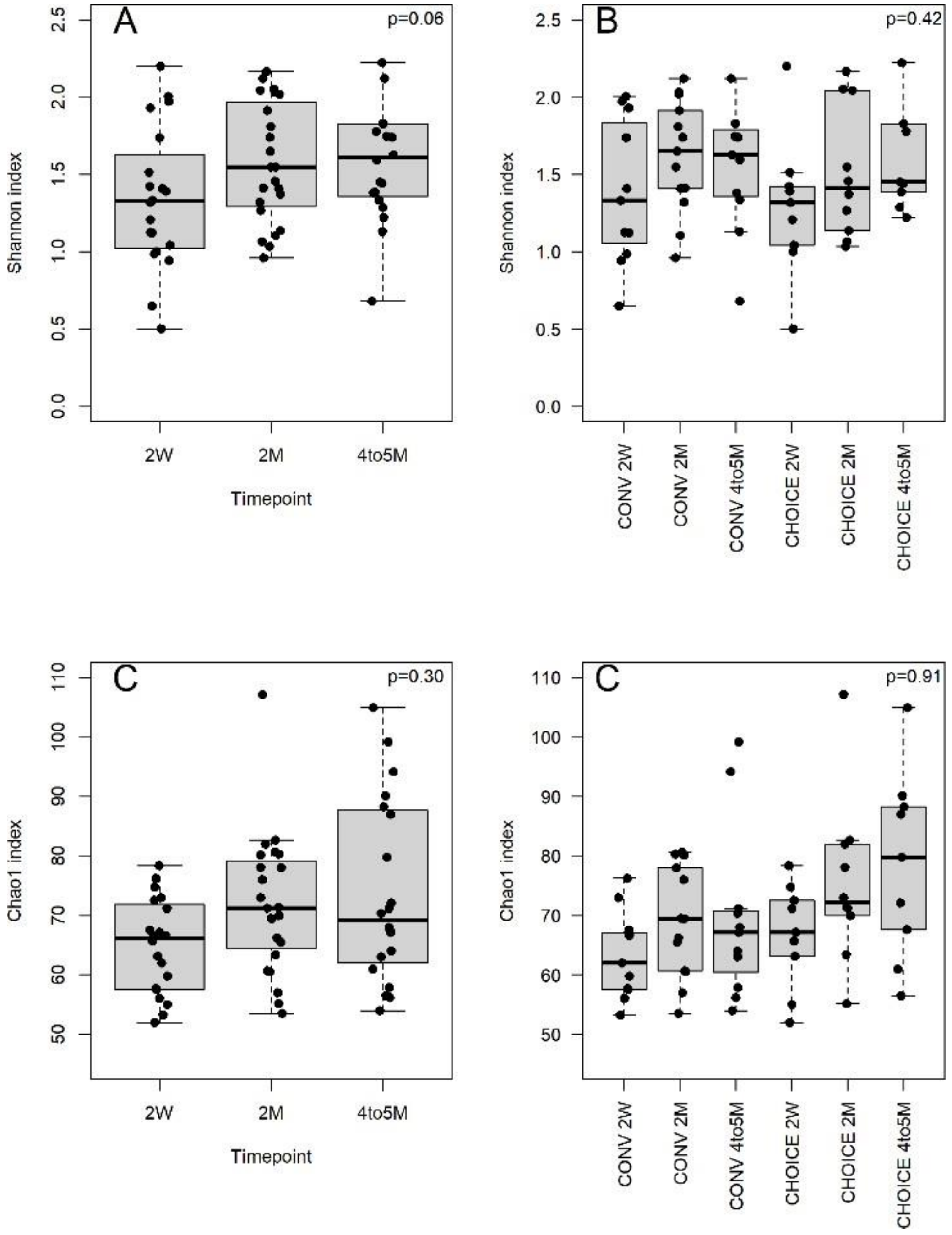

Supplementary Figure 10. Infant microbiome alpha diversity at the species level. Shannon diversity for infant age timepoint (A) and the interaction between diet group and infant age (B). Chao1 index of richness, modelled with diet, infant age, diet/age interaction and all 4 infant variables (maternal gestational weight gain, delivery mode, breastfeeding status, sex), for sample timepoint (C) and the interaction between diet group and infant age (D).

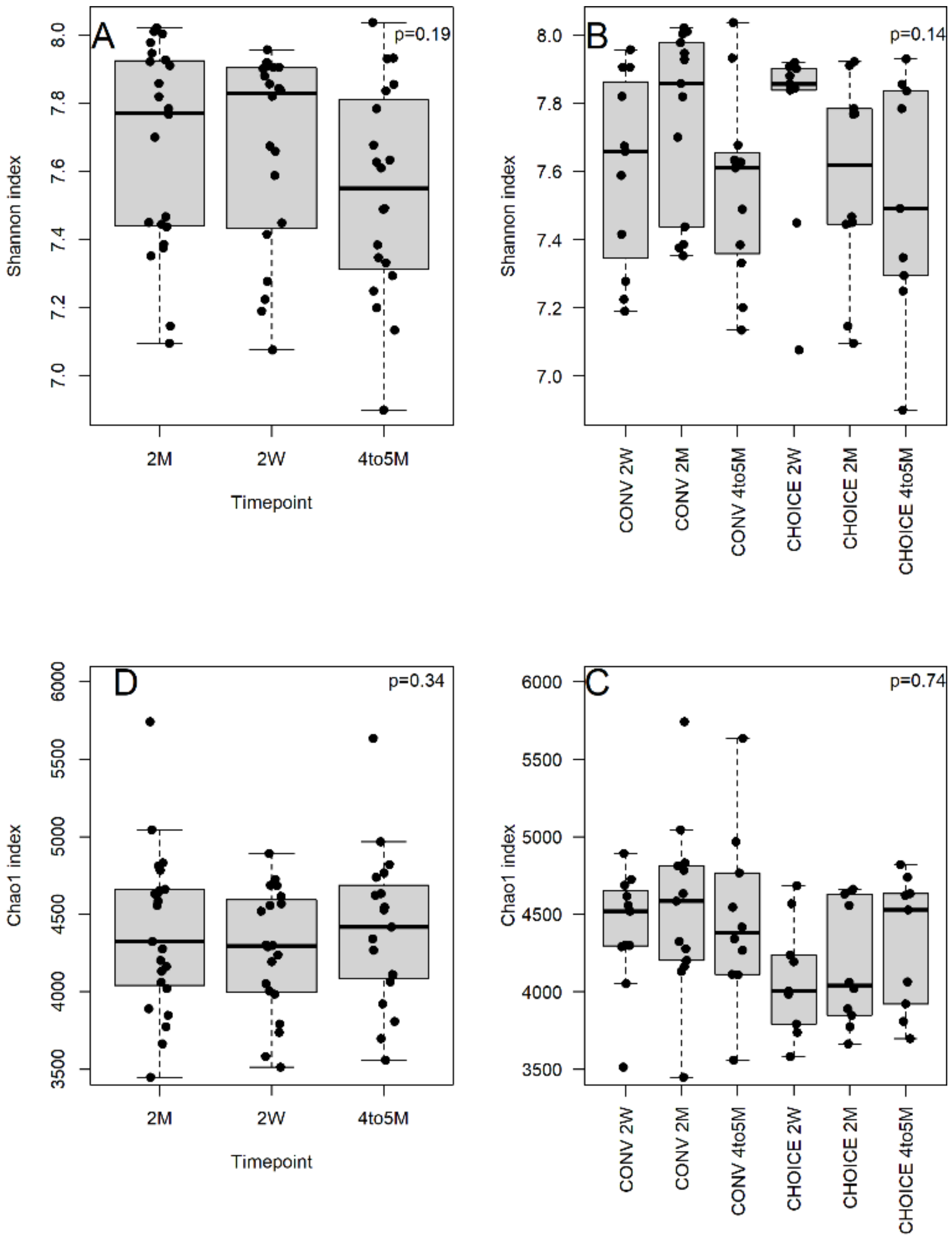

Supplementary Figure 11. Infant microbiome alpha diversity at the gene annotation level. Shannon diversity for infant age timepoint (A) and the interaction between diet group and infant age (B). Chao1 index of richness, modelled with diet, infant age, diet/age interaction and all 4 infant variables (maternal gestational weight gain, delivery mode, breastfeeding status, sex), for sample timepoint (C) the interaction between diet group and infant age (D).

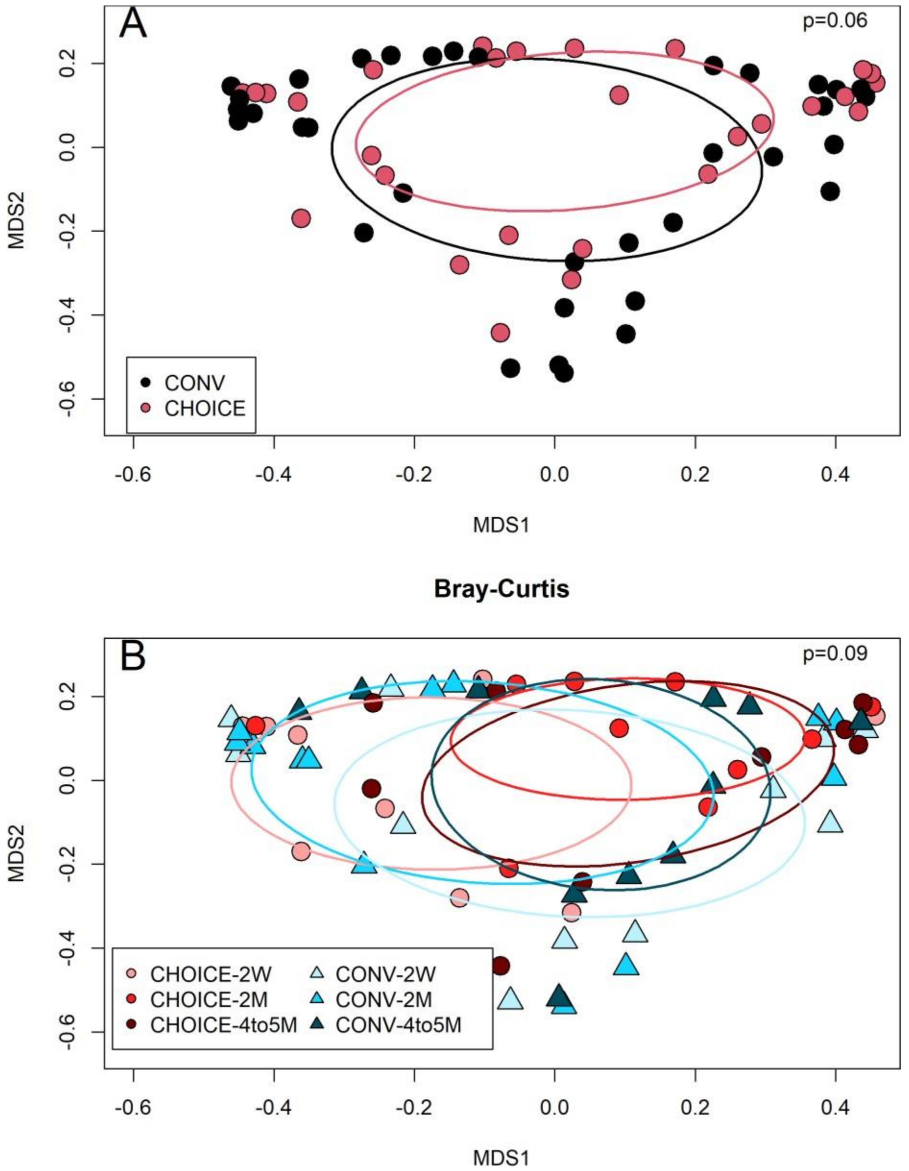

Supplementary Figure 12. Bray-Curtis dissimilarity at the family level ordinated by NMDS. Microbiome community structure between the diet groups (A) and microbial community development within the diet groups over time (B).

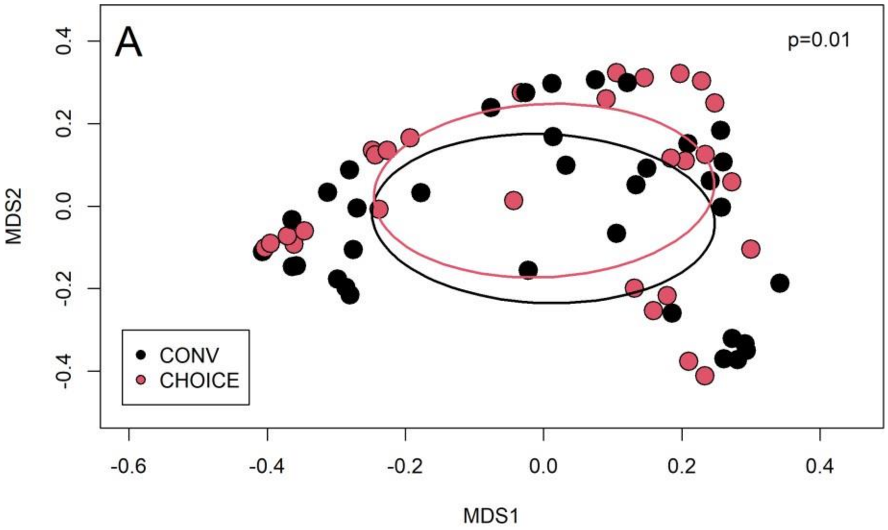

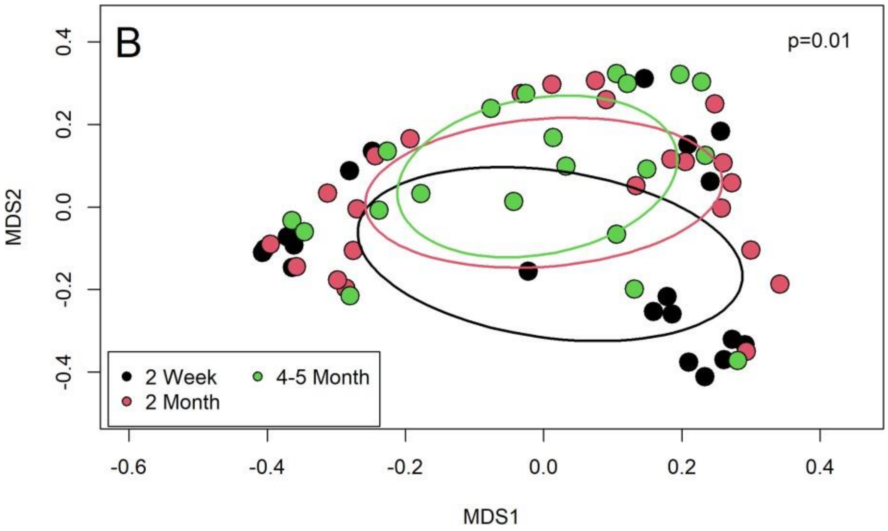

Supplementary Figure 13. Bray-Curtis dissimilarity at the species level ordinated by NMDS. Microbiome community structure between the diet groups (A) and microbial community development within the diet groups over time (B).

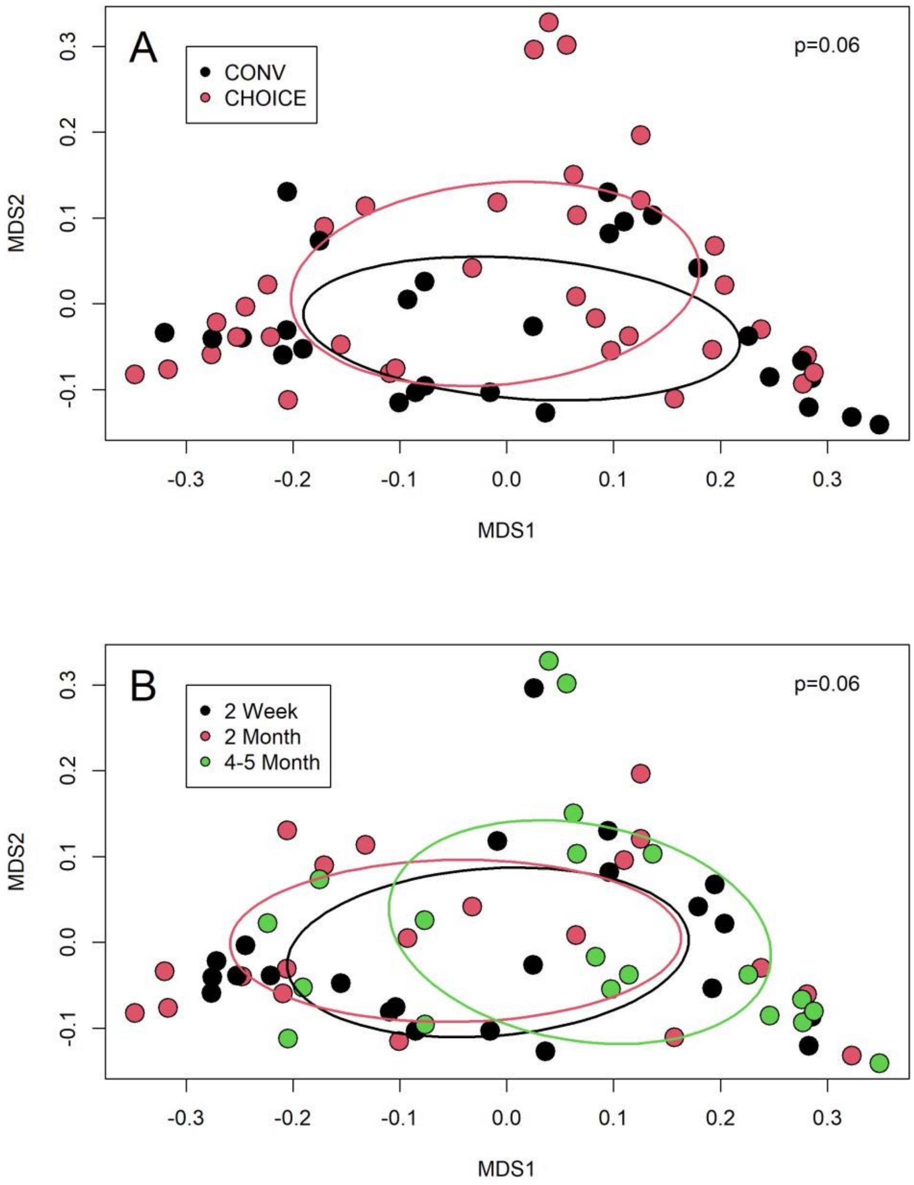

Supplementary Figure 14. Bray-Curtis dissimilarity at the gene annotation level ordinated by NMDS. Microbiome community structure between the diet groups (A) and microbial community development within the diet groups over time (B).

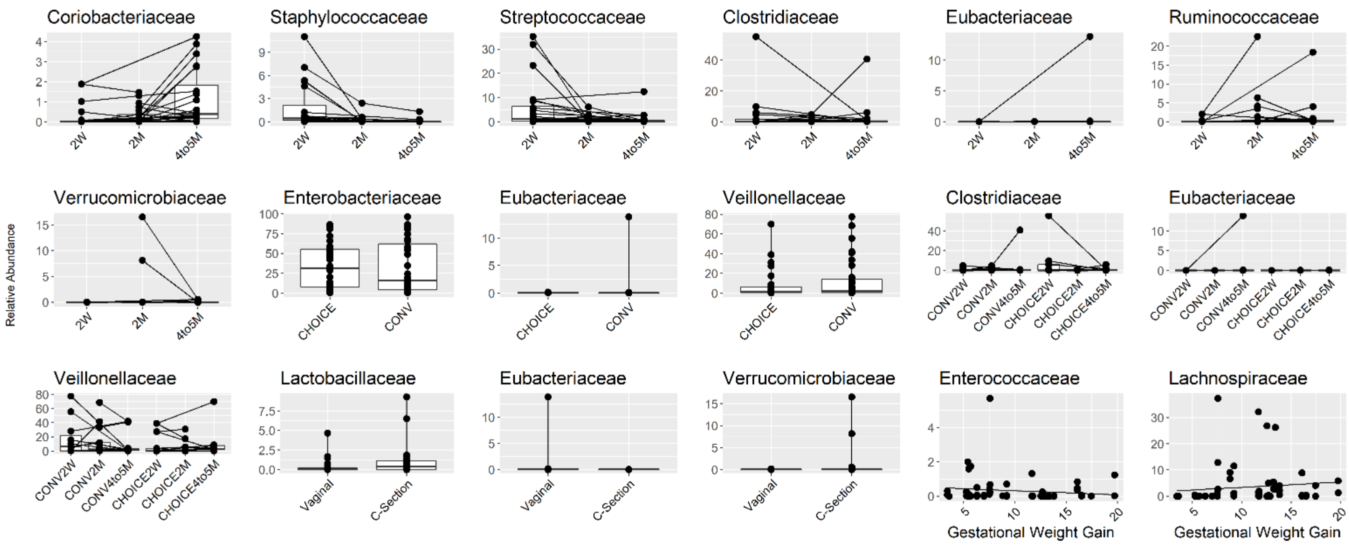

Supplementary Figure 15. Infant family-level results for the negative binomial regression models.

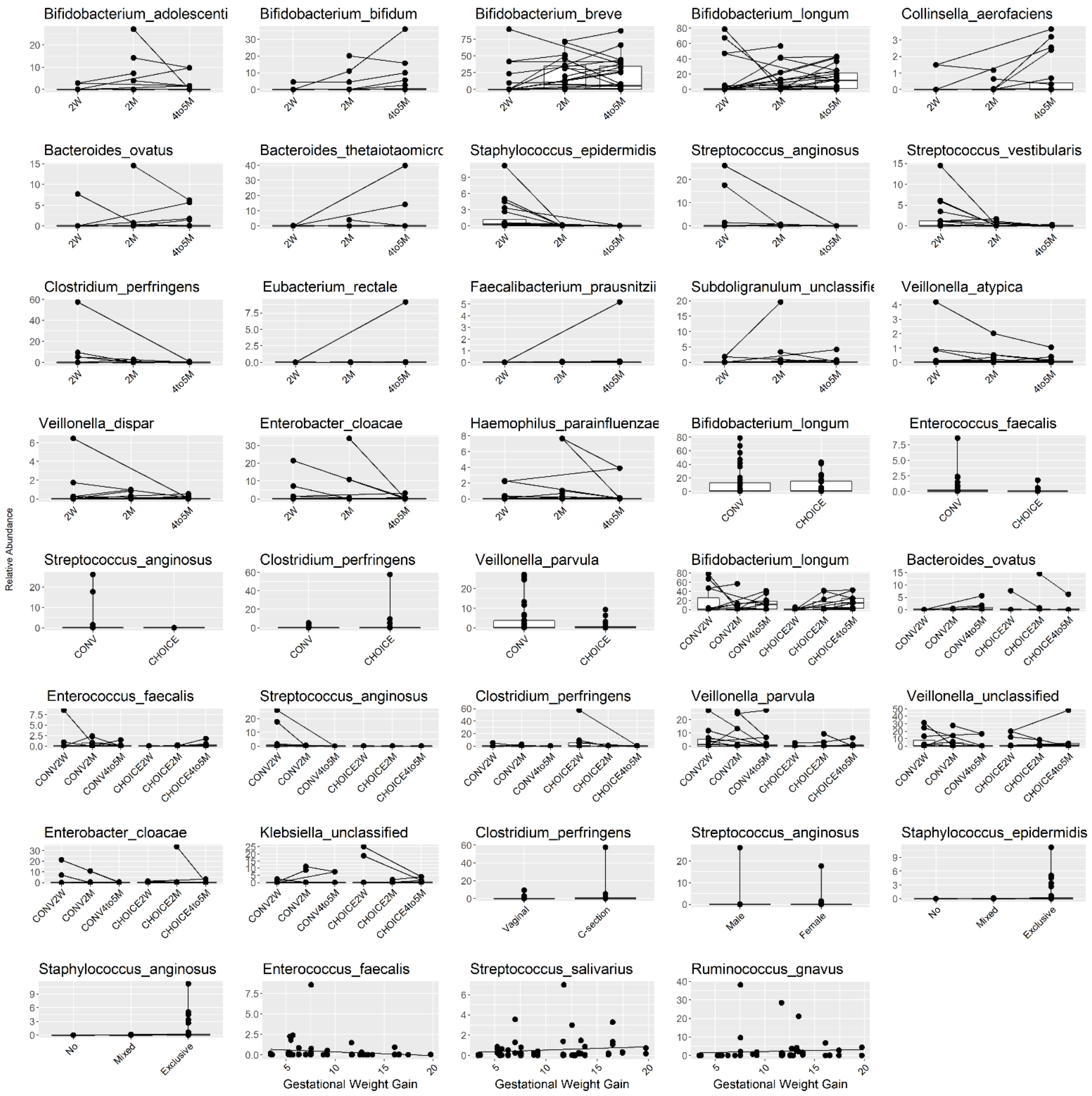

Supplementary Figure 16. Infant species-level results for the negative binomial regression models.

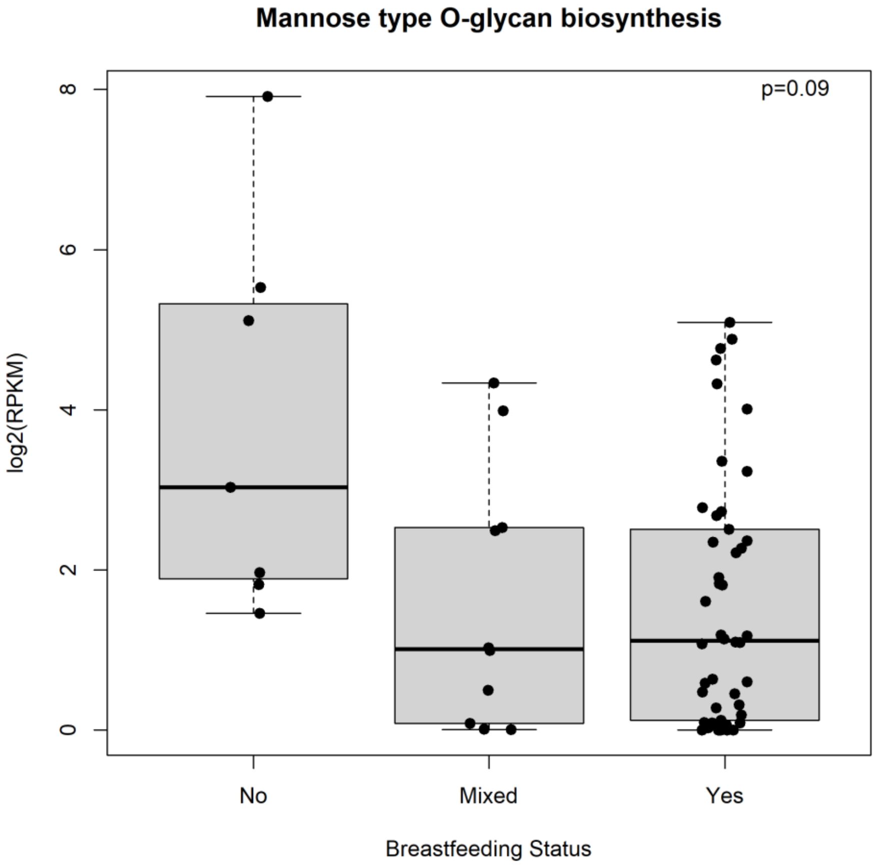

Supplemental Figure 17. Infant gene annotation pathways associated with mannose type O-glycan biosynthesis and infant breastfeeding status.
