# Supplementary material for: A maternal higher-complex carbohydrate diet increases bifidobacteria and alters early life acquisition of the infant microbiome in women with gestational diabetes mellitus
Source: Front Endocrinol (Lausanne). 2022 Jul 28;13:921464. doi: 10.3389/fendo.2022.921464 (PMC9366142; doi:10.3389/fendo.2022.921464)
Supplement: Supplementary file 2 [file Table_1.pdf]

A Maternal Higher-complex Carbohydrate Diet Increases Bifidobacteria and Alters Early Life Acquisition of the Infant Microbiome in Women with Gestational Diabetes Mellitus

Kameron Y. Sugino[1], Teri L. Hernandez[2,3], Linda A. Barbour[2,4], Jennifer M. Kofonow[5], Daniel N. Frank[5], and Jacob E. Friedman[1,6]*

[1]Harold Hamm Diabetes Center, The University of Oklahoma Health Science Center, Oklahoma City, OK, United States; [2]Department of Medicine, Division of Endocrinology, Metabolism and Diabetes, [3]College of Nursing, [4]Department of Obstetrics and Gynecology, and [5]Department of Medicine, Division of Infectious Diseases, The University of Colorado Anschutz Medical Center, Aurora, CO, United States; [5]Department of Pathology, The University of Oklahoma Health Science Center, Oklahoma City, OK, United States

*__Corresponding Author__: Jacob E Friedman, jed-friedman@ouhsc.edu

Supplementary Tables

Supplementary Table 1. Sequencing data and contig length/number after assembly as well as the read coverage after applying our filtering procedures at the family, species, and gene annotation levels.

	Overall Sequencing Output		
	Average Sequencing Depth	Average # of Contigs	Average of the Median Contig Length
Mom	50444099±23059537	242310±82502.7	10334±5321.7
Infant	92172100±45859834	121676±149352.2	16194±43534.3
	Family		
	Total OTUS	OTUs in Analysis	Average Read Coverage (%)
Mom	54	50	98.9
Infant	59	18	98.7
	Species		
	Total OTUS	OTUs in Analysis	Average Read Coverage (%)
Mom	346	67	85.4
Infant	351	42	93.5
	Gene annotation		
	Total Genes	Genes in Analysis	Average Read Coverage (%)
Mom	6236	3664	99.5
Infant	8407	4560	98.9

Supplementary Table 2. Maternal family-level results for the negative binomial regression models.

	comparison	chisq	pval	adjpval
Bifidobacteriaceae				
	Insulin AUC	6.440023	0.011158	0.194634
	Diet Group	0.051936	0.819728	0.99735
	Sample timepoint	4.677469	0.030561	0.341262
	Diet Group:Sample timepoint	8.454525	0.003641	0.121986
Coriobacteriaceae				
	Diet Group	0.376236	0.539625	0.99735
	Sample timepoint	0.027236	0.868918	0.99735
	Diet Group:Sample timepoint	0.0801	0.777162	0.99735
Bacteroidaceae				
	Diet Group	0.222941	0.636808	0.99735
	Sample timepoint	0.026942	0.869622	0.99735
	Diet Group:Sample timepoint	0.006381	0.93633	0.99735
Porphyromonadaceae				
	Diet Group	0.897271	0.343514	0.99735
	Sample timepoint	1.041131	0.307559	0.99735
	Diet Group:Sample timepoint	0.795871	0.372331	0.99735
Prevotellaceae				
	Matsuda Index	6.367957	0.01162	0.194634
	Diet Group	0.007967	0.928876	0.99735
	Sample timepoint	0.108156	0.742254	0.99735
	Diet Group:Sample timepoint	0.078969	0.7787	0.99735
Rikenellaceae				
	Fasting FFAs	13.24997	0.000273	0.018263
	Diet Group	0.196099	0.657888	0.99735
	Sample timepoint	0.222324	0.637275	0.99735
	Diet Group:Sample timepoint	0.032159	0.85768	0.99735
Streptococcaceae				
	Diet Group	0.442662	0.50584	0.99735
	Sample timepoint	0.094905	0.758032	0.99735
	Diet Group:Sample timepoint	0.91471	0.338868	0.99735
Clostridiaceae				
	Diet Group	1.294835	0.255159	0.99735
	Sample timepoint	0.087414	0.767491	0.99735

	Diet Group:Sample timepoint	0.721233	0.39574	0.99735
Clostridiales_noname				
	Diet Group	0.058185	0.809388	0.99735
	Sample timepoint	0.545206	0.460283	0.99735
	Diet Group:Sample timepoint	0.001222	0.972109	0.99735
Eubacteriaceae				
	Diet Group	0.118149	0.73105	0.99735
	Sample timepoint	0.459306	0.497949	0.99735
	Diet Group:Sample timepoint	0.000268	0.986949	0.99735
Lachnospiraceae				
	Diet Group	0.611693	0.434151	0.99735
	Sample timepoint	0.148339	0.700127	0.99735
	Diet Group:Sample timepoint	0.383514	0.535728	0.99735
Oscillospiraceae				
	Diet Group	1.255661	0.262474	0.99735
	Sample timepoint	0.09567	0.75709	0.99735
	Diet Group:Sample timepoint	0.000896	0.976121	0.99735
Peptostreptococcaceae				
	Diet Group	0.002935	0.956794	0.99735
	Sample timepoint	0.061678	0.803863	0.99735
	Diet Group:Sample timepoint	0.105135	0.745752	0.99735
Ruminococcaceae				
	Diet Group	2.598449	0.106968	0.89586
	Sample timepoint	0.084351	0.771486	0.99735
	Diet Group:Sample timepoint	0.206018	0.649906	0.99735
Erysipelotrichaceae				
	HOMA_IR	5.600667	0.017954	0.240579
	Diet Group	0.015168	0.901983	0.99735
	Sample timepoint	1.562562	0.21129	0.99735
	Diet Group:Sample timepoint	3.347116	0.067323	0.644376
Veillonellaceae				
	Diet Group	0.024089	0.876658	0.99735
	Sample timepoint	1.10E-05	0.99735	0.99735
	Diet Group:Sample timepoint	0.000125	0.991062	0.99735
Sutterellaceae				

	Diet Group	0.531191	0.466107	0.99735
	Sample timepoint	0.406655	0.523672	0.99735
	Diet Group:Sample timepoint	0.000742	0.978262	0.99735
Desulfovibrionaceae				
	Diet Group	1.183225	0.276701	0.99735
	Sample timepoint	0.148576	0.6999	0.99735
	Diet Group:Sample timepoint	0.479578	0.488614	0.99735
Enterobacteriaceae				
	HOMA_IR	0.068046	0.794203	0.99735
	Fasting Insulin	0.100163	0.751634	0.99735
	Fasting Triglycerides	2.22984	0.135368	0.99735
	Diet Group	1.567313	0.210598	0.99735
	Sample timepoint	0.17017	0.679961	0.99735
	Diet Group:Sample timepoint	0.145055	0.703306	0.99735
Verrucomicrobiaceae				
	Diet Group	0.031936	0.858168	0.99735
	Sample timepoint	1.710071	0.190976	0.99735
	Diet Group:Sample timepoint	0.591474	0.44185	0.99735

Supplementary Table 3. Maternal species-level results for the negative binomial regression models.

	comparison	chisq	pval	adjpval
Bifidobacterium_adolescentis				
	Diet Group	1.161042	0.28125	0.721076
	Sample Timepoint	6.440964	0.011152	0.115986
	Diet Group:Sample Timepoint	11.55073	0.000677	0.023316
Bifidobacterium_bifidum				
	Matsuda Index	6.378593	0.01155	0.115986
	Diet Group	0.050633	0.821966	0.984631
	Sample Timepoint	0.19527	0.658566	0.940003
	Diet Group:Sample Timepoint	0.687589	0.406986	0.883325
Bifidobacterium_breve				
	Diet Group	1.322443	0.250154	0.706072
	Sample Timepoint	0.002852	0.957407	0.990398
	Diet Group:Sample Timepoint	0.000693	0.978995	0.995518
Bifidobacterium_longum				
	Diet Group	0.440992	0.506644	0.920866
	Sample Timepoint	4.702479	0.030119	0.186583
	Diet Group:Sample Timepoint	3.083379	0.079096	0.36658
Bifidobacterium_pseudocatenulatum				
	Diet Group	0.022637	0.880404	0.984631
	Sample Timepoint	1.677571	0.195248	0.67221
	Diet Group:Sample Timepoint	2.409361	0.120612	0.509954
Collinsella_aerofaciens				
	Diet Group	1.779597	0.182199	0.666803
	Sample Timepoint	0.302208	0.582502	0.940003
	Diet Group:Sample Timepoint	0.776622	0.378176	0.838693

*Eggerthella*_unclassified				
	Diet Group	0.0593	0.807606	0.984631
	Sample Timepoint	0.045244	0.831555	0.984631
	Diet Group:Sample Timepoint	0.201072	0.653857	0.940003
Bacteroides_caccae				
	Diet Group	0.001527	0.968827	0.990398
	Sample Timepoint	0.553166	0.457027	0.917231
	Diet Group:Sample Timepoint	0.287061	0.59211	0.940003
Bacteroides_dorei				
	Diet Group	0.011032	0.916348	0.984631
	Sample Timepoint	0.783737	0.376001	0.838693
	Diet Group:Sample Timepoint	0.487181	0.485188	0.917231
Bacteroides_fragilis				
	Diet Group	1.847244	0.174104	0.655611
	Sample Timepoint	2.816217	0.093316	0.424324
	Insulin AUC	6.601631	0.010189	0.111611
	Maternal BMI	4.173633	0.041058	0.220717
	Diet Group:Sample Timepoint	0.517385	0.471959	0.917231
Bacteroides_ovatus				
	Diet Group	0.010082	0.920021	0.984631
	Sample Timepoint	0.19247	0.660868	0.940003
	Diet Group:Sample Timepoint	0.490451	0.483726	0.917231
Bacteroides_stercoris				
	Diet Group	1.333866	0.248119	0.706072
	Sample Timepoint	0.101681	0.749822	0.957235
	Diet Group:Sample Timepoint	0.103833	0.747278	0.957235
Bacteroides_thetaiotaomicron				

	Diet Group	2.55E-06	0.998727	0.998727
	Sample Timepoint	2.009615	0.156305	0.617533
	Fasting Glycerol	6.28064	0.012206	0.11767
	Maternal BMI	4.654726	0.030968	0.186583
	Diet Group:Sample Timepoint	0.033462	0.854856	0.984631
Bacteroides_uniformis				
	HOMA-IR	8.244121	0.004088	0.068583
	Diet Group	0.013143	0.908729	0.984631
	Sample Timepoint	0.233145	0.629202	0.940003
	Diet Group:Sample Timepoint	0.188512	0.664158	0.940003
Bacteroides_vulgatus				
	Fasting Glycerol	12.76068	0.000354	0.014218
	Diet Group	1.541448	0.214402	0.706072
	Sample Timepoint	1.750224	0.185849	0.666803
	Diet Group:Sample Timepoint	0.007226	0.932258	0.984631
Bacteroides_xylanisolvens				
	Diet Group	4.70367	0.030098	0.186583
	Sample Timepoint	0.635948	0.425182	0.886143
	Diet Group:Sample Timepoint	0.239387	0.624649	0.940003
Parabacteroides_distasonis				
	Matsuda Index	7.143197	0.007525	0.086357
	Diet Group	0.229313	0.632033	0.940003
	Sample Timepoint	0.44877	0.50292	0.920866
	Diet Group:Sample Timepoint	0.189036	0.66372	0.940003
Parabacteroides_merdae				
	Diet Group	0.223778	0.636176	0.940003
	Sample Timepoint	0.377826	0.538769	0.934125

	Diet Group:Sample Timepoint	0.380272	0.537457	0.934125
Prevotella_copri				
	Diet Group	0.179872	0.671484	0.940003
	Sample Timepoint	0.953288	0.328884	0.808786
	Diet Group:Sample Timepoint	7.706719	0.005502	0.07366
Alistipes_finegoldii				
	Diet Group	0.161877	0.687435	0.946696
	Sample Timepoint	1.440475	0.230062	0.706072
	Fasting Glycerol	15.10756	0.000102	0.008158
	Fasting Glucose	9.839424	0.001708	0.041165
	Maternal BMI	5.298282	0.021346	0.147922
	Diet Group:Sample Timepoint	1.260718	0.261515	0.706436
Alistipes_onderdonkii				
	Diet Group	0.356253	0.550595	0.938577
	Sample Timepoint	0.104278	0.746755	0.957235
	Diet Group:Sample Timepoint	0.103148	0.748084	0.957235
Alistipes_putredinis				
	Fasting Glycerol	12.91673	0.000326	0.014218
	Diet Group	0.260642	0.609679	0.940003
	Sample Timepoint	0.011442	0.914816	0.984631
	Diet Group:Sample Timepoint	1.232485	0.266924	0.706436
Alistipes_shahii				
	Diet Group	0.02394	0.877038	0.984631
	Sample Timepoint	1.067695	0.301467	0.756807
	Fasting FFAs	0.656387	0.417838	0.883325
	Fasting Glycerol	4.660622	0.030862	0.186583
	Fasting Triglycerides	0.097652	0.754666	0.957235
	Fasting Glucose	11.01521	0.000904	0.027223
	Maternal BMI	2.651038	0.103482	0.461838

	Diet Group:Sample Timepoint	1.481886	0.22348	0.706072
Streptococcus_salivarius				
	Diet Group	0.03974	0.841991	0.984631
	Sample Timepoint	1.769852	0.1834	0.666803
	Diet Group:Sample Timepoint	1.274551	0.258915	0.706436
Streptococcus_thermophilus				
	Glucose AUC	7.961234	0.004779	0.068583
	Diet Group	0.026255	0.87128	0.984631
	Sample Timepoint	0.772886	0.379326	0.838693
	Diet Group:Sample Timepoint	0.000265	0.987021	0.996846
Clostridium_bolteae				
	Diet Group	0.283521	0.594403	0.940003
	Sample Timepoint	1.218389	0.269677	0.706436
	Diet Group:Sample Timepoint	3.318943	0.068486	0.336839
Clostridium_leptum				
	Diet Group	1.414092	0.234378	0.706072
	Sample Timepoint	0.050495	0.822205	0.984631
	Diet Group:Sample Timepoint	0.11015	0.739974	0.957235
Clostridium_symbiosum				
	Diet Group	4.825441	0.028043	0.186583
	Sample Timepoint	0.10171	0.749787	0.957235
	Diet Group:Sample Timepoint	0.339199	0.560292	0.938577
Flavonifractor_plautii				
	Diet Group	0.001429	0.96985	0.990398
	Sample Timepoint	1.49707	0.221123	0.706072
	Diet Group:Sample Timepoint	0.108411	0.74196	0.957235

Eubacterium_eligens				
	Diet Group	1.580019	0.208758	0.699454
	Sample Timepoint	4.206029	0.040281	0.220717
	Diet Group:Sample Timepoint	1.440444	0.230068	0.706072
Eubacterium_hallii				
	Diet Group	7.489897	0.006205	0.077531
	Sample Timepoint	0.217474	0.640971	0.940003
	Diet Group:Sample Timepoint	0.656595	0.417765	0.883325
Eubacterium_ramulus				
	Glucose AUC	25.22228	5.11E-07	0.000123
	Diet Group	0.407032	0.52348	0.920866
	Sample Timepoint	1.327701	0.249215	0.706072
	Diet Group:Sample Timepoint	5.652162	0.017434	0.140052
Eubacterium_rectale				
	Diet Group	0.075839	0.783017	0.978663
	Sample Timepoint	0.010625	0.917902	0.984631
	Diet Group:Sample Timepoint	0.132969	0.715373	0.957235
Eubacterium_siraeum				
	Diet Group	3.529162	0.060298	0.309189
	Sample Timepoint	0.043819	0.834191	0.984631
	Diet Group:Sample Timepoint	0.183751	0.668169	0.940003
Eubacterium_sp_3_1_31				
	Diet Group	0.261753	0.608918	0.940003
	Sample Timepoint	1.312407	0.251959	0.706072
	Diet Group:Sample Timepoint	3.241613	0.07179	0.339243
Eubacterium_ventriosum				
	Insulin AUC	5.966692	0.014579	0.130128

	Diet Group	1.246916	0.264142	0.706436
	Sample Timepoint	0.00528	0.942073	0.987128
	Diet Group:Sample Timepoint	0.173769	0.676784	0.940003
Anaerostipes_hadrus				
	Fasting Glucose	5.357727	0.020631	0.147922
	Diet Group	0.981166	0.321911	0.7998
	Sample Timepoint	3.967933	0.046375	0.242963
	Diet Group:Sample Timepoint	0.418764	0.517554	0.920866
Ruminococcus_gnavus				
	Diet Group	0.05113	0.821109	0.984631
	Sample Timepoint	0.006528	0.935604	0.984631
	Diet Group:Sample Timepoint	0.006642	0.935044	0.984631
Ruminococcus_obeum				
	Fasting FFAs	5.89691	0.015167	0.130549
	Diet Group	1.508549	0.219361	0.706072
	Sample Timepoint	1.173326	0.278719	0.721076
	Diet Group:Sample Timepoint	1.710577	0.190911	0.666803
Ruminococcus_torques				
	Diet Group	0.866461	0.351937	0.838693
	Sample Timepoint	0.028333	0.866329	0.984631
	Diet Group:Sample Timepoint	0.334231	0.563178	0.938577
Coprococcus_catus				
	Diet Group	0.012886	0.909623	0.984631
	Sample Timepoint	1.410647	0.234949	0.706072
	Diet Group:Sample Timepoint	0.55565	0.456018	0.917231
Coprococcus_comes				
	Diet Group	1.864786	0.172073	0.655611

	Sample Timepoint	0.171622	0.678675	0.940003
	Diet Group:Sample Timepoint	0.410876	0.521525	0.920866
Dorea_formicigenerans				
	Diet Group	1.578578	0.208966	0.699454
	Sample Timepoint	0.331625	0.564704	0.938577
	Diet Group:Sample Timepoint	0.773723	0.379068	0.838693
Dorea_longicatena				
	Glucose AUC	8.69989	0.003182	0.063847
	Diet Group	0.037064	0.847334	0.984631
	Sample Timepoint	5.31157	0.021184	0.147922
	Diet Group:Sample Timepoint	3.2676	0.070661	0.339243
Lachnospiraceae_bacterium_1_4_56FAA				
	Diet Group	0.505702	0.477005	0.917231
	Sample Timepoint	0.09077	0.7632	0.962991
	Diet Group:Sample Timepoint	0.020882	0.885101	0.984631
Lachnospiraceae_bacterium_3_1_46FAA				
	Diet Group	1.337867	0.24741	0.706072
	Sample Timepoint	0.069638	0.791864	0.978663
	Diet Group:Sample Timepoint	1.070549	0.300822	0.756807
Lachnospiraceae_bacterium_3_1_57FAA_CT1				
	Diet Group	0.037968	0.845507	0.984631
	Sample Timepoint	0.148853	0.699634	0.957235
	Diet Group:Sample Timepoint	4.167248	0.041213	0.220717
Lachnospiraceae_bacterium_5_1_63FAA				
	Diet Group	0.79478	0.372659	0.838693
	Sample Timepoint	0.411836	0.521039	0.920866

	Diet Group:Sample Timepoint	0.037683	0.846081	0.984631
Lachnospiraceae_bacterium_7_1_58FAA				
	Diet Group	0.340302	0.559655	0.938577
	Sample Timepoint	2.373553	0.123406	0.512773
	Diet Group:Sample Timepoint	0.174349	0.676275	0.940003
Roseburia_hominis				
	Glucose AUC	8.555897	0.003444	0.063847
	Diet Group	0.069952	0.791407	0.978663
	Sample Timepoint	0.512098	0.474232	0.917231
	Diet Group:Sample Timepoint	0.102379	0.748993	0.957235
Roseburia_intestinalis				
	Fasting Insulin	0.785812	0.37537	0.838693
	Diet Group	0.002702	0.958547	0.990398
	Sample Timepoint	0.219783	0.639205	0.940003
	Diet Group:Sample Timepoint	0.000205	0.988573	0.996846
Roseburia_inulinivorans				
	Diet Group	0.004182	0.94844	0.989498
	Sample Timepoint	0.237712	0.625864	0.940003
	Diet Group:Sample Timepoint	0.032036	0.857948	0.984631
Oscillibacter_unclassified				
	Diet Group	1.277079	0.258443	0.706436
	Sample Timepoint	0.131712	0.716664	0.957235
	Diet Group:Sample Timepoint	4.37E-06	0.998333	0.998727
Clostridium_bartlettii				
	Diet Group	0.002246	0.962203	0.990398
	Sample Timepoint	0.303672	0.581589	0.940003

	Diet Group:Sample Timepoint	0.029249	0.864206	0.984631
Faecalibacterium_prausnitzii				
	Fasting Triglycerides	6.126352	0.013318	0.123449
	Diet Group	0.840789	0.359171	0.838693
	Sample Timepoint	2.228914	0.135449	0.553275
	Diet Group:Sample Timepoint	0.732569	0.392052	0.85895
Ruminococcus_bromii				
	Fasting Glucose	7.939088	0.004838	0.068583
	Diet Group	5.287219	0.021483	0.147922
	Sample Timepoint	0.006808	0.934242	0.984631
	Diet Group:Sample Timepoint	0.282404	0.59513	0.940003
Ruminococcus_sp_5_1_39BFAA				
	Diet Group	0.662607	0.415641	0.883325
	Sample Timepoint	0.100907	0.750744	0.957235
	Diet Group:Sample Timepoint	0.106735	0.743893	0.957235
*Subdoligranulum*_unclassified				
	Diet Group	0.43144	0.511283	0.920866
	Sample Timepoint	0.251995	0.615674	0.940003
	Diet Group:Sample Timepoint	0.622278	0.430202	0.886143
*Coprobacillus*_unclassified				
	Diet Group	0.009129	0.923883	0.984631
	Sample Timepoint	4.19666	0.040504	0.220717
	Diet Group:Sample Timepoint	2.422759	0.119584	0.509954
Holdemania_filiformis				
	Diet Group	1.328289	0.24911	0.706072
	Sample Timepoint	0.278416	0.597741	0.940003

	Diet Group:Sample Timepoint	0.071103	0.789738	0.978663
*Bilophila*_unclassified				
	Diet Group	0.775563	0.378502	0.838693
	Sample Timepoint	0.545959	0.459973	0.917231
	Diet Group:Sample Timepoint	0.470258	0.492868	0.920785
Citrobacter_freundii				
	Diet Group	0.816812	0.366114	0.838693
	Sample Timepoint	0.283823	0.594206	0.940003
	Diet Group:Sample Timepoint	0.174539	0.676109	0.940003
Escherichia_coli				
	Fasting FFAs	5.749894	0.01649	0.137035
	Diet Group	0.528154	0.467384	0.917231
	Sample Timepoint	2.066101	0.150606	0.604935
	Diet Group:Sample Timepoint	0.543953	0.460799	0.917231
*Escherichia*_unclassified				
	Fasting FFAs	7.424521	0.006434	0.077531
	Diet Group	0.059049	0.808005	0.984631
	Sample Timepoint	1.915616	0.166341	0.646583
	Diet Group:Sample Timepoint	0.047862	0.826826	0.984631
Klebsiella_oxytoca				
	Diet Group	4.46608	0.034574	0.203229
	Sample Timepoint	0.45058	0.50206	0.920866
	Diet Group:Sample Timepoint	0.906295	0.341099	0.830353
Akkermansia_muciniphila				
	Diet Group	0.029177	0.864371	0.984631
	Sample Timepoint	1.726348	0.188878	0.666803

	Diet Group:Sample Timepoint	0.628513	0.427901	0.886143

Supplementary Table 4. Maternal protein gene annotation results.

	comparison	chisq	pval	adjpval
ABC transporters				
	Diet Group	1.59476	0.206647	0.993988
	Sample Timepoint	0.083128	0.773102	0.993988
	Diet Group:Sample Timepoint	0.104777	0.746171	0.993988
Acarbose and validamycin biosynthesis				
	Diet Group	0.550068	0.45829	0.993988
	Sample Timepoint	0.586895	0.443622	0.993988
	Diet Group:Sample Timepoint	0.012221	0.911974	0.993988
Alanine, aspartate and glutamate metabolism				
	Diet Group	0.631953	0.42664	0.993988
	Sample Timepoint	0.463259	0.496105	0.993988
	Diet Group:Sample Timepoint	0.274592	0.600268	0.993988
alpha-Linolenic acid metabolism				
	Diet Group	0.278485	0.597696	0.993988
	Sample Timepoint	3.155291	0.075681	0.993988
	Diet Group:Sample Timepoint	4.840307	0.027802	0.761531
Amino acid metabolism				
	Diet Group	0.028397	0.866178	0.993988
	Sample Timepoint	0.373834	0.540922	0.993988
	Diet Group:Sample Timepoint	1.264421	0.260816	0.993988
Amino acid related enzymes				
	Diet Group	0.695294	0.40437	0.993988
	Sample Timepoint	0.180114	0.671275	0.993988
	Diet Group:Sample Timepoint	0.436014	0.509053	0.993988
Amino sugar and nucleotide sugar metabolism				
	Diet Group	0.143202	0.705119	0.993988
	Sample Timepoint	0.216753	0.641525	0.993988
	Diet Group:Sample Timepoint	0.013179	0.908602	0.993988
Aminoacyl-tR biosynthesis				
	Diet Group	0.387483	0.533625	0.993988
	Sample Timepoint	0.054016	0.816216	0.993988
	Diet Group:Sample Timepoint	0.612012	0.434031	0.993988
Aminobenzoate degradation				
	Diet Group	0.363038	0.546824	0.993988
	Sample Timepoint	0.098295	0.753885	0.993988
	Diet Group:Sample Timepoint	0.104555	0.74643	0.993988

Antimicrobial resistance genes				
	Diet Group	0.146961	0.701456	0.993988
	Sample Timepoint	0.000293	0.986345	0.994961
	Diet Group:Sample Timepoint	0.127034	0.721527	0.993988
Arachidonic acid metabolism				
	Diet Group	0.20401	0.651504	0.993988
	Sample Timepoint	0.000533	0.981576	0.994961
	Matsuda index	8.025142	0.004613	0.223565
	Diet Group:Sample Timepoint	0.04581	0.830522	0.993988
Arginine and proline metabolism				
	Diet Group	0.006965	0.933488	0.993988
	Sample Timepoint	0.191593	0.661594	0.993988
	Diet Group:Sample Timepoint	0.986309	0.320646	0.993988
Arginine biosynthesis				
	Diet Group	2.530285	0.11168	0.993988
	Sample Timepoint	0.015878	0.899725	0.993988
	Diet Group:Sample Timepoint	0.754401	0.385086	0.993988
Ascorbate and aldarate metabolism				
	Diet Group	0.630517	0.427166	0.993988
	Sample Timepoint	0.210302	0.646531	0.993988
	Diet Group:Sample Timepoint	0.131304	0.717084	0.993988
Atrazine degradation				
	Diet Group	3.573893	0.058695	0.993988
	Sample Timepoint	0.095099	0.757792	0.993988
	Diet Group:Sample Timepoint	0.809159	0.368369	0.993988
Bacterial chemotaxis				
	Diet Group	0.645072	0.42188	0.993988
	Sample Timepoint	0.024627	0.875299	0.993988
	Fasting Insulin	8.648534	0.003273	0.20301
	Diet Group:Sample Timepoint	0.710211	0.399374	0.993988
Bacterial invasion of epithelial cells				
	Diet Group	0.027962	0.867198	0.993988
	Sample Timepoint	0.051617	0.820273	0.993988
	Fasting FFAs	7.749864	0.005372	0.241725
	Diet Group:Sample Timepoint	1.455381	0.227666	0.993988
Bacterial motility proteins				
	Diet Group	0.321501	0.570707	0.993988
	Sample Timepoint	0.218978	0.639819	0.993988
	HOMA-IR	10.0864	0.001494	0.158201

	Diet Group:Sample Timepoint	0.748932	0.386814	0.993988
Bacterial secretion system				
	Diet Group	0.407457	0.523263	0.993988
	Sample Timepoint	0.000115	0.991433	0.994961
	Diet Group:Sample Timepoint	0.263582	0.60767	0.993988
Bacterial toxins				
	Diet Group	0.566845	0.451515	0.993988
	Sample Timepoint	0.004517	0.946414	0.993988
	Diet Group:Sample Timepoint	0.10276	0.748543	0.993988
Base excision repair				
	Diet Group	0.252101	0.6156	0.993988
	Sample Timepoint	0.017294	0.895374	0.993988
	Diet Group:Sample Timepoint	0.441346	0.506474	0.993988
Benzoate degradation				
	Diet Group	0.447598	0.503478	0.993988
	Sample Timepoint	1.051149	0.305243	0.993988
	Diet Group:Sample Timepoint	0.005001	0.943621	0.993988
beta-Alanine metabolism				
	Diet Group	0.923437	0.336574	0.993988
	Sample Timepoint	0.027401	0.868524	0.993988
	Diet Group:Sample Timepoint	3.007421	0.082884	0.993988
beta-Lactam resistance				
	Diet Group	0.881522	0.347785	0.993988
	Sample Timepoint	0.060315	0.805999	0.993988
	Diet Group:Sample Timepoint	0.085771	0.769624	0.993988
Betalain biosynthesis				
	Diet Group	0.382989	0.536008	0.993988
	Sample Timepoint	0.000425	0.983545	0.994961
	Diet Group:Sample Timepoint	0.061845	0.803603	0.993988
Biofilm formation - Escherichia coli				
	Diet Group	1.24366	0.264767	0.993988
	Sample Timepoint	0.169555	0.680507	0.993988
	Diet Group:Sample Timepoint	0.330896	0.565132	0.993988
Biofilm formation - Pseudomos aeruginosa				
	Diet Group	0.452791	0.501013	0.993988
	Sample Timepoint	0.852341	0.355891	0.993988
	Diet Group:Sample Timepoint	3.99E-05	0.994961	0.994961
Biofilm formation - Vibrio cholerae				
	Diet Group	0.872035	0.350393	0.993988
	Sample Timepoint	0.109131	0.741135	0.993988

	Diet Group:Sample Timepoint	0.149889	0.698641	0.993988
Biosynthesis and biodegradation of secondary metabolites				
	Diet Group	0.010243	0.919387	0.993988
	Sample Timepoint	0.723683	0.394938	0.993988
	Diet Group:Sample Timepoint	0.337845	0.561076	0.993988
Biosynthesis of ansamycins				
	Diet Group	0.608561	0.43533	0.993988
	Sample Timepoint	0.976084	0.323167	0.993988
	Diet Group:Sample Timepoint	0.103553	0.747607	0.993988
Biosynthesis of siderophore group nonribosomal peptides				
	Diet Group	0.569061	0.450632	0.993988
	Sample Timepoint	0.020137	0.887154	0.993988
	Diet Group:Sample Timepoint	0.414434	0.519728	0.993988
Biosynthesis of unsaturated fatty acids				
	Diet Group	0.540635	0.46217	0.993988
	Sample Timepoint	1.958656	0.161657	0.993988
	Diet Group:Sample Timepoint	0.675781	0.411043	0.993988
Biosynthesis of vancomycin group antibiotics				
	Diet Group	0.878866	0.348513	0.993988
	Sample Timepoint	0.521865	0.470047	0.993988
	Diet Group:Sample Timepoint	0.000843	0.976834	0.994961
Biotin metabolism				
	Diet Group	0.977063	0.322925	0.993988
	Sample Timepoint	1.757181	0.184977	0.993988
	Diet Group:Sample Timepoint	0.049583	0.82379	0.993988
Butanoate metabolism				
	Diet Group	0.047667	0.827173	0.993988
	Sample Timepoint	0.63932	0.423957	0.993988
	Diet Group:Sample Timepoint	0.124652	0.724042	0.993988
C5-Branched dibasic acid metabolism				
	Diet Group	1.260529	0.261551	0.993988
	Sample Timepoint	0.291776	0.589085	0.993988
	Diet Group:Sample Timepoint	0.079004	0.778652	0.993988
Caprolactam degradation				
	Diet Group	0.006311	0.936682	0.993988
	Sample Timepoint	0.618019	0.431785	0.993988
	Diet Group:Sample Timepoint	0.105764	0.745019	0.993988

Carbapenem biosynthesis				
	Diet Group	0.502131	0.478565	0.993988
	Sample Timepoint	0.186222	0.66608	0.993988
	Diet Group:Sample Timepoint	0.497408	0.480641	0.993988
Carbohydrate digestion and absorption				
	Diet Group	1.534802	0.215393	0.993988
	Sample Timepoint	1.021772	0.312099	0.993988
	Diet Group:Sample Timepoint	0.300947	0.583289	0.993988
Carbohydrate metabolism				
	Diet Group	0.136839	0.711444	0.993988
	Sample Timepoint	0.028005	0.867098	0.993988
	Diet Group:Sample Timepoint	0.082469	0.773978	0.993988
Carbon fixation pathways in prokaryotes				
	Diet Group	0.003009	0.956252	0.993988
	Sample Timepoint	0.000231	0.987886	0.994961
	Diet Group:Sample Timepoint	0.405264	0.524383	0.993988
Carotenoid biosynthesis				
	Diet Group	1.972542	0.160178	0.993988
	Sample Timepoint	1.180044	0.277347	0.993988
	Diet Group:Sample Timepoint	0.000535	0.981539	0.994961
Cationic antimicrobial peptide (CAMP) resistance				
	Diet Group	0.119184	0.729921	0.993988
	Sample Timepoint	8.665097	0.003244	0.20301
	Fasting Glycerol	10.76087	0.001037	0.158201
	Diet Group:Sample Timepoint	5.44941	0.019575	0.608512
Cell growth				
	Diet Group	0.598464	0.439165	0.993988
	Sample Timepoint	0.031192	0.859813	0.993988
	Diet Group:Sample Timepoint	1.097387	0.29484	0.993988
Cell motility and secretion				
	Diet Group	0.012466	0.911101	0.993988
	Sample Timepoint	0.061842	0.803608	0.993988
	Diet Group:Sample Timepoint	0.027738	0.867726	0.993988
Chaperones and folding catalysts				
	Diet Group	0.336136	0.562068	0.993988
	Sample Timepoint	1.931682	0.164575	0.993988
	Diet Group:Sample Timepoint	0.009284	0.92324	0.993988
Chloroalkane and chloroalkene degradation				
	Diet Group	0.227319	0.633519	0.993988

	Sample Timepoint	0.195063	0.658736	0.993988
	Diet Group:Sample Timepoint	0.001133	0.97315	0.994961
Chlorocyclohexane and chlorobenzene degradation				
	Diet Group	0.036925	0.847618	0.993988
	Sample Timepoint	0.501353	0.478906	0.993988
	Diet Group:Sample Timepoint	1.268171	0.26011	0.993988
Cholesterol metabolism				
	Diet Group	0.632217	0.426543	0.993988
	Sample Timepoint	2.812329	0.093543	0.993988
	Diet Group:Sample Timepoint	0.730979	0.392566	0.993988
Chromosome and associated proteins				
	Diet Group	0.298651	0.584729	0.993988
	Sample Timepoint	0.028483	0.865977	0.993988
	Fasting Glycerol	0.190858	0.662203	0.993988
	Diet Group:Sample Timepoint	0.551979	0.45751	0.993988
Citrate cycle (TCA cycle)				
	Diet Group	0.899638	0.342879	0.993988
	Sample Timepoint	0.246234	0.61974	0.993988
	Diet Group:Sample Timepoint	0.312879	0.575919	0.993988
Cyanoamino acid metabolism				
	Diet Group	0.056565	0.81201	0.993988
	Sample Timepoint	0.095087	0.757807	0.993988
	Diet Group:Sample Timepoint	0.038972	0.843505	0.993988
Cysteine and methionine metabolism				
	Diet Group	0.457321	0.498879	0.993988
	Sample Timepoint	0.131851	0.716521	0.993988
	Diet Group:Sample Timepoint	0.205045	0.650679	0.993988
Cytoskeleton proteins				
	Diet Group	0.314257	0.57508	0.993988
	Sample Timepoint	0.068619	0.793358	0.993988
	Diet Group:Sample Timepoint	0.497503	0.480599	0.993988
D-Alanine metabolism				
	Diet Group	0.237067	0.626332	0.993988
	Sample Timepoint	0.187443	0.665054	0.993988
	Diet Group:Sample Timepoint	0.002813	0.9577	0.993988
D-Arginine and D-ornithine metabolism				
	Diet Group	0.050543	0.82212	0.993988
	Sample Timepoint	0.048486	0.825719	0.993988
	Diet Group:Sample Timepoint	0.001874	0.965469	0.994961

D-Glutamine and D-glutamate metabolism				
	Diet Group	0.013869	0.906253	0.993988
	Sample Timepoint	1.287031	0.256596	0.993988
	Diet Group:Sample Timepoint	0.142297	0.706008	0.993988
Dioxin degradation				
	Diet Group	0.237261	0.626192	0.993988
	Sample Timepoint	0.062591	0.802446	0.993988
	Fasting Insulin	7.393626	0.006546	0.24257
	Diet Group:Sample Timepoint	0.198239	0.656146	0.993988
D repair and recombition proteins				
	Diet Group	0.282782	0.594884	0.993988
	Sample Timepoint	0.016325	0.898332	0.993988
	Diet Group:Sample Timepoint	0.553416	0.456925	0.993988
D replication proteins				
	Diet Group	0.036517	0.848452	0.993988
	Sample Timepoint	0.060726	0.805352	0.993988
	Diet Group:Sample Timepoint	0.616211	0.432459	0.993988
Energy metabolism				
	Diet Group	0.003824	0.95069	0.993988
	Sample Timepoint	0.786204	0.37525	0.993988
	Diet Group:Sample Timepoint	1.268759	0.26	0.993988
Ether lipid metabolism				
	Diet Group	0.829747	0.362346	0.993988
	Sample Timepoint	0.116696	0.732646	0.993988
	Diet Group:Sample Timepoint	0.251646	0.615919	0.993988
Exosome				
	Diet Group	1.197064	0.273909	0.993988
	Sample Timepoint	0.965236	0.325871	0.993988
	Diet Group:Sample Timepoint	0.091123	0.762754	0.993988
Fatty acid biosynthesis				
	Diet Group	0.577249	0.447393	0.993988
	Sample Timepoint	0.29998	0.583895	0.993988
	Diet Group:Sample Timepoint	0.5401	0.462391	0.993988
Fatty acid degradation				
	Diet Group	0.267817	0.6048	0.993988
	Sample Timepoint	0.535536	0.464289	0.993988
	Diet Group:Sample Timepoint	0.133752	0.714573	0.993988
Fatty acid elongation				
	Diet Group	0.014105	0.905462	0.993988
	Sample Timepoint	0.927731	0.335453	0.993988
	Diet Group:Sample Timepoint	1.397777	0.237096	0.993988

Flagellar assembly				
	Diet Group	0.080947	0.776018	0.993988
	Sample Timepoint	0.042543	0.836588	0.993988
	Matsuda index	12.28544	0.000457	0.158201
	Diet Group:Sample Timepoint	0.351402	0.55332	0.993988
Flavone and flavonol biosynthesis				
	Diet Group	1.536785	0.215097	0.993988
	Sample Timepoint	0.980125	0.322168	0.993988
	Fasting Triglycerides	6.981544	0.008235	0.27307
	Diet Group:Sample Timepoint	0.28381	0.594215	0.993988
Flavonoid biosynthesis				
	Diet Group	0.637415	0.424648	0.993988
	Sample Timepoint	0.069733	0.791726	0.993988
	Diet Group:Sample Timepoint	0.133404	0.714929	0.993988
Fluorobenzoate degradation				
	Diet Group	0.730991	0.392562	0.993988
	Sample Timepoint	0.175251	0.675487	0.993988
	Fasting Insulin	8.789303	0.00303	0.20301
	Diet Group:Sample Timepoint	0.910195	0.340063	0.993988
Folate biosynthesis				
	Diet Group	0.724034	0.394824	0.993988
	Sample Timepoint	0.000307	0.986015	0.994961
	Diet Group:Sample Timepoint	0.123661	0.725097	0.993988
Fructose and mannose metabolism				
	Diet Group	3.828441	0.05039	0.993988
	Sample Timepoint	0.73357	0.391729	0.993988
	Fasting Triglycerides	8.345301	0.003867	0.20301
	Diet Group:Sample Timepoint	0.379876	0.53767	0.993988
Galactose metabolism				
	Diet Group	2.981658	0.084213	0.993988
	Sample Timepoint	0.024523	0.875562	0.993988
	Diet Group:Sample Timepoint	0.004531	0.946331	0.993988
Geraniol degradation				
	Diet Group	0.021085	0.884548	0.993988
	Sample Timepoint	0.006645	0.935031	0.993988
	Diet Group:Sample Timepoint	0.340305	0.559653	0.993988
Glucagon sigling pathway				
	Diet Group	0.710247	0.399362	0.993988
	Sample Timepoint	1.424748	0.232623	0.993988
	Materl BMI	7.152441	0.007486	0.262017
	Diet Group:Sample Timepoint	0.003183	0.955008	0.993988

Glucosinolate biosynthesis				
	Diet Group	1.595996	0.206472	0.993988
	Sample Timepoint	0.217754	0.640756	0.993988
	Diet Group:Sample Timepoint	0.14197	0.706331	0.993988
Glutathione metabolism				
	Diet Group	0.538229	0.463168	0.993988
	Sample Timepoint	2.333967	0.126579	0.993988
	Diet Group:Sample Timepoint	1.039159	0.308017	0.993988
Glycan biosynthesis and metabolism				
	Diet Group	0.031312	0.859546	0.993988
	Sample Timepoint	0.288172	0.591394	0.993988
	Diet Group:Sample Timepoint	0.009722	0.921454	0.993988
Glycerolipid metabolism				
	Diet Group	1.849514	0.17384	0.993988
	Sample Timepoint	4.88E-05	0.994428	0.994961
	Diet Group:Sample Timepoint	0.658714	0.417014	0.993988
Glycerophospholipid metabolism				
	Diet Group	0.6567	0.417728	0.993988
	Sample Timepoint	0.023942	0.877033	0.993988
	Diet Group:Sample Timepoint	0.832501	0.36155	0.993988
Glycine, serine and threonine metabolism				
	Diet Group	0.066963	0.795811	0.993988
	Sample Timepoint	2.251778	0.133461	0.993988
	Diet Group:Sample Timepoint	0.465421	0.495101	0.993988
Glycolysis / Gluconeogenesis				
	Diet Group	0.677952	0.410293	0.993988
	Sample Timepoint	0.531707	0.46589	0.993988
	Diet Group:Sample Timepoint	0.077583	0.7806	0.993988
Glycosaminoglycan binding proteins				
	Diet Group	0.045794	0.830551	0.993988
	Sample Timepoint	0.038521	0.844401	0.993988
	Diet Group:Sample Timepoint	0.19564	0.658263	0.993988
Glycosaminoglycan degradation				
	Diet Group	0.579952	0.446331	0.993988
	Sample Timepoint	0.420273	0.516801	0.993988
	Diet Group:Sample Timepoint	0.721915	0.395517	0.993988
Glycosylphosphatidylinositol (GPI)-anchored proteins				
	Diet Group	0.032724	0.856448	0.993988

	Sample Timepoint	0.000103	0.991911	0.994961
	Diet Group:Sample Timepoint	0.042829	0.836047	0.993988
Glycosyltransferases				
	Diet Group	0.012514	0.91093	0.993988
	Sample Timepoint	2.962232	0.08523	0.993988
	Diet Group:Sample Timepoint	0.198858	0.655644	0.993988
Glyoxylate and dicarboxylate metabolism				
	Diet Group	0.015582	0.900661	0.993988
	Sample Timepoint	2.478554	0.115408	0.993988
	Diet Group:Sample Timepoint	1.14488	0.284623	0.993988
Histidine metabolism				
	Diet Group	0.026539	0.87059	0.993988
	Sample Timepoint	1.25109	0.263344	0.993988
	Diet Group:Sample Timepoint	0.657378	0.417487	0.993988
Homologous recombition				
	Diet Group	0.043899	0.834042	0.993988
	Sample Timepoint	0.716061	0.397439	0.993988
	Diet Group:Sample Timepoint	0.334232	0.563178	0.993988
Inorganic ion transport and metabolism				
	Diet Group	0.074459	0.784952	0.993988
	Sample Timepoint	1.63851	0.20053	0.993988
	Diet Group:Sample Timepoint	3.857241	0.049532	0.993988
Inositol phosphate metabolism				
	Diet Group	1.946517	0.162963	0.993988
	Sample Timepoint	0.662457	0.415694	0.993988
	Diet Group:Sample Timepoint	0.364243	0.546159	0.993988
Isoquinoline alkaloid biosynthesis				
	Diet Group	0.324179	0.569107	0.993988
	Sample Timepoint	2.19181	0.138746	0.993988
	Diet Group:Sample Timepoint	0.114016	0.735618	0.993988
Limonene and pinene degradation				
	Diet Group	0.006311	0.936682	0.993988
	Sample Timepoint	0.618019	0.431785	0.993988
	Diet Group:Sample Timepoint	0.105764	0.745019	0.993988
Linoleic acid metabolism				
	Diet Group	0.278485	0.597696	0.993988
	Sample Timepoint	3.155291	0.075681	0.993988
	Diet Group:Sample Timepoint	4.840307	0.027802	0.761531
Lipid biosynthesis proteins				

	Diet Group	0.306463	0.579859	0.993988
	Sample Timepoint	0.38441	0.535252	0.993988
	Diet Group:Sample Timepoint	0.284045	0.594062	0.993988
Lipid metabolism				
	Diet Group	0.764129	0.382039	0.993988
	Sample Timepoint	0.444685	0.50487	0.993988
	Diet Group:Sample Timepoint	0.039367	0.842724	0.993988
Lipoic acid metabolism				
	Diet Group	2.024379	0.154792	0.993988
	Sample Timepoint	0.972537	0.324048	0.993988
	Diet Group:Sample Timepoint	2.333806	0.126592	0.993988
Lipopolysaccharide biosynthesis				
	Diet Group	0.360068	0.548469	0.993988
	Sample Timepoint	0.003356	0.953802	0.993988
	Diet Group:Sample Timepoint	0.083872	0.772118	0.993988
Lipopolysaccharide biosynthesis proteins				
	Diet Group	0.228158	0.632893	0.993988
	Sample Timepoint	0.083143	0.773082	0.993988
	Diet Group:Sample Timepoint	0.13412	0.714198	0.993988
Lysine biosynthesis				
	Diet Group	1.065071	0.302062	0.993988
	Sample Timepoint	0.007222	0.932276	0.993988
	Diet Group:Sample Timepoint	0.600477	0.438396	0.993988
Lysine degradation				
	Diet Group	0.015586	0.900646	0.993988
	Sample Timepoint	0.046469	0.829326	0.993988
	Diet Group:Sample Timepoint	0.04982	0.823377	0.993988
Lysosome				
	Diet Group	0.39506	0.529651	0.993988
	Sample Timepoint	0.17273	0.677697	0.993988
	Diet Group:Sample Timepoint	0.246532	0.619528	0.993988
Mannose type O-glycan biosyntheis				
	Diet Group	0.000141	0.990531	0.994961
	Sample Timepoint	0.789334	0.374301	0.993988
	Diet Group:Sample Timepoint	0.034345	0.852976	0.993988
Membrane and intracellular structural molecules				
	Diet Group	0.004688	0.94541	0.993988
	Sample Timepoint	0.780227	0.377072	0.993988
	Diet Group:Sample Timepoint	0.33756	0.561241	0.993988

Membrane trafficking				
	Diet Group	4.096727	0.042966	0.993988
	Sample Timepoint	0.273405	0.601057	0.993988
	Diet Group:Sample Timepoint	1.357818	0.243916	0.993988
Messenger R Biogenesis				
	Diet Group	0.520263	0.470729	0.993988
	Sample Timepoint	0.329551	0.565924	0.993988
	Diet Group:Sample Timepoint	0.224918	0.635318	0.993988
Metabolism of cofactors and vitamins				
	Diet Group	1.09221	0.295982	0.993988
	Sample Timepoint	0.659699	0.416666	0.993988
	Diet Group:Sample Timepoint	0.012287	0.911737	0.993988
Metabolism of xenobiotics by cytochrome P450				
	Diet Group	0.816815	0.366113	0.993988
	Sample Timepoint	2.635243	0.104516	0.993988
	Diet Group:Sample Timepoint	2.344988	0.125687	0.993988
Methane metabolism				
	Diet Group	2.626022	0.105125	0.993988
	Sample Timepoint	0.177822	0.673252	0.993988
	Diet Group:Sample Timepoint	0.126573	0.722012	0.993988
Mineral absorption				
	Diet Group	1.113935	0.291228	0.993988
	Sample Timepoint	0.007426	0.931329	0.993988
	Diet Group:Sample Timepoint	0.097838	0.75444	0.993988
Mismatch repair				
	Diet Group	0.019276	0.889577	0.993988
	Sample Timepoint	0.713389	0.398321	0.993988
	Diet Group:Sample Timepoint	0.255887	0.61296	0.993988
Monobactam biosynthesis				
	Diet Group	1.297876	0.254602	0.993988
	Sample Timepoint	0.163497	0.685957	0.993988
	Diet Group:Sample Timepoint	0.132687	0.715662	0.993988
N-Glycan biosynthesis				
	Diet Group	0.54221	0.461518	0.993988
	Sample Timepoint	0.212173	0.645069	0.993988
	Diet Group:Sample Timepoint	0.235311	0.627613	0.993988
phthalene degradation				
	Diet Group	7.51E-05	0.993084	0.994961
	Sample Timepoint	0.176758	0.674174	0.993988
	Diet Group:Sample Timepoint	0.03373	0.854283	0.993988

Neomycin, kamycin and gentamicin biosynthesis				
	Diet Group	0.257889	0.611574	0.993988
	Sample Timepoint	0.454843	0.500044	0.993988
	Diet Group:Sample Timepoint	2.363519	0.124202	0.993988
Nicotite and nicotimide metabolism				
	Diet Group	1.151942	0.283143	0.993988
	Sample Timepoint	0.264741	0.606882	0.993988
	Diet Group:Sample Timepoint	0.367719	0.544251	0.993988
Nitrogen metabolism				
	Diet Group	1.353754	0.244623	0.993988
	Sample Timepoint	0.024174	0.876444	0.993988
	Diet Group:Sample Timepoint	1.029259	0.310333	0.993988
Nitrotoluene degradation				
	Diet Group	0.012042	0.912617	0.993988
	Sample Timepoint	0.479057	0.48885	0.993988
	Diet Group:Sample Timepoint	0.002887	0.957148	0.993988
Non-homologous end-joining				
	Diet Group	0.164855	0.684726	0.993988
	Sample Timepoint	1.939488	0.163724	0.993988
	Diet Group:Sample Timepoint	0.215162	0.642751	0.993988
Nonribosomal peptide structures				
	Diet Group	0.377415	0.53899	0.993988
	Sample Timepoint	1.386102	0.239064	0.993988
	Diet Group:Sample Timepoint	1.359593	0.243608	0.993988
Novobiocin biosynthesis				
	Diet Group	0.719987	0.396148	0.993988
	Sample Timepoint	0.699298	0.40302	0.993988
	Diet Group:Sample Timepoint	0.016751	0.897021	0.993988
Nucleotide excision repair				
	Diet Group	0.100688	0.751005	0.993988
	Sample Timepoint	0.011665	0.913993	0.993988
	Diet Group:Sample Timepoint	0.56893	0.450685	0.993988
Nucleotide metabolism				
	Diet Group	0.169208	0.680817	0.993988
	Sample Timepoint	0.27007	0.603285	0.993988
	Diet Group:Sample Timepoint	0.119854	0.729193	0.993988
One carbon pool by folate				
	Diet Group	0.196625	0.657459	0.993988
	Sample Timepoint	0.93467	0.333652	0.993988

	Diet Group:Sample Timepoint	0.554562	0.45646	0.993988
Other glycan degradation				
	Diet Group	0.091193	0.762666	0.993988
	Sample Timepoint	0.004295	0.947744	0.993988
	Diet Group:Sample Timepoint	0.064344	0.799757	0.993988
Other types of O-glycan biosynthesis				
	Diet Group	0.000141	0.990531	0.994961
	Sample Timepoint	0.789334	0.374301	0.993988
	Diet Group:Sample Timepoint	0.034345	0.852976	0.993988
Oxidative phosphorylation				
	Diet Group	2.893365	0.088945	0.993988
	Sample Timepoint	0.165806	0.683866	0.993988
	Diet Group:Sample Timepoint	0.803186	0.370142	0.993988
Pantothete and CoA biosynthesis				
	Diet Group	2.255115	0.133174	0.993988
	Sample Timepoint	0.001717	0.966951	0.994961
	Diet Group:Sample Timepoint	1.182045	0.27694	0.993988
Penicillin and cephalosporin biosynthesis				
	Diet Group	1.524485	0.216942	0.993988
	Sample Timepoint	1.966637	0.160805	0.993988
	Diet Group:Sample Timepoint	0.993223	0.318956	0.993988
Pentose and glucurote interconversions				
	Diet Group	1.243388	0.264819	0.993988
	Sample Timepoint	1.384021	0.239417	0.993988
	Fasting Triglycerides	11.82177	0.000585	0.158201
	Diet Group:Sample Timepoint	0.054087	0.816099	0.993988
Pentose phosphate pathway				
	Diet Group	2.075583	0.149673	0.993988
	Sample Timepoint	0.358347	0.549426	0.993988
	Diet Group:Sample Timepoint	0.075002	0.784188	0.993988
Peptidases				
	Diet Group	0.413506	0.520196	0.993988
	Sample Timepoint	0.216873	0.641433	0.993988
	Diet Group:Sample Timepoint	0.513787	0.473504	0.993988
Peptidoglycan biosynthesis and degradation proteins				
	Diet Group	0.693536	0.404964	0.993988
	Sample Timepoint	0.258151	0.611394	0.993988
	Diet Group:Sample Timepoint	0.163118	0.686302	0.993988
Peroxisome				

	Diet Group	1.50621	0.219718	0.993988
	Sample Timepoint	1.189404	0.27545	0.993988
	Diet Group:Sample Timepoint	1.362979	0.243022	0.993988
Phezine biosynthesis				
	Diet Group	0.010538	0.918237	0.993988
	Sample Timepoint	1.257018	0.262216	0.993988
	Diet Group:Sample Timepoint	0.008362	0.927142	0.993988
Phenylalanine metabolism				
	Diet Group	0.167551	0.682297	0.993988
	Sample Timepoint	0.442372	0.50598	0.993988
	Diet Group:Sample Timepoint	0.84237	0.35872	0.993988
Phenylalanine, tyrosine and tryptophan biosynthesis				
	Diet Group	1.926232	0.165171	0.993988
	Sample Timepoint	0.152554	0.696106	0.993988
	Diet Group:Sample Timepoint	1.036812	0.308564	0.993988
Phenylpropanoid biosynthesis				
	Diet Group	0.03309	0.855656	0.993988
	Sample Timepoint	0.28391	0.59415	0.993988
	Diet Group:Sample Timepoint	0.106333	0.744358	0.993988
Phosphote and phosphite metabolism				
	Diet Group	0.018489	0.891841	0.993988
	Sample Timepoint	0.297133	0.585685	0.993988
	Diet Group:Sample Timepoint	0.009273	0.923284	0.993988
Phosphotransferase system (PTS)				
	Diet Group	1.052028	0.305041	0.993988
	Sample Timepoint	0.436593	0.508771	0.993988
	Diet Group:Sample Timepoint	0.302262	0.582468	0.993988
Polyketide biosynthesis proteins				
	Diet Group	1.509666	0.21919	0.993988
	Sample Timepoint	0.029846	0.86284	0.993988
	Diet Group:Sample Timepoint	0.840871	0.359148	0.993988
Polyketide sugar unit biosynthesis				
	Diet Group	0.73964	0.389776	0.993988
	Sample Timepoint	0.579683	0.446437	0.993988
	Diet Group:Sample Timepoint	4.05E-05	0.994925	0.994961
Porphyrin and chlorophyll metabolism				
	Diet Group	4.215544	0.040055	0.993988

	Sample Timepoint	0.123941	0.724799	0.993988
	Diet Group:Sample Timepoint	0.213033	0.6444	0.993988
Prenyltransferases				
	Diet Group	0.232363	0.629777	0.993988
	Sample Timepoint	0.399505	0.527345	0.993988
	Diet Group:Sample Timepoint	0.132498	0.715856	0.993988
Prodigiosin biosyntheses				
	Diet Group	0.250921	0.616427	0.993988
	Sample Timepoint	1.294654	0.255192	0.993988
	Diet Group:Sample Timepoint	0.055409	0.813905	0.993988
Prokaryotic Defense System				
	Diet Group	0.989123	0.319957	0.993988
	Sample Timepoint	1.771868	0.183151	0.993988
	Diet Group:Sample Timepoint	7.527906	0.006075	0.24257
Propanoate metabolism				
	Diet Group	2.236201	0.134812	0.993988
	Sample Timepoint	0.12981	0.718629	0.993988
	Diet Group:Sample Timepoint	0.082144	0.774412	0.993988
Proteasome				
	Diet Group	0.162021	0.687303	0.993988
	Sample Timepoint	3.372116	0.066309	0.993988
	Diet Group:Sample Timepoint	0.633556	0.426054	0.993988
Protein digestion and absorption				
	Diet Group	0.906995	0.340913	0.993988
	Sample Timepoint	0.104532	0.746458	0.993988
	Diet Group:Sample Timepoint	0.043633	0.834538	0.993988
Protein export				
	Diet Group	1.148604	0.283842	0.993988
	Sample Timepoint	0.006562	0.935438	0.993988
	Diet Group:Sample Timepoint	1.168058	0.279801	0.993988
Protein folding and associated processing				
	Diet Group	3.184107	0.074358	0.993988
	Sample Timepoint	0.000391	0.984225	0.994961
	Diet Group:Sample Timepoint	0.593058	0.441239	0.993988
Protein kises				
	Diet Group	1.38508	0.239238	0.993988
	Sample Timepoint	0.143637	0.704692	0.993988
	Diet Group:Sample Timepoint	0.015481	0.900982	0.993988
Protein phosphatase and associated proteins				
	Diet Group	1.452438	0.228137	0.993988

	Sample Timepoint	0.578201	0.447019	0.993988
	Diet Group:Sample Timepoint	0.045304	0.831446	0.993988
Purine metabolism				
	Diet Group	0.872213	0.350344	0.993988
	Sample Timepoint	0.061318	0.804425	0.993988
	Diet Group:Sample Timepoint	0.756525	0.384418	0.993988
Pyrimidine metabolism				
	Diet Group	0.295607	0.58665	0.993988
	Sample Timepoint	0.324078	0.569167	0.993988
	Diet Group:Sample Timepoint	1.050348	0.305427	0.993988
Pyruvate metabolism				
	Diet Group	0.868021	0.351504	0.993988
	Sample Timepoint	0.173798	0.676758	0.993988
	Diet Group:Sample Timepoint	0.188576	0.664104	0.993988
Quorum sensing				
	Diet Group	0.954811	0.328498	0.993988
	Sample Timepoint	0.03754	0.846369	0.993988
	Diet Group:Sample Timepoint	0.214569	0.64321	0.993988
Replication, recombition and repair proteins				
	Diet Group	0.714063	0.398098	0.993988
	Sample Timepoint	0.096321	0.75629	0.993988
	Diet Group:Sample Timepoint	0.945036	0.330986	0.993988
Retinol metabolism				
	Diet Group	0.028462	0.866027	0.993988
	Sample Timepoint	0.501157	0.478992	0.993988
	Diet Group:Sample Timepoint	0.042125	0.837381	0.993988
Riboflavin metabolism				
	Diet Group	0.064101	0.800127	0.993988
	Sample Timepoint	0.318238	0.572668	0.993988
	Diet Group:Sample Timepoint	0.36502	0.545731	0.993988
Ribosome biogenesis				
	Diet Group	0.400118	0.527028	0.993988
	Sample Timepoint	0.049276	0.824327	0.993988
	Diet Group:Sample Timepoint	0.811178	0.367772	0.993988
R degradation				
	Diet Group	0.341455	0.558991	0.993988
	Sample Timepoint	0.072439	0.787819	0.993988
	Diet Group:Sample Timepoint	0.806486	0.369161	0.993988
R polymerase				
	Diet Group	0.150683	0.697884	0.993988
	Sample Timepoint	0.49412	0.482095	0.993988
	Diet Group:Sample Timepoint	0.013169	0.908639	0.993988

R transport				
	Diet Group	1.915758	0.166325	0.993988
	Sample Timepoint	0.006965	0.933489	0.993988
	Diet Group:Sample Timepoint	2.775913	0.095692	0.993988
Salmonella infection				
	Diet Group	1.913222	0.166606	0.993988
	Sample Timepoint	0.150749	0.697821	0.993988
	Fasting Triglycerides	8.363768	0.003828	0.20301
	Diet Group:Sample Timepoint	0.06773	0.794671	0.993988
Secondary bile acid biosynthesis				
	Diet Group	0.090356	0.763725	0.993988
	Sample Timepoint	0.032836	0.856206	0.993988
	Diet Group:Sample Timepoint	0.430672	0.51166	0.993988
Secretion system				
	Diet Group	0.107473	0.74304	0.993988
	Sample Timepoint	0.199733	0.654937	0.993988
	Diet Group:Sample Timepoint	0.058671	0.808609	0.993988
Selenocompound metabolism				
	Diet Group	0.079533	0.777932	0.993988
	Sample Timepoint	1.143622	0.284888	0.993988
	Diet Group:Sample Timepoint	0.061074	0.804807	0.993988
Sesquiterpenoid and triterpenoid biosynthesis				
	Diet Group	0.209685	0.647014	0.993988
	Sample Timepoint	0.006759	0.934476	0.993988
	Diet Group:Sample Timepoint	0.434144	0.509963	0.993988
Sigl transduction mechanisms				
	Diet Group	0.306789	0.579657	0.993988
	Sample Timepoint	0.013021	0.909151	0.993988
	Diet Group:Sample Timepoint	0.025899	0.872147	0.993988
Sphingolipid metabolism				
	Diet Group	0.001052	0.974131	0.994961
	Sample Timepoint	0.003814	0.950757	0.993988
	Diet Group:Sample Timepoint	0.036677	0.848124	0.993988
Staphylococcus aureus infection				
	Diet Group	0.159435	0.689677	0.993988
	Sample Timepoint	1.721117	0.189549	0.993988
	Fasting Insulin	10.45135	0.001226	0.158201
	Diet Group:Sample Timepoint	3.378571	0.066049	0.993988

Starch and sucrose metabolism				
	Diet Group	0.766552	0.381286	0.993988
	Sample Timepoint	0.226662	0.63401	0.993988
	Diet Group:Sample Timepoint	0.153527	0.695187	0.993988
Stilbenoid, diarylheptanoid and gingerol biosynthesis				
	Diet Group	0.637415	0.424648	0.993988
	Sample Timepoint	0.069733	0.791726	0.993988
	Diet Group:Sample Timepoint	0.133404	0.714929	0.993988
Streptomycin biosynthesis				
	Diet Group	0.394953	0.529707	0.993988
	Sample Timepoint	0.337221	0.561438	0.993988
	Diet Group:Sample Timepoint	0.166634	0.683121	0.993988
Styrene degradation				
	Diet Group	0.172128	0.678228	0.993988
	Sample Timepoint	0.441613	0.506345	0.993988
	Diet Group:Sample Timepoint	0.693935	0.404829	0.993988
Sulfur metabolism				
	Diet Group	0.509488	0.475361	0.993988
	Sample Timepoint	0.16273	0.686656	0.993988
	Diet Group:Sample Timepoint	0.37796	0.538697	0.993988
Sulfur relay system				
	Diet Group	1.752409	0.185574	0.993988
	Sample Timepoint	0.002376	0.961123	0.994961
	Diet Group:Sample Timepoint	0.630512	0.427168	0.993988
Synthesis and degradation of ketone bodies				
	Diet Group	0.572743	0.449171	0.993988
	Sample Timepoint	0.151107	0.697479	0.993988
	Diet Group:Sample Timepoint	0.027281	0.868811	0.993988
Taurine and hypotaurine metabolism				
	Diet Group	0.174643	0.676018	0.993988
	Sample Timepoint	0.12052	0.728471	0.993988
	Diet Group:Sample Timepoint	0.085835	0.76954	0.993988
Terpenoid backbone biosynthesis				
	Diet Group	0.843133	0.358502	0.993988
	Sample Timepoint	0.003907	0.95016	0.993988
	Diet Group:Sample Timepoint	2.229726	0.135378	0.993988
Thiamine metabolism				
	Diet Group	1.493863	0.221618	0.993988
	Sample Timepoint	0.042637	0.836411	0.993988

	Diet Group:Sample Timepoint	1.285937	0.256798	0.993988
Toluene degradation				
	Diet Group	0.730991	0.392562	0.993988
	Sample Timepoint	0.175251	0.675487	0.993988
	Fasting Insulin	8.789303	0.00303	0.20301
	Diet Group:Sample Timepoint	0.910195	0.340063	0.993988
Transcription factors				
	Diet Group	0.577525	0.447284	0.993988
	Sample Timepoint	0.181667	0.669945	0.993988
	Diet Group:Sample Timepoint	0.064743	0.799151	0.993988
Transcription machinery				
	Diet Group	0.198464	0.655964	0.993988
	Sample Timepoint	3.458471	0.062928	0.993988
	Fasting Glycerol	10.07043	0.001507	0.158201
	Diet Group:Sample Timepoint	0.115739	0.733703	0.993988
Transcription related proteins				
	Diet Group	0.382364	0.536341	0.993988
	Sample Timepoint	1.615173	0.203766	0.993988
	Diet Group:Sample Timepoint	1.775507	0.182702	0.993988
Transfer R biogenesis				
	Diet Group	0.509575	0.475323	0.993988
	Sample Timepoint	0.000683	0.979146	0.994961
	Diet Group:Sample Timepoint	0.890532	0.345333	0.993988
Translation factors				
	Diet Group	0.314355	0.57502	0.993988
	Sample Timepoint	0.078803	0.778926	0.993988
	Diet Group:Sample Timepoint	2.154836	0.142122	0.993988
Translation proteins				
	Diet Group	2.70848	0.099816	0.993988
	Sample Timepoint	0.558115	0.455021	0.993988
	Diet Group:Sample Timepoint	0.973555	0.323795	0.993988
Transport				
	Diet Group	0.229653	0.631781	0.993988
	Sample Timepoint	0.161071	0.688172	0.993988
	Diet Group:Sample Timepoint	0.531608	0.465932	0.993988
Transporters				
	Diet Group	1.478621	0.22399	0.993988
	Sample Timepoint	0.178221	0.672906	0.993988
	Diet Group:Sample Timepoint	0.068831	0.793047	0.993988
Tropane, piperidine and pyridine alkaloid biosynthesis				

	Diet Group	0.424837	0.514533	0.993988
	Sample Timepoint	1.247284	0.264072	0.993988
	Diet Group:Sample Timepoint	0.036017	0.84948	0.993988
Tryptophan metabolism				
	Diet Group	0.03552	0.85051	0.993988
	Sample Timepoint	0.402011	0.526052	0.993988
	Diet Group:Sample Timepoint	0.303824	0.581495	0.993988
Two-component system				
	Diet Group	1.151288	0.28328	0.993988
	Sample Timepoint	0.004794	0.944802	0.993988
	Diet Group:Sample Timepoint	0.353239	0.552285	0.993988
Tyrosine metabolism				
	Diet Group	0.183584	0.668311	0.993988
	Sample Timepoint	0.451715	0.501521	0.993988
	Diet Group:Sample Timepoint	0.240944	0.623525	0.993988
Ubiquinone and other terpenoid-quinone biosynthesis				
	Diet Group	0.460167	0.497546	0.993988
	Sample Timepoint	0.270033	0.60331	0.993988
	Diet Group:Sample Timepoint	0.826108	0.3634	0.993988
Ubiquitin system				
	Diet Group	0.682497	0.408728	0.993988
	Sample Timepoint	4.595342	0.032059	0.841547
	Diet Group:Sample Timepoint	5.387308	0.020284	0.608512
Valine, leucine and isoleucine biosynthesis				
	Diet Group	1.044255	0.306834	0.993988
	Sample Timepoint	0.258332	0.611269	0.993988
	Diet Group:Sample Timepoint	0.212716	0.644647	0.993988
Valine, leucine and isoleucine degradation				
	Diet Group	0.002823	0.95763	0.993988
	Sample Timepoint	0.583901	0.444787	0.993988
	Diet Group:Sample Timepoint	0.01687	0.896657	0.993988
Vancomycin resistance				
	Diet Group	0.113721	0.735947	0.993988
	Sample Timepoint	0.000248	0.987433	0.994961
	Diet Group:Sample Timepoint	0.17312	0.677354	0.993988
Various types of N-glycan biosynthesis				
	Diet Group	0.739542	0.389808	0.993988
	Sample Timepoint	0.517705	0.471822	0.993988
	Diet Group:Sample Timepoint	0.510376	0.474976	0.993988

Vitamin B6 metabolism				
	Diet Group	0.82428	0.363932	0.993988
	Sample Timepoint	1.22605	0.268177	0.993988
	Diet Group:Sample Timepoint	0.48715	0.485201	0.993988
Xylene degradation				
	Diet Group	0.237261	0.626192	0.993988
	Sample Timepoint	0.062591	0.802446	0.993988
	Fasting Insulin	7.393626	0.006546	0.24257
	Diet Group:Sample Timepoint	0.198239	0.656146	0.993988
Zeatin biosynthesis				
	Diet Group	0.1344	0.713913	0.993988
	Sample Timepoint	0.027929	0.867275	0.993988
	Diet Group:Sample Timepoint	0.188613	0.664073	0.993988

Supplementary Table 5. Infant family-level results for the negative binomial regression models.

	comparison	chisq	pval	adjpval
Micrococcaceae				
	Diet group	0.000246685	0.987468769	0.999984565
	Infant age	2.594727146	0.273251251	0.614815315
	Diet group:Infant age	0.312626128	0.855291388	0.999984565
Bifidobacteriaceae				
	Diet group	0.536775955	0.463771955	0.859342152
	Infant age	0.487851819	0.783545685	0.999984565
	Diet group:Infant age	1.758553736	0.415082963	0.792431111
Coriobacteriaceae				
	Diet group	0.175318753	0.675427485	0.989188576
	Infant age	10.68421645	0.004785771	0.025125296
	Diet group:Infant age	2.976592384	0.225756975	0.568907576
Bacteroidaceae				
	Diet group	1.17E-06	0.999137832	0.999984565
	Infant age	0.000196268	0.999901871	0.999984565
Breastfeeding status		4.82E-05	0.99997592	0.999984565
	Diet group:Infant age	3.09E-05	0.999984565	0.999984565
Porphyromonadaceae				
	Diet group	0.2520574	0.61563011	0.941067137
	Infant age	2.647633759	0.266117622	0.614815315
	Diet group:Infant age	2.390630975	0.30260847	0.628024363
Staphylococcaceae				
	Diet group	0.001330319	0.970904783	0.999984565
	Infant age	37.3324723	7.82E-09	2.46E-07
	Diet group:Infant age	1.273544863	0.528997046	0.877021418
Enterococcaceae				
	Diet group	13.14847965	0.000287753	0.002014268
	Infant age	3.316499301	0.190472081	0.521727875
	Gestational weight gain	6.649867839	0.009916318	0.042504482
	Diet group:Infant age	5.735321023	0.056831728	0.179019944
Lactobacillaceae				
	Diet group	0.005847457	0.939046243	0.999984565
	Infant age	2.418982197	0.298349071	0.628024363
	Delivery mode	9.364927365	0.002211768	0.012667396
	Diet group:Infant age	3.736684852	0.154379345	0.454746545
Streptococcaceae				
	Diet group	1.21738651	0.269874161	0.614815315
	Infant age	23.63547729	7.37E-06	9.07E-05
	Delivery mode	0.235633065	0.627378091	0.941067137

	Diet group:Infant age	2.348647681	0.309027861	0.628024363
Clostridiaceae				
	Diet group	7.709303396	0.005493695	0.02662329
	Infant age	6.274283929	0.043406679	0.151923375
	Diet group:Infant age	21.32901407	2.34E-05	0.000183956
Eubacteriaceae				
	Diet group	0.048089509	0.826421656	0.999984565
	Infant age	111.7527423	5.41E-25	3.41E-23
	Delivery mode	4.565146983	0.032628874	0.120918769
	Diet group:Infant age	34.87688928	2.67E-08	5.61E-07
Lachnospiraceae				
	Diet group	0.443170128	0.505596325	0.877021418
	Infant age	0.654247683	0.720994449	0.999984565
	Gestational weight gain	10.78505251	0.00102323	0.006446349
	Diet group:Infant age	0.195202589	0.907010462	0.999984565
Ruminococcaceae				
	Diet group	0.094798996	0.758162645	0.999984565
	Infant age	25.88518823	2.39E-06	3.77E-05
	Sex	0.324617148	0.568846031	0.918905127
	Diet group:Infant age	6.003502769	0.049699948	0.164794566
Erysipelotrichaceae				
	Diet group	0.018252832	0.892530406	0.999984565
	Infant age	0.054211144	0.973258487	0.999984565
	Sex	0.096380067	0.756217718	0.999984565
	Diet group:Infant age	0.046593378	0.976972584	0.999984565
Veillonellaceae				
	Diet group	4.585242411	0.032248409	0.120918769
	Infant age	3.680214666	0.158800381	0.454746545
	Diet group:Infant age	9.186460551	0.010120115	0.042504482
Enterobacteriaceae				
	Diet group	0.907898115	0.340672727	0.67069943
	Infant age	1.056032201	0.589773862	0.928893833
	Diet group:Infant age	1.324058916	0.51580347	0.877021418
Pasteurellaceae				
	Diet group	0.006049953	0.938001905	0.999984565
	Infant age	3.045384379	0.218123865	0.568907576
	Diet group:Infant age	1.283187346	0.526452762	0.877021418
Verrucomicrobiaceae				
	Diet group	0.014545481	0.904004188	0.999984565
	Infant age	22.36925649	1.39E-05	0.000124974
	Sex	19.79116026	8.64E-06	9.07E-05
	Diet group:Infant age	0.739630769	0.690861863	0.989188576

Supplementary Table 6. Infant species-level results for the negative binomial regression models.

	comparison	chisq	pval	adjpval
Bifidobacterium_adolescentis				
	Diet group	0.012255689	0.911849953	0.999878978
	Infant age	6.635900455	0.036227013	0.150782702
	Breastfeeding status	3.147136007	0.207304199	0.514916882
	Diet group:Infant age	2.445207955	0.294462394	0.616328435
Bifidobacterium_bifidum				
	Diet group	0.031656309	0.858784077	0.999878978
	Infant age	7.122901462	0.028397598	0.124949429
	Diet group:Infant age	0.052214338	0.974230677	0.999878978
Bifidobacterium_breve				
	Diet group	1.719244694	0.189790298	0.479142721
	Infant age	12.32703232	0.002104839	0.017060276
	Diet group:Infant age	5.319104984	0.069979531	0.250624367
Bifidobacterium_dentium				
	Diet group	0.143221204	0.705099469	0.969031683
	Infant age	4.420968031	0.109647565	0.337714499
	Diet group:Infant age	2.905210466	0.233959973	0.545906604
Bifidobacterium_longum				
	Diet group	4.026280063	0.044796613	0.176889189
	Infant age	12.43048591	0.001998731	0.017060276
	Gestational weight gain	0.779760518	0.377214392	0.667712832
	Sex	2.430997917	0.118957149	0.352296172
	Diet group:Infant age	11.71266691	0.002861717	0.022035222
Collinsella_aerofaciens				
	Diet group	1.061116867	0.302960592	0.622079082
	Infant age	13.36965685	0.001249729	0.011321076
	Diet group:Infant age	0.678254595	0.712391758	0.969031683
Bacteroides_caccae				
	Diet group	0.070769133	0.790220198	0.992015794
	Infant age	0.118816946	0.942321777	0.999878978
	Delivery mode	1.292582721	0.255572533	0.582013271
	Diet group:Infant age	0.840788333	0.656787885	0.93937951
Bacteroides_dorei				
	Diet group	1.270133108	0.25974171	0.582013271
	Infant age	1.625140329	0.443716176	0.719287275
	Diet group:Infant age	0.403148605	0.817442837	0.999878978
Bacteroides_fragilis				
	Diet group	0.780902437	0.376865285	0.667712832
	Infant age	5.607211134	0.060591203	0.222167743

	Diet group:Infant age	3.0097593	0.222044015	0.526073513
Bacteroides_ovatus				
	Diet group	0.915880702	0.338558895	0.64211721
	Infant age	24.52402859	4.73E-06	8.23E-05
	Diet group:Infant age	11.07865354	0.003929171	0.027504198
Bacteroides_stercoris				
	Diet group	1.016726444	0.313296766	0.626593533
	Infant age	3.937729973	0.139615231	0.398161956
	Diet group:Infant age	3.428502904	0.180098483	0.469163541
Bacteroides_thetaiotaomicron				
	Diet group	0.11606833	0.733338455	0.972345818
	Infant age	11.37363576	0.003390364	0.024862672
	Diet group:Infant age	5.933818928	0.05146211	0.193296707
Bacteroides_uniformis				
	Diet group	0.011417084	0.914907381	0.999878978
	Infant age	4.303604488	0.116274414	0.351103132
	Breastfeeding status	0.834714185	0.65878563	0.93937951
	Diet group:Infant age	2.641097963	0.26698869	0.582013271
Bacteroides_vulgatus				
	Diet group	1.97E-05	0.996457327	0.999878978
	Infant age	0.000242059	0.999878978	0.999878978
	Breastfeeding status	0.000508365	0.99974585	0.999878978
	Diet group:Infant age	0.000416777	0.999791633	0.999878978
Staphylococcus_epidermidis				
	Diet group	0.111245782	0.738730265	0.972345818
	Infant age	105.6343968	1.15E-23	1.78E-21
	Breastfeeding status	24.48985708	4.81E-06	8.23E-05
	Diet group:Infant age	1.268734022	0.530271038	0.809828012
Enterococcus_faecalis				
	Diet group	15.6215729	7.74E-05	0.000992875
	Infant age	3.046208666	0.218033985	0.524644277
	Gestational weight gain	16.0319104	6.23E-05	0.000871974
	Diet group:Infant age	6.280899018	0.043263346	0.175330403
Streptococcus_anginosus				
	Diet group	80.2922536	3.23E-19	2.49E-17
	Infant age	73.33358873	1.19E-16	6.11E-15
	Gestational weight gain	0.950053456	0.329705691	0.64211721
	Delivery mode	1.774785116	0.18279099	0.469163541
	Breastfeeding status	26.81295387	1.51E-06	3.31E-05
	Sex	6.329058874	0.011877533	0.061738954
	Diet group:Infant age	41.88339722	8.04E-10	2.06E-08

Streptococcus_parasanguinis				
	Diet group	0.279026113	0.597340248	0.893110662
	Infant age	4.673378455	0.096647086	0.303747983
	Diet group:Infant age	0.65544351	0.720563485	0.969031683
Streptococcus_salivarius				
	Diet group	2.028656534	0.154357002	0.430711065
	Infant age	4.952898931	0.084041087	0.275368669
	Gestational weight gain	8.062141092	0.004519974	0.030264173
	Diet group:Infant age	0.380330463	0.826822506	0.999878978
Streptococcus_vestibularis				
	Diet group	0.424584196	0.514658438	0.800579792
	Infant age	17.16272216	0.00018757	0.002063265
	Gestational weight gain	3.224408954	0.072547982	0.253917935
	Diet group:Infant age	0.529475793	0.767407064	0.984839065
Clostridium_perfringens				
	Diet group	19.51980025	9.96E-06	0.000153326
	Infant age	8.319947508	0.015607968	0.075113344
	Delivery mode	41.81841353	1.00E-10	3.86E-09
	Diet group:Infant age	43.05561155	4.47E-10	1.38E-08
Eubacterium_rectale				
	Diet group	0.153478186	0.695233394	0.969031683
	Infant age	8.552800492	0.013892582	0.069014762
	Diet group:Infant age	1.693241877	0.428861636	0.714601411
Ruminococcus_gnavus				
	Diet group	1.039879667	0.30784946	0.623800221
	Infant age	0.165290776	0.920677573	0.999878978
	Gestational weight gain	13.68143019	0.000216586	0.002223612
	Diet group:Infant age	0.106876304	0.947964569	0.999878978
Ruminococcus_torques				
	Diet group	0.863989758	0.35262459	0.654260358
	Infant age	1.858193277	0.394910296	0.688972515
	Diet group:Infant age	0.228319993	0.892115209	0.999878978
Faecalibacterium_prausnitzii				
	Diet group	0.0083091	0.927370014	0.999878978
	Infant age	9.474608063	0.008762237	0.051899404
	Diet group:Infant age	1.490423375	0.474633815	0.753542346
*Subdoligranulum*_unclassified				
	Diet group	0.009138886	0.923840236	0.999878978
	Infant age	17.38585261	0.000167768	0.00198741
	Diet group:Infant age	1.596149856	0.450194788	0.722187472

*Coprobacillus*_unclassified				
	Diet group	0.003684343	0.951599095	0.999878978
	Infant age	0.058015617	0.971408879	0.999878978
	Delivery mode	0.014668392	0.903601422	0.999878978
	Diet group:Infant age	0.005512711	0.99724744	0.999878978
Veillonella_atypica				
	Diet group	0.998060437	0.317780281	0.62741235
	Infant age	9.167516556	0.010216428	0.058271478
	Diet group:Infant age	5.098521339	0.078139415	0.265191132
Veillonella_dispar				
	Diet group	0.126390183	0.722204581	0.969031683
	Infant age	8.860225987	0.011913143	0.061738954
	Diet group:Infant age	1.764997707	0.413747724	0.707968328
Veillonella_parvula				
	Diet group	13.2581192	0.000271402	0.002612242
	Infant age	4.719574338	0.094440321	0.30299603
	Diet group:Infant age	10.40306449	0.005508118	0.035196807
*Veillonella*_unclassified				
	Diet group	0.039053306	0.843343095	0.999878978
	Infant age	5.071231331	0.079212936	0.265191132
	Diet group:Infant age	10.3297534	0.005713767	0.035196807
Citrobacter_freundii				
	Diet group	0.145097914	0.703264689	0.969031683
	Infant age	0.465568994	0.792324303	0.992015794
	Gestational weight gain	0.001745181	0.966677777	0.999878978
	Delivery mode	0.217863205	0.640672895	0.930788922
	Breastfeeding status	0.069505839	0.965844028	0.999878978
	Sex	0.016939876	0.896445159	0.999878978
	Diet group:Infant age	0.400901239	0.8183619	0.999878978
*Citrobacter*_unclassified				
	Diet group	0.591608852	0.441797659	0.719287275
	Infant age	0.477126608	0.787758818	0.992015794
	Gestational weight gain	0.001616962	0.967924533	0.999878978
	Diet group:Infant age	0.414495274	0.812818341	0.999878978
Enterobacter_cloacae				
	Diet group	0.63978373	0.423789122	0.714601411
	Infant age	6.873196037	0.032173955	0.137633028
	Diet group:Infant age	8.841190861	0.012027069	0.061738954
Escherichia_coli				
	Diet group	0.848880308	0.356869286	0.654260358
	Infant age	0.975116315	0.614124159	0.905723657

	Breastfeeding status	2.146435554	0.341906566	0.64211721
	Diet group:Infant age	1.680766936	0.431545008	0.714601411
*Escherichia*_unclassified				
	Diet group	0.036118148	0.849271711	0.999878978
	Infant age	2.031126831	0.362198304	0.656218104
	Diet group:Infant age	2.433725586	0.29615782	0.616328435
Klebsiella_oxytoca				
	Diet group	0.319649421	0.571818406	0.863333673
	Infant age	2.499638681	0.286556561	0.612912645
	Gestational weight gain	0.025360296	0.873472572	0.999878978
	Delivery mode	3.834991439	0.050193247	0.193244003
	Breastfeeding status	0.080114022	0.960734665	0.999878978
	Sex	2.006539632	0.156622205	0.430711065
	Diet group:Infant age	3.961875126	0.137939849	0.398161956
Klebsiella_pneumoniae				
	Diet group	0.095286173	0.757561471	0.984227671
	Infant age	3.062909684	0.216220871	0.524644277
	Diet group:Infant age	0.964026572	0.617538857	0.905723657
*Klebsiella*_unclassified				
	Diet group	1.972130026	0.160221953	0.432880365
	Infant age	3.575251951	0.167357008	0.444361711
	Diet group:Infant age	7.195710641	0.027382386	0.124026101
Raoultella_ornithinolytica				
	Diet group	0.7138386	0.398172428	0.688972515
	Infant age	2.631069509	0.268330794	0.582013271
	Diet group:Infant age	1.26553094	0.531120969	0.809828012
Haemophilus_parainfluenzae				
	Diet group	0.092886168	0.760539564	0.984227671
	Infant age	8.192221245	0.016637258	0.077640539
	Delivery mode	0.457009813	0.499024767	0.784181776
	Diet group:Infant age	2.669364452	0.263241814	0.582013271
Akkermansia_muciniphila				
	Diet group	0.125043451	0.723627555	0.969031683
	Infant age	2.183552094	0.335619887	0.64211721
	Diet group:Infant age	0.067240075	0.966938835	0.999878978

Supplementary Table 7. Infant protein gene annotation results.

comparison	chisq	pval	adjpval
ABC transporters			
Diet group	1.631587745	0.201483582	0.429187629
Infant age	6.418935201	0.040378105	0.333662958
Diet group:Infant age	3.798655416	0.149669207	0.383103896
Acarbose and validamycin biosynthesis			
Diet group	0.536535854	0.463871937	0.648378303
Infant age	1.235659712	0.539113122	0.713469202
Diet group:Infant age	0.1471577	0.929062886	0.95652343
Alanine, aspartate and glutamate metabolism			
Diet group	3.053068688	0.080584748	0.343852775
Infant age	2.37134868	0.305540072	0.517836308
Diet group:Infant age	4.013649545	0.134414795	0.371582234
alpha-Linolenic acid metabolism			
Diet group	2.393158719	0.121867164	0.359777503
Infant age	3.023466064	0.220527465	0.440324269
Diet group:Infant age	4.446925179	0.10823369	0.356197648
Amino acid metabolism			
Diet group	5.047106828	0.024667093	0.329254136
Infant age	7.862973792	0.019614486	0.329254136
Diet group:Infant age	7.36742958	0.025129451	0.329254136
Amino acid related enzymes			
Diet group	1.163190574	0.280804825	0.497023804
Infant age	1.009020146	0.603801322	0.748054435
Diet group:Infant age	4.56328182	0.102116505	0.344354957
Amino sugar and nucleotide sugar metabolism			
Diet group	3.211703259	0.073113336	0.343852775
Infant age	0.533106548	0.766015194	0.861958863
Diet group:Infant age	1.657385556	0.436619672	0.630583005
Aminoacyl-tRNA biosynthesis			
Diet group	1.273408367	0.259128245	0.472662078
Infant age	1.070361152	0.585563542	0.739856806
Diet group:Infant age	3.634657964	0.162459103	0.390982586
Aminobenzoate degradation			
Diet group	0.28944692	0.590575175	0.74060032
Infant age	1.223693018	0.542348492	0.714705005
Diet group:Infant age	1.486401173	0.475589312	0.656257087
Antimicrobial resistance genes			
Diet group	1.852275327	0.173518992	0.395019013
Infant age	14.49792201	0.000710913	0.147395888
Diet group:Infant age	9.135163767	0.010383037	0.329254136

Arachidonic acid metabolism			
Diet group	3.989969789	0.045771885	0.337039819
Infant age	9.241718966	0.009844331	0.329254136
Diet group:Infant age	8.234427036	0.016289843	0.329254136
Arginine and proline metabolism			
Diet group	0.398141829	0.528050457	0.706338461
Infant age	0.013937696	0.993055378	0.996561944
Diet group:Infant age	3.682738039	0.158600151	0.386859975
Arginine biosynthesis			
Diet group	3.650969531	0.056036582	0.337039819
Infant age	1.728367561	0.421395364	0.618179047
Diet group:Infant age	1.408205725	0.494552056	0.676068965
Ascorbate and aldarate metabolism			
Diet group	2.507517522	0.113304291	0.359777503
Infant age	5.883636089	0.052769704	0.337039819
Diet group:Infant age	6.72965663	0.03456795	0.329254136
Atrazine degradation			
Diet group	0.059480562	0.807318824	0.890888637
Infant age	8.605720226	0.013529807	0.329254136
Diet group:Infant age	8.520726165	0.014117176	0.329254136
Bacterial chemotaxis			
Diet group	1.762187514	0.184351898	0.412425514
Infant age	6.120599571	0.046873641	0.337039819
Diet group:Infant age	4.311008466	0.115844763	0.359777503
Bacterial invasion of epithelial cells			
Diet group	0.456454974	0.499285423	0.676794509
Infant age	5.388003439	0.067609842	0.342608231
Diet group:Infant age	4.684170713	0.09612697	0.344354957
Bacterial motility proteins			
Diet group	0.107908419	0.742538053	0.853712881
Infant age	4.316078312	0.115551477	0.359777503
Diet group:Infant age	1.638643581	0.440730461	0.633104727
Bacterial secretion system			
Diet group	2.030347551	0.154185345	0.385747068
Infant age	1.803353593	0.405888496	0.604342885
Diet group:Infant age	1.986871872	0.370302168	0.571533371
Bacterial toxins			
Diet group	2.129499454	0.144487932	0.379703033
Infant age	2.558989691	0.278177788	0.494361668
Diet group:Infant age	1.128855729	0.568685419	0.73045832
Base excision repair			
Diet group	1.401133355	0.236533902	0.452692809
Infant age	0.187633735	0.91044948	0.946989258

Diet group:Infant age	3.157811571	0.206200602	0.433051682
Benzoate degradation			
Diet group	7.513845245	0.006122652	0.329254136
Infant age	2.202876206	0.332392725	0.537268239
Diet group:Infant age	4.598929683	0.100312512	0.344354957
beta-Alanine metabolism			
Diet group	2.919474685	0.087516351	0.344354957
Infant age	4.953624306	0.084010612	0.343852775
Diet group:Infant age	4.141429031	0.126095652	0.359777503
beta-Lactam resistance			
Diet group	0.581190341	0.445846123	0.637195662
Infant age	9.344364077	0.009351841	0.329254136
Diet group:Infant age	2.531178789	0.282072995	0.497023804
Betalain biosynthesis			
Diet group	4.994100222	0.025433874	0.329254136
Infant age	1.476041209	0.478059248	0.656408062
Diet group:Infant age	4.291995501	0.116951291	0.359777503
Biofilm formation - Escherichia coli			
Diet group	0.91140669	0.339741458	0.543236984
Infant age	4.91486781	0.085654467	0.343852775
Diet group:Infant age	4.903846577	0.086127779	0.343852775
Biofilm formation - Pseudomonas aeruginosa			
Diet group	0.070792574	0.790186271	0.882398313
Infant age	0.684828413	0.710054035	0.83488395
Diet group:Infant age	1.663328335	0.435324231	0.630583005
Biofilm formation - Vibrio cholerae			
Diet group	1.699294921	0.192380212	0.416935511
Infant age	2.88252887	0.236628368	0.452692809
Diet group:Infant age	4.16379062	0.124693655	0.359777503
Biosynthesis and biodegradation of secondary metabolites			
Diet group	4.716046334	0.029882375	0.329254136
Infant age	3.367700303	0.185657788	0.412425514
Diet group:Infant age	5.788859999	0.055330555	0.337039819
Biosynthesis of ansamycins			
Diet group	2.114614861	0.145898754	0.379703033
Infant age	2.300900449	0.316494244	0.528177462
Diet group:Infant age	2.491406075	0.287738546	0.501325982
Biosynthesis of siderophore group nonribosomal peptides			
Diet group	0.81912677	0.365435602	0.568131936
Infant age	6.928632122	0.031294402	0.329254136
Diet group:Infant age	3.530857436	0.171113411	0.393340256
Biosynthesis of unsaturated fatty acids			
Diet group	2.037406985	0.153471066	0.385747068

Infant age	5.231529353	0.07311186	0.343852775
Diet group:Infant age	5.062726994	0.079550479	0.343852775
Biosynthesis of vancomycin group antibiotics			
Diet group	1.90727754	0.167265988	0.393340256
Infant age	7.171605264	0.027714414	0.329254136
Diet group:Infant age	1.675169246	0.432754527	0.630382473
Biotin metabolism			
Diet group	5.963900922	0.014601699	0.329254136
Infant age	6.82215007	0.033005699	0.329254136
Diet group:Infant age	7.322599564	0.025699088	0.329254136
Butanoate metabolism			
Diet group	1.339855986	0.247059367	0.461474253
Infant age	3.382707142	0.184269933	0.412425514
Diet group:Infant age	3.513686087	0.17258886	0.394670114
C5-Branched dibasic acid metabolism			
Diet group	0.536846303	0.463742667	0.648378303
Infant age	0.420722122	0.810291629	0.890888637
Diet group:Infant age	1.783550247	0.409927435	0.605641008
Caprolactam degradation			
Diet group	0.658852774	0.416965249	0.613126206
Infant age	3.582999962	0.16670992	0.393340256
Diet group:Infant age	5.80476709	0.054892226	0.337039819
Carbapenem biosynthesis			
Diet group	2.702424078	0.1001958	0.344354957
Infant age	1.938527306	0.379362278	0.578604268
Diet group:Infant age	3.879804896	0.143717969	0.379703033
Carbohydrate digestion and absorption			
Diet group	0.988401872	0.320133285	0.528177462
Infant age	3.092145719	0.21308314	0.440324269
Diet group:Infant age	5.116430416	0.077442837	0.343852775
Carbohydrate metabolism			
Diet group	1.105089447	0.29315189	0.503702972
Infant age	0.447353733	0.799573462	0.886514605
Diet group:Infant age	1.143791798	0.564454275	0.73045832
Carbon fixation pathways in prokaryotes			
Diet group	4.257379429	0.039080164	0.333662958
Infant age	4.952384798	0.084062694	0.343852775
Diet group:Infant age	6.976756627	0.030550375	0.329254136
Cationic antimicrobial peptide (CAMP) resistance			
Diet group	2.345275984	0.125663304	0.359777503
Infant age	8.096436529	0.017453444	0.329254136
Diet group:Infant age	6.742275226	0.034350537	0.329254136
Cell growth			

Diet group	1.005884609	0.315890782	0.528177462
Infant age	1.710069677	0.425268375	0.622392775
Diet group:Infant age	1.067459072	0.586413835	0.739856806
Cell motility and secretion			
Diet group	0.113588309	0.736095305	0.850902029
Infant age	5.025602234	0.081040916	0.343852775
Diet group:Infant age	4.218136732	0.121350968	0.359777503
Chaperones and folding catalysts			
Diet group	0.139842961	0.70843718	0.834560466
Infant age	2.196883107	0.33339025	0.537268239
Diet group:Infant age	2.503874232	0.285950341	0.501017217
Chloroalkane and chloroalkene degradation			
Diet group	0.036503184	0.848480101	0.90523949
Infant age	3.117104902	0.210440474	0.43777249
Diet group:Infant age	0.765151377	0.682102262	0.811219134
Chlorocyclohexane and chlorobenzene degradation			
Diet group	3.566485547	0.058956965	0.337039819
Infant age	6.335639485	0.042095276	0.333662958
Diet group:Infant age	3.527803045	0.171374935	0.393340256
Cholesterol metabolism			
Diet group	0.308848067	0.578387919	0.738721326
Infant age	1.819808209	0.402562826	0.604342885
Diet group:Infant age	0.55309796	0.758396472	0.861344571
Chromosome and associated proteins			
Diet group	0.259147018	0.610706771	0.750814139
Infant age	2.466849806	0.291293216	0.502242444
Diet group:Infant age	2.13072248	0.344603348	0.548555062
Citrate cycle (TCA cycle)			
Diet group	1.705107948	0.191621322	0.416916929
Infant age	2.465468907	0.291494409	0.502242444
Diet group:Infant age	3.313477311	0.190760101	0.416916929
Cyanoamino acid metabolism			
Diet group	0.323403197	0.56956959	0.73045832
Infant age	3.060908125	0.216437369	0.440324269
Diet group:Infant age	1.388684237	0.499402888	0.676794509
Cysteine and methionine metabolism			
Diet group	0.335768127	0.562282184	0.730145133
Infant age	0.534473778	0.765491713	0.861958863
Diet group:Infant age	4.970009071	0.083325177	0.343852775
Cytoskeleton proteins			
Diet group	0.24357239	0.621637769	0.761139158
Infant age	0.248840709	0.883008586	0.923739214
Diet group:Infant age	1.135334826	0.566846116	0.73045832

D-Alanine metabolism			
Diet group	0.088833359	0.76566557	0.861958863
Infant age	0.982052465	0.61199802	0.750814139
Diet group:Infant age	1.127449283	0.569085472	0.73045832
D-Arginine and D-ornithine metabolism			
Diet group	2.524881938	0.112063168	0.359777503
Infant age	2.253699318	0.324052526	0.533229289
Breastfeeding status	12.00063886	0.002477961	0.268637448
Diet group:Infant age	2.049081182	0.358961338	0.562402902
D-Glutamine and D-glutamate metabolism			
Diet group	0.267612398	0.604937911	0.748054435
Infant age	0.009721267	0.99515116	0.996753658
Diet group:Infant age	6.69667838	0.035142671	0.329254136
Dioxin degradation			
Diet group	2.400922094	0.121263754	0.359777503
Infant age	1.270928079	0.529689634	0.707010628
Diet group:Infant age	3.070419778	0.215410474	0.440324269
DNA repair and recombination proteins			
Diet group	0.456136428	0.499435176	0.676794509
Infant age	0.132047195	0.936108774	0.959241611
Diet group:Infant age	3.994647348	0.13569797	0.371829466
DNA replication proteins			
Diet group	0.936010021	0.333306079	0.537268239
Infant age	0.339337298	0.843944412	0.90349987
Diet group:Infant age	4.527775666	0.103945575	0.347602945
Energy metabolism			
Diet group	0.5582912	0.454949565	0.6427644
Infant age	2.691587695	0.260332961	0.473409196
Diet group:Infant age	2.644377928	0.266551192	0.479175842
Ether lipid metabolism			
Diet group	0.57652904	0.447676052	0.637195662
Infant age	1.231396013	0.540263656	0.713469202
Diet group:Infant age	0.640947654	0.72580505	0.848591618
Ethylbenzene degradation			
Diet group	2.730372929	0.098456292	0.344354957
Infant age	2.282225311	0.319463371	0.528177462
Diet group:Infant age	4.353792049	0.113392954	0.359777503
Exosome			
Diet group	3.50227337	0.061284648	0.340348671
Infant age	3.819383353	0.14812605	0.380720675
Diet group:Infant age	4.488632182	0.10600001	0.350720143
Fatty acid biosynthesis			
Diet group	4.564496478	0.032641268	0.329254136

Infant age	10.77372089	0.004576318	0.329254136
Diet group:Infant age	3.729752886	0.15491535	0.385747068
Fatty acid degradation			
Diet group	0.045254847	0.83153611	0.895107118
Infant age	0.601398221	0.740300488	0.852716488
Diet group:Infant age	1.06859604	0.586080563	0.739856806
Fatty acid elongation			
Diet group	0.10298225	0.74827975	0.857145496
Infant age	5.927687498	0.051620121	0.337039819
Diet group:Infant age	2.221054845	0.32938519	0.537268239
Flagellar assembly			
Diet group	0.008000778	0.928726616	0.95652343
Infant age	2.868644216	0.238276835	0.452692809
Diet group:Infant age	0.385444953	0.824710819	0.892121964
Flavone and flavonol biosynthesis			
Diet group	0.007691691	0.930113303	0.95652343
Infant age	7.016237455	0.029953212	0.329254136
Diet group:Infant age	1.460673933	0.48174663	0.660014106
Flavonoid biosynthesis			
Diet group	1.704493646	0.191701353	0.416916929
Infant age	2.97229598	0.226242467	0.442524575
Diet group:Infant age	2.031241105	0.36217761	0.564597678
Fluorobenzoate degradation			
Diet group	3.99327436	0.045682208	0.337039819
Infant age	1.913256345	0.384186114	0.582838446
Diet group:Infant age	1.261057421	0.532310288	0.70844918
Folate biosynthesis			
Diet group	2.386880786	0.122357553	0.359777503
Infant age	3.959828615	0.138081069	0.375233027
Diet group:Infant age	3.908488182	0.141671527	0.379703033
Fructose and mannose metabolism			
Diet group	2.014809935	0.155770775	0.386013635
Infant age	7.754365507	0.020709086	0.329254136
Diet group:Infant age	5.174807342	0.07521507	0.343852775
Galactose metabolism			
Diet group	6.541036932	0.010541389	0.329254136
Infant age	6.453268566	0.039690862	0.333662958
Diet group:Infant age	7.74394194	0.020817299	0.329254136
Geraniol degradation			
Diet group	1.874226251	0.170991826	0.393340256
Infant age	2.5148355	0.284387439	0.499686403
Diet group:Infant age	5.129440257	0.076940712	0.343852775
Glucagon signaling pathway			

Diet group	3.21320646	0.073046204	0.343852775
Infant age	4.849618641	0.088494992	0.344354957
Diet group:Infant age	2.328436289	0.312166636	0.526199587
Glucosinolate biosynthesis			
Diet group	3.061187344	0.080183058	0.343852775
Infant age	5.448268596	0.065602971	0.342608231
Diet group:Infant age	5.448099762	0.065608509	0.342608231
Glutathione metabolism			
Diet group	0.55638113	0.455722026	0.6427644
Infant age	3.948110167	0.138892492	0.375613609
Diet group:Infant age	4.026836504	0.133531449	0.370788221
Glycan biosynthesis and metabolism			
Diet group	2.502711597	0.113650465	0.359777503
Infant age	3.020456863	0.220859521	0.440324269
Diet group:Infant age	1.162591999	0.559173209	0.727627062
Glycerolipid metabolism			
Diet group	0.025666639	0.872717141	0.922872944
Infant age	2.282376439	0.319439232	0.528177462
Diet group:Infant age	0.796347214	0.671545433	0.803271652
Glycerophospholipid metabolism			
Diet group	1.388142501	0.238719037	0.452692809
Infant age	5.936228081	0.051400158	0.337039819
Diet group:Infant age	5.37539924	0.068037271	0.342608231
Glycine, serine and threonine metabolism			
Diet group	0.033216059	0.855384391	0.909485626
Infant age	0.532259646	0.766339633	0.861958863
Diet group:Infant age	2.737852368	0.25437997	0.470118095
Glycolysis / Gluconeogenesis			
Diet group	0.000479976	0.982521058	0.992091068
Infant age	1.980352951	0.371511123	0.571979996
Diet group:Infant age	2.493685482	0.287410796	0.501325982
Glycosaminoglycan binding proteins			
Diet group	0.056663163	0.811849873	0.890888637
Infant age	0.179501159	0.914159167	0.948118156
Diet group:Infant age	2.963723671	0.227214259	0.443032191
Glycosaminoglycan degradation			
Diet group	2.226056307	0.135699821	0.371829466
Infant age	2.719448893	0.25673151	0.470118095
Diet group:Infant age	1.209104096	0.546319097	0.716533921
Glycosylphosphatidylinositol (GPI)-anchored proteins			
Diet group	2.006373413	0.156639372	0.386625751
Infant age	0.390620294	0.822579498	0.891366634
Diet group:Infant age	1.662449136	0.435515641	0.630583005

Glycosyltransferases			
Diet group	4.228044855	0.039761218	0.333662958
Infant age	9.516383096	0.008581114	0.329254136
Diet group:Infant age	4.591594298	0.100681103	0.344354957
Glyoxylate and dicarboxylate metabolism			
Diet group	1.246226156	0.264274487	0.47646009
Infant age	2.184609098	0.335442558	0.539135068
Diet group:Infant age	2.671576972	0.262950761	0.475451666
Histidine metabolism			
Diet group	0.124491002	0.724213774	0.848325739
Infant age	4.702680822	0.095241414	0.344354957
Diet group:Infant age	9.388345139	0.009148434	0.329254136
Homologous recombination			
Diet group	0.805016142	0.369597844	0.571533371
Infant age	0.406733182	0.815979056	0.890888637
Diet group:Infant age	5.854098294	0.053554838	0.337039819
Inorganic ion transport and metabolism			
Diet group	0.596851714	0.439781767	0.633104727
Infant age	2.289688609	0.318273468	0.528177462
Diet group:Infant age	2.38038423	0.304162824	0.516910592
Inositol phosphate metabolism			
Diet group	0.306115184	0.580073905	0.739356494
Infant age	8.63027246	0.013364729	0.329254136
Diet group:Infant age	5.125282576	0.077100826	0.343852775
Isoquinoline alkaloid biosynthesis			
Diet group	4.130545475	0.042115773	0.333662958
Infant age	6.727837898	0.0345994	0.329254136
Diet group:Infant age	4.888471959	0.086792421	0.343852775
Limonene and pinene degradation			
Diet group	0.658852774	0.416965249	0.613126206
Infant age	3.582999962	0.16670992	0.393340256
Diet group:Infant age	5.80476709	0.054892226	0.337039819
Linoleic acid metabolism			
Diet group	1.952224154	0.162347378	0.390982586
Infant age	3.856850636	0.145376941	0.379703033
Diet group:Infant age	4.428339096	0.109244199	0.357631008
Lipid biosynthesis proteins			
Diet group	2.348034393	0.125441089	0.359777503
Infant age	5.45314176	0.065443318	0.342608231
Diet group:Infant age	2.762101507	0.251314345	0.468016534
Lipid metabolism			
Diet group	0.044337621	0.833226723	0.895107118
Infant age	2.008760542	0.366271554	0.568131936

Diet group:Infant age	0.924334981	0.629916826	0.766748074
Lipoic acid metabolism			
Diet group	0.54626315	0.459848602	0.645656502
Infant age	1.679029577	0.431920045	0.630382473
Diet group:Infant age	0.102393944	0.950091511	0.969921912
Lipopolysaccharide biosynthesis			
Diet group	3.819842259	0.050649006	0.337039819
Infant age	2.940928295	0.229818791	0.443935676
Diet group:Infant age	3.037602039	0.218974276	0.440324269
Lipopolysaccharide biosynthesis proteins			
Diet group	3.850898005	0.049719364	0.337039819
Infant age	4.894703627	0.086522411	0.343852775
Diet group:Infant age	4.488529637	0.106005445	0.350720143
Lysine biosynthesis			
Diet group	1.374347846	0.241066266	0.45302916
Infant age	3.560106251	0.168629189	0.393340256
Diet group:Infant age	6.751928035	0.034185147	0.329254136
Lysine degradation			
Diet group	2.021919595	0.155043034	0.385747068
Infant age	4.651334852	0.097718203	0.344354957
Diet group:Infant age	5.03308062	0.080738454	0.343852775
Lysosome			
Diet group	4.406420515	0.035803894	0.329254136
Infant age	3.371329796	0.185321171	0.412425514
Diet group:Infant age	1.169884888	0.557137928	0.726498513
Mannose type O-glycan biosyntheis			
Diet group	4.397311707	0.035995629	0.329254136
Infant age	7.099069416	0.028738008	0.329254136
Breastfeeding status	16.25855256	0.000294781	0.091677035
Diet group:Infant age	3.856548511	0.145398903	0.379703033
Membrane and intracellular structural molecules			
Diet group	2.198462221	0.138148494	0.375233027
Infant age	2.099695287	0.349991068	0.552523971
Diet group:Infant age	4.066923423	0.130881661	0.36506006
Membrane trafficking			
Diet group	2.954569279	0.085635121	0.343852775
Infant age	2.872276991	0.237844425	0.452692809
Diet group:Infant age	1.258297497	0.533045363	0.70844918
Messenger RNA Biogenesis			
Diet group	2.861216514	0.090738851	0.344354957
Infant age	0.566533653	0.753318756	0.861331371
Diet group:Infant age	2.941690266	0.22973125	0.443935676
Metabolism of cofactors and vitamins			

Diet group	0.570887991	0.449906268	0.638907988
Infant age	3.085224443	0.213821821	0.440324269
Diet group:Infant age	0.909757211	0.634524991	0.769346091
Metabolism of xenobiotics by cytochrome P450			
Diet group	0.691070795	0.405800402	0.604342885
Infant age	1.485350839	0.475839142	0.656257087
Diet group:Infant age	2.834740793	0.242350465	0.454042137
Methane metabolism			
Diet group	0.120450677	0.728546208	0.849335102
Infant age	1.127238927	0.56914533	0.73045832
Diet group:Infant age	3.342913303	0.187973055	0.416082705
Mineral absorption			
Diet group	1.029252647	0.310334264	0.52453237
Infant age	0.879445815	0.644214903	0.775051586
Diet group:Infant age	0.527507292	0.768162756	0.862449882
Mismatch repair			
Diet group	0.520357411	0.470689261	0.653104642
Infant age	0.400068932	0.818702535	0.890888637
Diet group:Infant age	5.202981046	0.074162954	0.343852775
Monobactam biosynthesis			
Diet group	1.123265669	0.289216356	0.502242444
Infant age	2.749907669	0.252851268	0.469473101
Diet group:Infant age	4.658394657	0.097373875	0.344354957
N-Glycan biosynthesis			
Diet group	0.05132614	0.820771551	0.890959694
Infant age	0.709685141	0.701283837	0.827701227
Diet group:Infant age	0.737650834	0.691546132	0.820881096
Naphthalene degradation			
Diet group	0.126822486	0.721749616	0.847034455
Infant age	3.283875968	0.193604476	0.417571927
Diet group:Infant age	0.554343385	0.757924356	0.861344571
Neomycin, kanamycin and gentamicin biosynthesis			
Diet group	4.120026508	0.042378414	0.333662958
Infant age	0.88286023	0.643116033	0.775051586
Diet group:Infant age	1.020913688	0.600221309	0.746675308
Nicotinate and nicotinamide metabolism			
Diet group	0.918796012	0.337791299	0.541510794
Infant age	0.057496812	0.971660898	0.984320975
Diet group:Infant age	0.245060296	0.884679233	0.923739214
Nitrogen metabolism			
Diet group	2.836790357	0.092128071	0.344354957
Infant age	3.887808878	0.14314396	0.379703033
Diet group:Infant age	3.692480146	0.157829479	0.386859975

Nitrotoluene degradation			
Diet group	0.945410678	0.330890227	0.537268239
Infant age	2.395473077	0.301876725	0.516145463
Diet group:Infant age	1.391860943	0.498610289	0.676794509
Nonribosomal peptide structures			
Diet group	1.951956634	0.162376161	0.390982586
Infant age	1.523981356	0.46673638	0.64946315
Diet group:Infant age	1.031746998	0.596978906	0.74413002
Novobiocin biosynthesis			
Diet group	4.485644751	0.034180651	0.329254136
Infant age	9.104048388	0.010545836	0.329254136
Diet group:Infant age	7.238295677	0.026805509	0.329254136
Nucleotide excision repair			
Diet group	2.983379203	0.084123469	0.343852775
Infant age	4.299299009	0.116524992	0.359777503
Diet group:Infant age	4.584987603	0.101014238	0.344354957
Nucleotide metabolism			
Diet group	0.118830646	0.730306225	0.849335102
Infant age	1.047979159	0.592153386	0.741085324
Diet group:Infant age	0.93506112	0.626547581	0.764142344
One carbon pool by folate			
Diet group	1.595312437	0.206568774	0.433051682
Infant age	2.059065528	0.357173806	0.561015423
Diet group:Infant age	8.594743101	0.01360427	0.329254136
Other glycan degradation			
Diet group	2.513788475	0.112854336	0.359777503
Infant age	3.005833294	0.222480316	0.440324269
Diet group:Infant age	1.503870238	0.471453351	0.653104642
Diet group:Infant age	3.856548511	0.145398903	0.379703033
Oxidative phosphorylation			
Diet group	0.686346505	0.407409857	0.604794584
Infant age	2.199362369	0.332977225	0.537268239
Diet group:Infant age	2.475753554	0.289999298	0.502242444
Pantothenate and CoA biosynthesis			
Diet group	0.000186051	0.989117142	0.995519195
Infant age	1.11654839	0.57219571	0.732316321
Diet group:Infant age	1.917230848	0.383423397	0.582838446
Penicillin and cephalosporin biosynthesis			
Diet group	0.199270745	0.655310125	0.786878181
Infant age	0.551850923	0.758869493	0.861344571
Diet group:Infant age	0.004895307	0.99755534	0.99755534
Pentose and glucuronate interconversions			
Diet group	2.299364426	0.12942695	0.364269514

Infant age	5.606005681	0.060627734	0.340348671
Diet group:Infant age	4.20354532	0.122239547	0.359777503
Pentose phosphate pathway			
Diet group	0.346997823	0.555817529	0.726299377
Infant age	0.774611555	0.678883476	0.808937781
Diet group:Infant age	0.25475097	0.880403033	0.923739214
Peptidases			
Diet group	0.292251591	0.588781258	0.740552283
Infant age	2.849514082	0.240566904	0.45302916
Diet group:Infant age	2.321876013	0.313192267	0.526501594
Peptidoglycan biosynthesis and degradation proteins			
Diet group	0.076900581	0.781542434	0.874315457
Infant age	3.180458811	0.203878835	0.431335495
Diet group:Infant age	3.261310665	0.195801217	0.419508628
Peroxisome			
Diet group	2.344816433	0.125700368	0.359777503
Infant age	3.279623748	0.194016538	0.417571927
Diet group:Infant age	3.1871671	0.203196142	0.431335495
Phenazine biosynthesis			
Diet group	0.406924937	0.523534427	0.702799188
Infant age	4.62727551	0.098900819	0.344354957
Diet group:Infant age	6.946936016	0.031009303	0.329254136
Phenylalanine metabolism			
Diet group	0.112463051	0.737357224	0.850902029
Infant age	1.655884972	0.436947387	0.630583005
Diet group:Infant age	0.721944354	0.696998391	0.825777141
Phenylalanine, tyrosine and tryptophan biosynthesis			
Diet group	0.531071958	0.466156534	0.64946315
Infant age	3.022397299	0.220645343	0.440324269
Diet group:Infant age	5.866929504	0.053212351	0.337039819
Phenylpropanoid biosynthesis			
Diet group	0.550650091	0.458052189	0.64458928
Infant age	1.937618856	0.379534632	0.578604268
Diet group:Infant age	0.71072574	0.700919055	0.827701227
Phosphonate and phosphinate metabolism			
Diet group	1.910756515	0.166879248	0.393340256
Infant age	3.548992631	0.169568837	0.393340256
Diet group:Infant age	5.085535932	0.078648402	0.343852775
Phosphotransferase system (PTS)			
Diet group	0.421608384	0.516135581	0.694883834
Infant age	5.367652394	0.068301319	0.342608231
Diet group:Infant age	3.539362954	0.170387252	0.393340256
Polycyclic aromatic hydrocarbon degradation			

Diet group	3.615855119	0.057231293	0.337039819
Infant age	0.77716526	0.678017195	0.808937781
Diet group:Infant age	1.035446369	0.595875704	0.74413002
Polyketide biosynthesis proteins			
Diet group	0.303863059	0.581470704	0.739621223
Infant age	7.748484698	0.020770068	0.329254136
Diet group:Infant age	1.874126426	0.391776712	0.590036598
Polyketide sugar unit biosynthesis			
Diet group	0.091619212	0.762128667	0.861958863
Infant age	0.218847353	0.896350574	0.933886193
Diet group:Infant age	0.280909272	0.868963084	0.922183353
Porphyrin and chlorophyll metabolism			
Diet group	5.077583404	0.024237129	0.329254136
Infant age	5.187948916	0.074722468	0.343852775
Diet group:Infant age	5.146436969	0.076289613	0.343852775
Prenyltransferases			
Diet group	0.037412955	0.846626697	0.904814099
Infant age	0.944350704	0.623644146	0.762095597
Diet group:Infant age	3.839308201	0.146657682	0.380087826
Prodigiosin biosyntheses			
Diet group	3.351800395	0.067131552	0.342608231
Infant age	7.163946103	0.027820752	0.329254136
Diet group:Infant age	4.663253355	0.097137607	0.344354957
Prokaryotic Defense System			
Diet group	1.485000385	0.222993802	0.440324269
Infant age	4.33665283	0.114368863	0.359777503
Diet group:Infant age	4.208535756	0.121934913	0.359777503
Propanoate metabolism			
Diet group	1.429743008	0.231806384	0.446388765
Infant age	4.57964661	0.101284357	0.344354957
Diet group:Infant age	4.959028792	0.083783902	0.343852775
Proteasome			
Diet group	5.189354177	0.02272566	0.329254136
Infant age	11.61436374	0.003005889	0.268637448
Diet group:Infant age	5.498707454	0.063969189	0.342608231
Protein digestion and absorption			
Diet group	1.580642812	0.208668126	0.435542196
Infant age	2.121866336	0.34613266	0.548555062
Diet group:Infant age	4.225568459	0.120900882	0.359777503
Protein export			
Diet group	0.105992071	0.744754075	0.854680876
Infant age	1.205909958	0.547192303	0.716533921
Diet group:Infant age	2.388818775	0.302882788	0.516145463

Protein folding and associated processing			
Diet group	1.263126821	0.261060055	0.473409196
Infant age	3.072499082	0.215186639	0.440324269
Diet group:Infant age	3.789441014	0.150360354	0.383295656
Protein kinases			
Diet group	0.18190876	0.669737988	0.802653234
Infant age	0.377214129	0.828111837	0.894245769
Diet group:Infant age	0.884778462	0.642499506	0.775051586
Protein phosphatase and associated proteins			
Diet group	2.421650401	0.119668793	0.359777503
Infant age	5.557334067	0.062121258	0.341941791
Diet group:Infant age	8.505545576	0.014224737	0.329254136
Purine metabolism			
Diet group	0.217262261	0.641133903	0.775051586
Infant age	0.403666707	0.817231105	0.890888637
Diet group:Infant age	1.969039091	0.373618691	0.573804508
Pyrimidine metabolism			
Diet group	0.002835456	0.957533512	0.973179484
Infant age	1.076233073	0.583846872	0.739856806
Diet group:Infant age	3.575824998	0.167309063	0.393340256
Pyruvate metabolism			
Diet group	3.714549713	0.053940172	0.337039819
Infant age	6.196649321	0.045124738	0.337039819
Diet group:Infant age	6.070958396	0.048051631	0.337039819
Quorum sensing			
Diet group	1.218354304	0.269683862	0.483166119
Infant age	0.144323258	0.930380507	0.95652343
Diet group:Infant age	0.433115518	0.805286021	0.890888637
Replication, recombination and repair proteins			
Diet group	0.096521783	0.756044245	0.861344571
Infant age	0.265026759	0.875891215	0.923047252
Diet group:Infant age	0.147494249	0.928906562	0.95652343
Retinol metabolism			
Diet group	2.302535919	0.129162967	0.364269514
Infant age	7.012034738	0.03001622	0.329254136
Diet group:Infant age	3.824984419	0.147711799	0.380720675
Riboflavin metabolism			
Diet group	1.990178407	0.158322227	0.386859975
Infant age	7.563733302	0.022780129	0.329254136
Diet group:Infant age	6.408506934	0.040589192	0.333662958
Ribosome biogenesis			
Diet group	0.269427411	0.603716129	0.748054435
Infant age	0.989988546	0.609574398	0.750814139

Diet group:Infant age	1.786965318	0.409228066	0.605641008
RIG-I-like receptor signaling pathway			
Diet group	0.118618745	0.730537427	0.849335102
Infant age	0.422116205	0.809727018	0.890888637
Diet group:Infant age	0.138006126	0.933323821	0.957966034
RNA degradation			
Diet group	3.19844613	0.07370827	0.343852775
Infant age	1.057482747	0.58934627	0.740552283
Diet group:Infant age	4.557333137	0.102420687	0.344354957
RNA polymerase			
Diet group	1.286149653	0.256758735	0.470118095
Infant age	0.365035962	0.833169663	0.895107118
Diet group:Infant age	2.244809118	0.325496178	0.534191617
RNA transport			
Diet group	3.179124568	0.07458468	0.343852775
Infant age	11.60284506	0.003023251	0.268637448
Diet group:Infant age	6.94652477	0.03101568	0.329254136
Salmonella infection			
Diet group	1.284965961	0.256977737	0.470118095
Infant age	5.178877319	0.075062164	0.343852775
Diet group:Infant age	5.594955258	0.060963642	0.340348671
Secondary bile acid biosynthesis			
Diet group	0.005913115	0.938705658	0.960320591
Infant age	0.521145793	0.77060998	0.863638572
Diet group:Infant age	1.609052038	0.447299888	0.637195662
Secretion system			
Diet group	0.754255356	0.385132295	0.582852281
Infant age	5.040604179	0.080435304	0.343852775
Diet group:Infant age	1.802144423	0.406133965	0.604342885
Selenocompound metabolism			
Diet group	0.042708194	0.836275703	0.896833599
Infant age	1.233795943	0.539615747	0.713469202
Diet group:Infant age	6.19124282	0.045246887	0.337039819
Signal transduction mechanisms			
Diet group	5.183528764	0.02280197	0.329254136
Infant age	2.947249865	0.22909353	0.443935676
Diet group:Infant age	3.40297522	0.182411964	0.411087832
Sphingolipid metabolism			
Diet group	3.587451586	0.058217456	0.337039819
Infant age	3.256576025	0.19626529	0.419508628
Diet group:Infant age	2.531256408	0.282062048	0.497023804
Sphingolipid signaling pathway			
Diet group	0.067225122	0.795420691	0.886514605

Infant age	0.013329189	0.993357565	0.996561944
Diet group:Infant age	1.626837963	0.443339702	0.635385471
Staphylococcus aureus infection			
Diet group	0.113048499	0.736699802	0.850902029
Infant age	4.995384767	0.082274638	0.343852775
Diet group:Infant age	0.178573169	0.91458343	0.948118156
Starch and sucrose metabolism			
Diet group	1.488634791	0.222428401	0.440324269
Infant age	2.196721036	0.333417267	0.537268239
Diet group:Infant age	0.044995319	0.977753525	0.988882427
Steroid degradation			
Diet group	0.055613323	0.813568645	0.890888637
Infant age	0.982174412	0.611960705	0.750814139
Breastfeeding status	13.04030777	0.001473442	0.229120285
Diet group:Infant age	0.027911267	0.986141295	0.994132715
Stilbenoid, diarylheptanoid and gingerol biosynthesis			
Diet group	1.704493646	0.191701353	0.416916929
Infant age	2.97229598	0.226242467	0.442524575
Diet group:Infant age	2.031241105	0.36217761	0.564597678
Streptomycin biosynthesis			
Diet group	0.869837048	0.351000425	0.552714593
Infant age	0.262397854	0.877043289	0.923047252
Diet group:Infant age	1.33167142	0.513843924	0.694806349
Styrene degradation			
Diet group	0.78812778	0.374666845	0.573996989
Infant age	0.413593978	0.813184719	0.890888637
Diet group:Infant age	1.885090993	0.389634758	0.588234999
Sulfur metabolism			
Diet group	3.409522297	0.064821209	0.342608231
Infant age	4.758507505	0.092619669	0.344354957
Diet group:Infant age	5.823776215	0.054372971	0.337039819
Sulfur relay system			
Diet group	2.487061705	0.114785844	0.359777503
Infant age	5.734332258	0.056859832	0.337039819
Diet group:Infant age	5.658292817	0.059063248	0.337039819
Synthesis and degradation of ketone bodies			
Diet group	4.421369784	0.035491534	0.329254136
Infant age	7.117348647	0.02847655	0.329254136
Diet group:Infant age	5.684754638	0.058286934	0.337039819
Taurine and hypotaurine metabolism			
Diet group	0.06471444	0.799194021	0.886514605
Infant age	2.394188207	0.302070724	0.516145463
Diet group:Infant age	3.317726288	0.190355264	0.416916929

Terpenoid backbone biosynthesis			
Diet group	0.066012556	0.797233516	0.886514605
Infant age	0.910954324	0.634145306	0.769346091
Diet group:Infant age	3.760943508	0.152518138	0.385635291
Thiamine metabolism			
Diet group	0.888866866	0.345784561	0.548555062
Infant age	0.1000417	0.951209592	0.969921912
Diet group:Infant age	1.478393917	0.477497212	0.656408062
Toluene degradation			
Diet group	3.692975401	0.054642059	0.337039819
Infant age	2.845241003	0.241081434	0.45302916
Diet group:Infant age	3.010363873	0.221976904	0.440324269
Transcription factors			
Diet group	0.690794842	0.405894157	0.604342885
Infant age	4.624822594	0.099022192	0.344354957
Diet group:Infant age	3.630408575	0.162804646	0.390982586
Transcription machinery			
Diet group	0.052202704	0.819273795	0.890888637
Infant age	2.569644955	0.2766997	0.493143877
Diet group:Infant age	2.979217089	0.225460896	0.442524575
Transcription related proteins			
Diet group	3.938132904	0.04720314	0.337039819
Infant age	7.48296936	0.023718862	0.329254136
Diet group:Infant age	7.932320248	0.018946044	0.329254136
Transfer RNA biogenesis			
Diet group	0.020872687	0.885126321	0.923739214
Infant age	0.619989717	0.733450727	0.850902029
Diet group:Infant age	2.733224236	0.254969304	0.470118095
Translation factors			
Diet group	0.003450242	0.953160186	0.97032019
Infant age	0.277852967	0.870292007	0.922183353
Diet group:Infant age	4.196134761	0.122693319	0.359777503
Translation proteins			
Diet group	1.559500974	0.211738302	0.43900408
Infant age	4.564412346	0.102058799	0.344354957
Diet group:Infant age	3.152212309	0.206778697	0.433051682
Transport			
Diet group	3.271332976	0.070500318	0.343852775
Infant age	8.658657983	0.013176386	0.329254136
Diet group:Infant age	7.498358979	0.02353705	0.329254136
Transporters			
Diet group	2.150709404	0.142504032	0.379703033
Infant age	4.095217981	0.129043078	0.364269514

Diet group:Infant age	4.687994636	0.095943355	0.344354957
Tropane, piperidine and pyridine alkaloid biosynthesis			
Diet group	2.876356897	0.08988918	0.344354957
Infant age	6.499364045	0.038786539	0.333662958
Diet group:Infant age	3.485016297	0.175080721	0.396000758
Tryptophan metabolism			
Diet group	1.500222275	0.220637164	0.440324269
Infant age	4.577436343	0.101396351	0.344354957
Diet group:Infant age	4.278357596	0.117751501	0.359777503
Two-component system			
Diet group	2.289662834	0.130238212	0.364901658
Infant age	6.497989956	0.038813196	0.333662958
Diet group:Infant age	5.4838925	0.064444799	0.342608231
Tyrosine metabolism			
Diet group	2.834168964	0.092278528	0.344354957
Infant age	6.91485495	0.03151072	0.329254136
Diet group:Infant age	1.576073514	0.454736678	0.6427644
Ubiquinone and other terpenoid-quinone biosynthesis			
Diet group	2.775879661	0.095694069	0.344354957
Infant age	5.348002926	0.068975669	0.343222928
Diet group:Infant age	4.600968759	0.100210292	0.344354957
Ubiquitin system			
Diet group	4.707439854	0.030032344	0.329254136
Infant age	9.55407594	0.008420905	0.329254136
Diet group:Infant age	5.369481851	0.068238871	0.342608231
Valine, leucine and isoleucine biosynthesis			
Diet group	2.049386655	0.152267521	0.385635291
Infant age	2.119196199	0.346595079	0.548555062
Diet group:Infant age	3.497266736	0.174011591	0.395019013
Valine, leucine and isoleucine degradation			
Diet group	3.58044024	0.058463656	0.337039819
Infant age	5.972553155	0.050475027	0.337039819
Diet group:Infant age	4.732592465	0.093827599	0.344354957
Vancomycin resistance			
Diet group	1.215099871	0.270324452	0.483166119
Infant age	0.323619001	0.850603229	0.905950699
Diet group:Infant age	1.208497268	0.546484883	0.716533921
Various types of N-glycan biosynthesis			
Diet group	2.362809924	0.12425819	0.359777503
Infant age	3.549189118	0.169552179	0.393340256
Diet group:Infant age	1.32496738	0.515569229	0.694883834
Vitamin B6 metabolism			
Diet group	0.025183679	0.873910231	0.922872944

Infant age	0.07184195	0.964716527	0.978880392
Diet group:Infant age	0.250145127	0.882432868	0.923739214
Xylene degradation			
Diet group	4.138963881	0.041906812	0.333662958
Infant age	1.291479237	0.524274635	0.702799188
Diet group:Infant age	2.293242954	0.317708343	0.528177462
Zeatin biosynthesis			
Diet group	1.525238674	0.216828444	0.440324269
Infant age	4.271816668	0.118137234	0.359777503
Diet group:Infant age	6.165587765	0.045831031	0.337039819
